# *Babesia duncani* multi-omics identifies virulence factors and drug targets

**DOI:** 10.1038/s41564-023-01360-8

**Published:** 2023-04-13

**Authors:** Pallavi Singh, Stefano Lonardi, Qihua Liang, Pratap Vydyam, Eleonora Khabirova, Tiffany Fang, Shalev Gihaz, Jose Thekkiniath, Muhammad Munshi, Steven Abel, Loic Ciampossin, Gayani Batugedara, Mohit Gupta, Xueqing Maggie Lu, Todd Lenz, Sakshar Chakravarty, Emmanuel Cornillot, Yangyang Hu, Wenxiu Ma, Luis Miguel Gonzalez, Sergio Sánchez, Karel Estrada, Alejandro Sánchez-Flores, Estrella Montero, Omar S. Harb, Karine G. Le Roch, Choukri Ben Mamoun

**Affiliations:** 1 Department of Internal Medicine, Section of Infectious Diseases, Yale School of Medicine, New Haven, CT USA; 2 Department of Computer Science and Engineering, University of California, Riverside, CA USA; 3 Department of Statistics, University of California, Riverside, CA USA; 4 Department of Molecular, Cell and Systems Biology, University of California, Riverside, CA USA; 5 Institut de Biologie Computationnelle (IBC), and Institut de Recherche en Cancérologie de Montpellier (IRCM - INSERM U1194), Institut régional du Cancer Montpellier (ICM) and Université de Montpellier, Montpellier, France; 6 Parasitology Reference and Research Laboratory, National Centre for Microbiology, Instituto de Salud Carlos III, Majadahonda, Madrid, Spain; 7 Reference and Research Laboratory on Food and Waterborne Bacterial Infections, National Centre for Microbiology, Instituto de Salud Carlos III, Majadahonda, Madrid, Spain; 8 Unidad Universitaria de Secuenciación Masiva y Bioinformática, Instituto de Biotecnología, Universidad Nacional Autónoma de México, Cuernavaca, México; 9 Department of Biology, University of Pennsylvania, Philadelphia, PA USA

**Keywords:** Microbiology, Applied microbiology

## Abstract

Babesiosis is a malaria-like disease in humans and animals that is caused by *Babesia* species, which are tick-transmitted apicomplexan pathogens. *Babesia duncani* causes severe to lethal infection in humans, but despite the risk that this parasite poses as an emerging pathogen, little is known about its biology, metabolic requirements or pathogenesis. Unlike other apicomplexan parasites that infect red blood cells, *B. duncani* can be continuously cultured in vitro in human erythrocytes and can infect mice resulting in fulminant babesiosis and death. We report comprehensive, detailed molecular, genomic, transcriptomic and epigenetic analyses to gain insights into the biology of *B. duncani*. We completed the assembly, 3D structure and annotation of its nuclear genome, and analysed its transcriptomic and epigenetics profiles during its asexual life cycle stages in human erythrocytes. We used RNA-seq data to produce an atlas of parasite metabolism during its intraerythrocytic life cycle. Characterization of the *B. duncani* genome, epigenome and transcriptome identified classes of candidate virulence factors, antigens for diagnosis of active infection and several attractive drug targets. Furthermore, metabolic reconstitutions from genome annotation and in vitro efficacy studies identified antifolates, pyrimethamine and WR-99210 as potent inhibitors of *B. duncani* to establish a pipeline of small molecules that could be developed as effective therapies for the treatment of human babesiosis.

## Abstract (web summary)

Comprehensive multi-omics of *Babesia duncani* reveals evolution, drug sensitivity and virulence.

## Main

Recent reports suggest an increase in the incidence of tick-borne bacterial, parasitic and viral infections worldwide^1^. This is largely due to changes in environmental factors that led to the expansion of the geographical distribution of the tick vectors, several of which carry multiple human pathogens^1^. Among these pathogens, *Babesia* parasites are considered a serious threat to humans and animals^2,3^. All cases of human babesiosis reported in the United States have been linked to either *Babesia microti* (majority of cases), *B. duncani* or a *B. divergens*-like species called MO1^3^. Available data suggest that *B. duncani* is transmitted by the hard tick *Dermacentor albipictus*^4^. The first clinical isolate of *B. duncani* (WA1) was collected in 1991 from a patient who lacked all of the typical risk factors for fulminating babesiosis (that is, old age, splenectomy and weak immune system)^5^. Previous phylogenetic analyses based on the 18S rRNA gene and the mitochondrial *cox-1* genes revealed that *B. duncani* belongs to Clade II of piroplasmids and defines a new lineage that is closely related to *Babesia negevi*, a causative agent of canine babesiosis. This clade is distinct from those encompassing *B. microti* (Clade I), *B. bovis* (Clade VI), *Theileria* spp. (Clade IV and V) and *Cytauxzoon* spp. (Clade III)^6^. Recent studies using a continuous in vitro culture of the parasite in human erythrocytes showed that *B. duncani* has low susceptibility to atovaquone, azithromycin, clindamycin and quinine^7^. Consistent with the severe clinical outcome of *B. duncani* infection in humans^5,8–11^, studies in immunocompetent hamsters, and both immunocompetent and immunocompromised mice confirmed the high virulent status of *B. duncani*^12^.

Despite the highly virulent properties of *B. duncani*, little is known about its biology, evolution and mechanism of virulence. Here we report presumably the first completed nuclear genome sequence, assembly, three-dimensional (3D) structure and transcriptional annotation of this parasite. We also characterized its epigenomic landscape and localized the active and inactive histone marks. Our analysis further revealed that the parasite has evolved new classes of multigene families (MGFs). Drug target mining and in vitro efficacy studies identified the antifolates, pyrimethamine and WR-99210, as excellent inhibitors of parasite development within human erythrocytes. The high potency of these compounds provides promise for the future development of more effective therapies.

## Results

### *B. duncani* genome sequencing and assembly

**Fig. 1:**
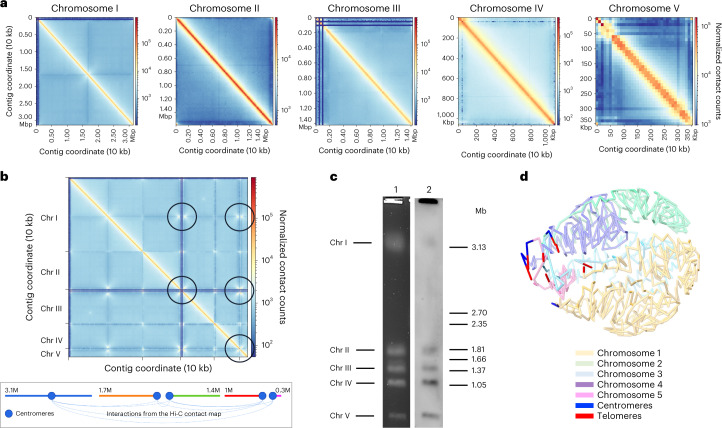
*B. duncani* genome organization. **a**, Hi-C interchromosomal contact maps for the five *B. duncani* chromosomes (10 Kb bins). **b**, Full Hi-C contact map for *B. duncani* (10 Kb bins); black circles highlight strong intra-chromosomal interaction, which are summarized in the bottom panel; X-shaped interactions typically indicate the presence of centromeres. **c**, PFGE (lane 1) and subsequent Southern-blot analyses using a *Plasmodium berghei* telomeric probe (lane 2) showing the number and approximate size of *B. duncani* Chr: ~3.1 Mb, (Chr I), ~1.81, Mb (Chr II), ~1.37 Mb (Chr III), ~1.05 Mb (Chr IV) and <1 Mb (Chr V). *Hansenula wingei* DNA chromosomes were used as DNA markers. The manufacturer’s estimates of the sizes of chromosomes are indicated in megabase pairs (Mb) on the right. The experiment was performed in biological duplicates. **d**, 3D genome structure of *B. duncani* derived from the contact map interactions. Source data

**Fig. 2:**
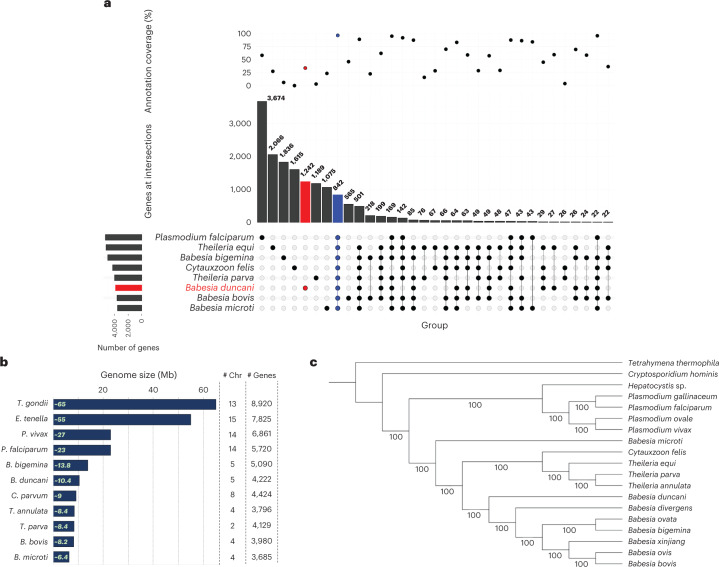
Evolutionary analysis of *B. duncani* genome. **a**, Upset plot for orthology results between *B. duncani* and other organisms. Top: the percentage of annotated proteins for shared or unique ones from a given organism is presented. Middle: the total number of shared proteins or unique ones from a given organism is depicted. Bottom: the intersection or uniqueness of a given species, with horizontal bars at the left side representing the total number of genes for a given species. **b**, Schematic showing genome sizes in megabases of various Apicomplexan species. Gene numbers include both coding and non-coding genes. **c**, Maximum-likelihood gene orthology-based phylogenetic tree analysis using single-copy orthologues from *B. duncani* and other species. Bootstrap values are shown at each node.

**Extended Data Fig. 1:**
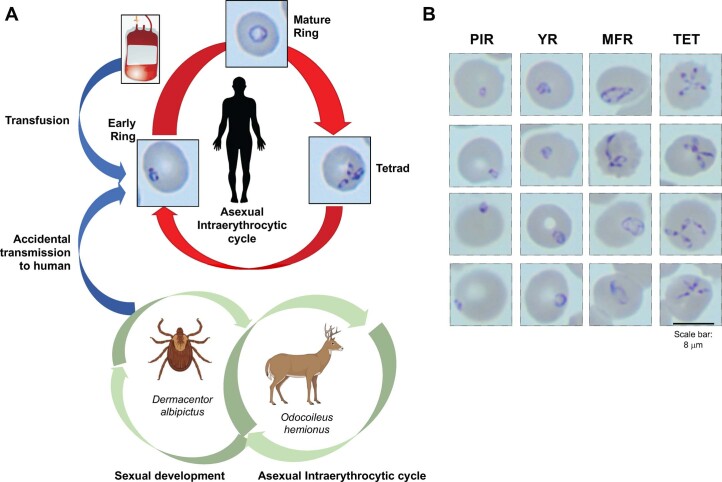
Life-cycle of *B. duncani*. **The** ***B. duncani*** **life cycle. A**. Schematic representation of the life cycle of *B. duncani* in humans, tick vector and mule deer. **B**. Representative images of the various forms of *B. duncani* grown in human erythrocytes *in vitro*. PIR: Post-invasion rings; YR: young rings; MFR: mature and filamentous rings; TET: tetrads. The representative images from three independent experiments performed in duplicates.

*Babesia duncani* asexual development takes place within human erythrocytes or those of the reservoir host (the mule deer *Odocoileus hemionus*), whereas its sexual cycle occurs in the tick vector (*Dermacentor albipictus*) (Extended Data Fig. 1)^3,4^. The parasite can be transmitted from ticks to humans either through a tick-bite or through transfusion of *Babesia duncani*-infected blood (Extended Data Fig. 1a). During its intraerythrocytic life cycle, *B. duncani* undergoes various morphological changes that manifest in four developmental stages: paired pyriform, young rings, mature and filamentous rings and tetrads (Extended Data Fig. 1b). To gain further insight into the genomic content and structure of *B. duncani*, we purified its total genomic DNA from a culture of the parasite in human erythrocytes^7^. A high-quality genome assembly was produced by processing PacBio HiFi WGS long reads (~150x coverage) and Illumina WGS paired-end reads (~130x coverage) (Supplementary Fig. 1). Reads enriched for nuclear DNA reads were assembled with HiCANU^13^. The assembly was polished using Polypolish^14^ and scaffolded/corrected using a Bionano optical map (Supplementary Fig. 2 and Table 1). The final assembly had five chromosome-level scaffolds of 3.13 Mb (Chromosome (Chr) I), 1.58 Mb (Chr II), 1.42 Mb (Chr III), 1.07 Mb (Chr IV) and 0.35 Mb (Chr V) with a total of only 35 Kb in two gaps (see Extended Data Table 1 for other statistics). The assembly pipeline is illustrated in Supplementary Fig. 1 and is described in more details in Methods. Analysis using BUSCO v5 (ref. ^15^) was used to determine that the assembly has 95.1% of the gene models in the apicomplexa_odb10 database (85.2% single-copy and 9.9% duplicated). Long-range chromatin contact frequency information using Hi-C allowed creation of contact maps for *B. duncani* WA1 (shown on Figs. 1a and 2b). The contact maps show that the assembly does not contain large mis-joints or mis-assemblies and is consistent with that of other apicomplexan parasites^16^. The centromeres interact strongly with each other (as illustrated in Fig. 1b(bottom)). Chr I was found to have a metacentric centromere; Chr II and III have a telocentric profile, and Chr IV and V have acrocentric centromeres (Supplementary Fig. 3). A similar organization was previously observed in three of the four chromosomes of *Babesia bovis*^17^.

The chromosomal organization of the *B. duncani* nuclear genome was further validated using pulsed field gel electrophoresis (PFGE) analysis. Five bands with approximate sizes of ~3.1 Mb (Chr I), ~1.81 Mb (Chr II), ~1.37 Mb (Chr III), ~1.05 Mb (Chr IV) and <1 Mb (Chr V) were identified (Fig. 1c, lane 1). Using a specific telomeric probe from *Plasmodium berghei*, each of the 5 chromosomes were labelled using Southern-blot analysis (Fig. 1c, lane 2). Altogether, these results demonstrate that *B. duncani* WA1 nuclear genome consists of five chromosomes. A 3D model was constructed from Hi-C contact maps showing genome-wide chromatin organization (Fig. 1d). The five chromosomes showed strong interactions among the centromeres (Fig. 1b,d), an organization similar to that of recently reported 3D structures of apicomplexan parasites including *Babesia microti*^16,18^. Full interactions were observed between the telomere ends, whereas only partial interactions were detected between the centromeres and telomeres (Fig. 1d).

### *B. duncani* genome annotation

To accurately predict gene loci, we generated Illumina RNA-seq and PacBio IsoSeq data using RNA isolated from parasites propagated in vitro in human erythrocytes. The gene annotation pipeline used FunAnnotate and InterProScan to determine gene loci and functional annotations. The final set of gene models included 4,222 gene loci (including 52 transfer (t)RNA genes) with an average length of 1,656 bp (average protein length was 499 amino acids) (Extended Data Table 2). Of the total genes, 63% were multi-exon and 76% had a functional annotation. The *B. duncani* nuclear genome expresses two 18S rRNA encoding genes, one on Chr I and the second on Chr III, three 5S rRNAs genes in tandem on Chr II, and 46 tRNAs distributed on all five chromosomes with another 6 found in unplaced contigs. Transcript analysis using BUSCO v5 (ref. ^15^) showed that the transcripts’ annotations match 88.6% of the transcripts in the apicomplexa_odb10 database (80.5% single-copy and 8.1% duplicated), whereas 5.8% are fragmented and 5.6% are missing. BUSCO analysis further showed that the *B. duncani* protein annotations match 87.7% of the proteins in the apicomplexa_odb10 database (79.6% single-copy and 8.1% duplicated), whereas 5.6% are fragmented and 6.7% are missing. Only 1.2% of the genome is considered repetitive by RepeatMasker.

### Genome-wide comparison of *B*. *duncani* gene models with other Apicomplexa

The *B. duncani* nuclear genome has a GC content of 37.32%, which is similar to that of other piroplasmids^19^ and almost double that of *Plasmodium falciparum* (19.34%)^20^. Main genome and gene statistics of *B. duncani* and other Apicomplexa are shown in Extended Data Table 2. The genome size, number of chromosomes and number of protein coding genes in *B. duncani* are higher than those in *B. microti, B. bovis, Theileria. parva, Cryptosporidium. parvum* and *Theileria. annulate*, which could be also attributed to a more complete genome assembly for *B. duncani*. An approximate 60% of the *B. duncani* genome encodes proteins, with ~63% of the genes containing introns with an average number of exons per gene of 3.1.

Out of the total 4,222 *B. duncani* genes, 3,484 mapped evenly across the five chromosomes while the rest were located on unplaced contigs. A summarized comparative genomics analysis between *B. duncani* and other Apicomplexa species is presented in Fig. 2a. The bars depict the number of orthologous proteins shared between the compared species, as well as unique proteins for each organism and their percentage of annotation (upper dot plot). Interestingly, we found 842 core proteins that are shared between *B. duncani* and other Apicomplexa, where 100% of the proteins have a functional annotation. Interestingly, the *B. duncani* genome possesses 1,242 unique proteins only ~30% of which have a functional annotation, suggesting that there could be several predicted proteins with unknown functions. In contrast, *B. bovis* has 565 unique proteins, ~50% of which are annotated, and *P. falciparum* has 3,674 proteins, ~55% of which are annotated. These differences could be due to different genome size (as seen in Fig. 2b), quality of assembly and annotation, gene paralogy or species-specific related proteins.

**Extended Data Fig. 2:**
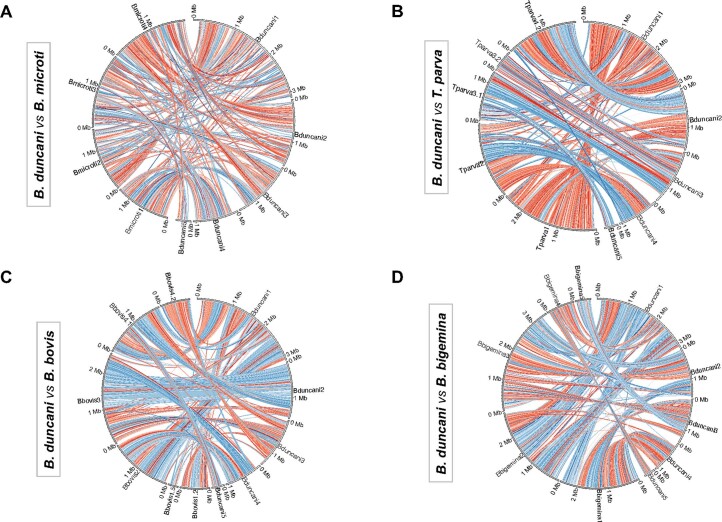
Synteny circos plots. **Circos synteny plots**. The chromosomes of *B. duncani* are illustrated on the right semicircle on all circular plots, and the chromosomes of the other organisms are on the left semicircle (**A**: *B. microti*, **B**: *T. parva*, **C**: *B. bovis*, **D**: *B. bigemina*); blue arcs indicate syntenies, red arcs indicate syntenies involved in a reversals; the intensity of the color is proportional to the level of collinearity; the number after the species’ name refers to the chromosome number (when chromosomes are broken into pieces, fragments.

To investigate the evolutionary distance and relationships among piroplasms, the phylogeny for 19 species (18 different Apicomplexa with *Tetrahymena hominis* as the outgroup) was determined using a maximum-likelihood approach and 190 single-copy orthologous proteins (see Methods). As shown in Fig. 2c, *B*. *duncani* is a defining member of a separate clade in Piroplasmida. This analysis provided further support to previous clade classification of piroplasms^21^ using other genetic markers, which showed that *B. duncani* belongs to a clade (Clade II) distinct from that of *B. microti* (Clade I)^22^ and other human *Babesia* parasites (Fig. 2c). *B. duncani* also shares the same ancestor with *Babesia* species of Clade VI, which includes *B. bovis, B. bigemina* and *B. divergens* (Fig. 2c). Synteny-based analyses (Extended Data Fig. 2) indicated that *B. duncani* is least sytenic with *B. microti* compared with *T. parva, B. bovis* and *B. bigemina* (Extended Data Fig. 2).

The metabolic atlas of *B. duncani* was reconstituted from the annotated genome. All enzymes of the glycolytic pathway and the tricarboxylic acid cycle were found in the annotated proteome (Extended Data Tables 3 and 4). An estimated 11.4% of the coding sequences harbour an N-terminal signal peptide, thus highlighting the importance of protein secretion in *B. duncani* intraerythrocytic development. Of all the predicted proteins of *B. duncani*, 2.5% of coding sequences carry a mitochondrial targeting sequence, 19% have at least one predicted transmembrane domain and 0.1% have 18 transmembrane domains. The most conserved genes between *B. duncani* and other *Babesia* species are those involved in DNA replication, transcription and protein translation. Our analysis identified 19 members of the Apicomplexan Apetala 2 (ApiAP2) family and 17 GPI-anchored proteins (Extended Data Tables 5 and 6, and Supplementary Fig. 4).

### New multigene families of *B. duncani*

**Fig. 3:**
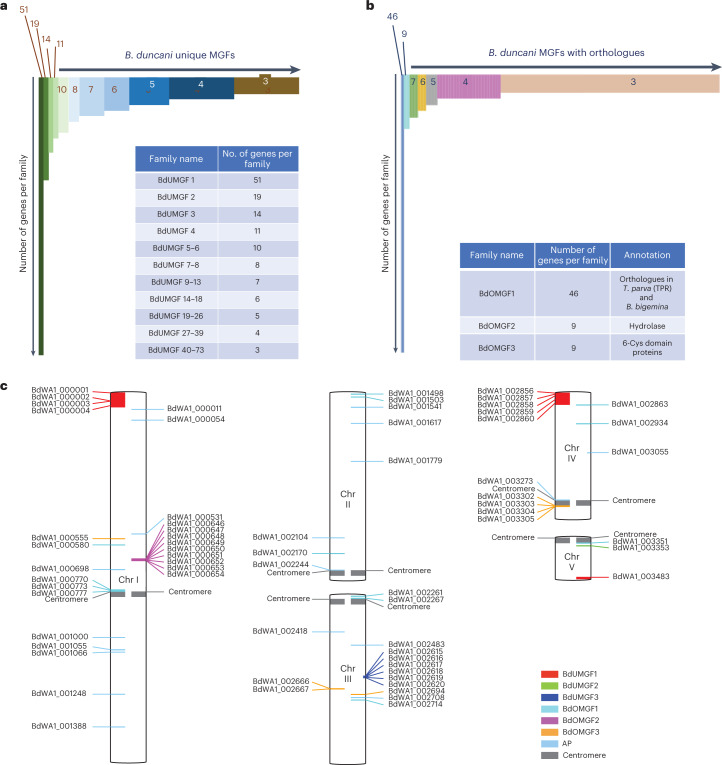
Multigene families and chromosomal localization in the *B. duncani* genome. **a**, Gene families unique to *B. duncani*. **b**, Gene families with orthologues in other Apicomplexa. TPR, *Theileria parva* repeat gene family. **c**, Localization of the genes in the gene families BdUMGF1, BdUMGF2, BdUMGF3, BdOMGF1, BdOMGF2, BdOMGF3 and AP on the five *B. duncani* chromosomes (genes localized on unplaced contigs are ignored); genes on the right side of a chromosome are on the positive strand, genes on the left side are on the negative strand.

**Fig. 4:**
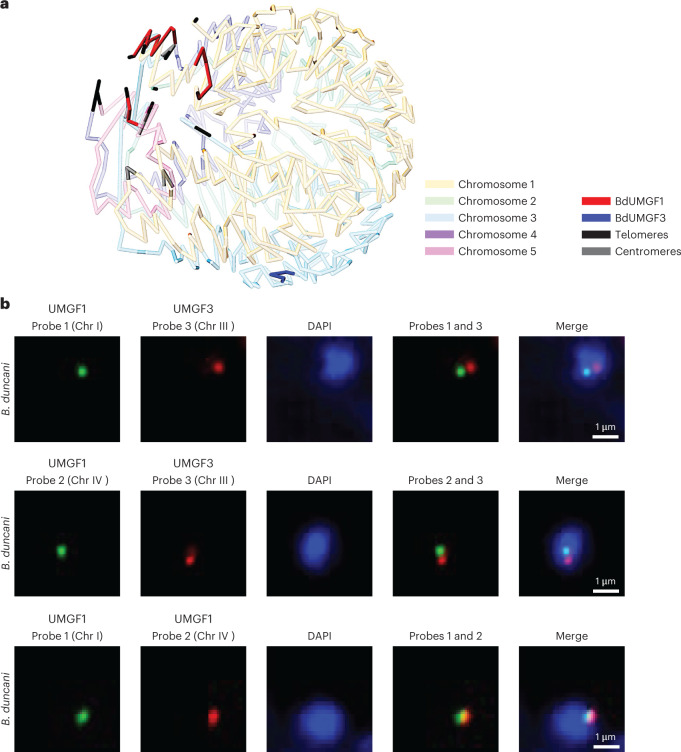
*B. duncani* unique multigene family (BdUMGF) members are spatially distinct from each other in the genome. **a**, *B. duncani* WA1 3D genome model showing the location of BdUMGF1 (red) and BdUMGF3 (dark blue) on different chromosomes. **b**, The detection of BdUMGF1 (located on Chr I and Chr IV) and BdUMGF3 (located on Chr III) using a DNA probe labelled with either fluorescein (Probes 1 and 2, green) or biotin/avidin-rhodamine (Probe 3, red). The detection of BdUMGF1 on Chr I with DNA probe labelled with fluorescein (Probe 1) and on Chr IV using a DNA probe labelled with biotin/avidin-rhodamine (Probe 2, red). Nuclear DNA is stained with DAPI (blue). Representative images from two independent experiments performed in duplicates.

The extremely high virulence of *B. duncani* and the cytokine storm it triggers in immunocompetent mice and hamsters suggest that the parasite produces virulence factors during its blood stage development that are delivered into the host and trigger a strong host response^12^. Interestingly, analysis of the *B. duncani* genome identified 747 genes that belong to MGFs, each with at least three members (Fig. 3). These families can be divided into two classes: BdUMGFs (unique to *B. duncani*) and BdOMGFs (with orthologues in other Apicomplexa) (Fig. 3a,b). The BdUMGF class includes 397 genes grouped into 73 gene families, whereas the BdOMGFs comprise 420 genes grouped into 105 gene families, each belonging to an orthologous group among Apicomplexa (Fig. 3b). The components of each of the gene families and their properties are available in the supplementary datasets. The chromosomal location of the genes in the three largest BdUMGF and BdOMGF families, as well as the activator protein (AP) family, is illustrated in Fig. 3c. Our analysis showed that genes in the BdUMGF1 strongly co-localize and are on the telomeres of Chr I and IV, which also appear to be in close proximity in the 3D genome model (Fig. 3c and also Fig. 4a and Supplementary Fig. 4). All genes in the BdUMGF3 family (shown in blue) are clustered together on the same strand in the middle of Chr III. All genes in the BdOMGF2 family (shown in magenta) are clustered together on the same strand in the middle of Chr I. The genes in the AP family are uniformly distributed along the chromosomes.

The distribution of BdUMGF1 gene family members near the telomeres as determined by Hi-C analysis (Fig. 4a) suggests a possible heterochromatin clustering associated with transcriptional silencing surrounding these genes as described for the *var* or *SICAvar* gene families of *P. falciparum* and *P. knowlesi*, respectively^23,24^. The 3D genome model of the *B. duncani* genome based on Hi-C analysis also determined that members of the BdUMGF3 multigene family are present outside the telomeric and centromeric regions (Fig. 4a). Interestingly, despite this localization, RNA-seq showed that these genes are repressed (Supplementary Data file 1). DNA fluorescence in situ hybridization (FISH) using probes specific to BdUMGF1 to mark Chr I and IV, and a probe specific to BdUMGF3 to mark Chr III demonstrated that BdUMGF1 and BdUMGF3 are distant from each other in the nucleus since two distinct non-overlapping signals were observed for each family (Fig. 4b and Supplementary Fig. 5). In contrast, the members of BdUMGF1 on Chr I and IV are in close proximity to each other as two partially overlapping signals were observed using DNA FISH (Fig. 4b and Supplementary Fig. 5). These data suggest that the transcriptional repression of the BdUMGF3 family is not due to their proximity to the members of BdUMGF1 family (Fig. 4a,b).

### Regulation of gene expression in *B. duncani*

**Fig. 5:**
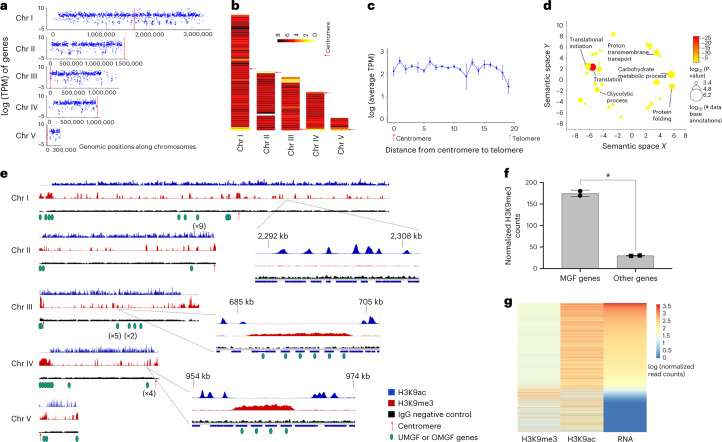
Transcriptomic and epigenomic profiles of *B. duncani*. **a**, Logarithms of the TPM counts were used as expression values0 for each gene across the 5 chromosomes using the R package ggplot2. **b**, RNA-seq data as normalized heat maps across the 5 chromosomes. Chromosomes were divided into 50 kb bins and the average of the log TPM of genes within each bin was calculated. **c**, Relation between gene expression and distance from the centromeres to the telomeres. Chromosomes were divided into 20 bins. For each bin, the average gene expression value was plotted. Error bars denote the range of expression values within each bin for each chromosome. *n* = 2 biologically independent samples. **d**, Gene ontology (GO) enrichment of the most highly expressed genes. GO was calculated using the R package TopGO with the weight01 algorithm and the biological process tree. REVIGO was used to visualize the GO results of the most highly expressed genes (500 TPM or higher). Fisher’s exact test, one-sided, with the weight01 algorithm. **e**, Heterochromatin and euchromatin distribution across all five *B. duncani* chromosomes. Tracks correspond to H3K9me3-ChIP, H3K9ac-ChIP and IgG control and are normalized to millions of mapped reads. **f**, Normalized H3K9me3 counts in multigene families, and other genes encoded in the *B. duncani* genome (unpaired *t*-test with Welch’s correction, *P* = 0.022). *n* = 2 biologically independent samples. **g**, Comparison between epigenetic marks and gene expression. Heat maps were generated using normalized log_2_ H3K9me3 and H3K9ac read counts as well as the RNA-seq TPM levels for each gene. Read counts for H3K9me3 and H3K9ac were normalized to millions of mapped reads and gene length, while TPM was determined by Stringtie. Genes were sorted from high to low TPM highlighting the correlation and anti-correlation between transcript abundance and the H3K9ac and H3K9me3 marks, respectively.

Using transcript abundance values based on the mean depth of coverage (see Methods), we found that the total range of transcriptional activity captured using the continuous in vitro growth conditions varied by more than four orders of magnitude (Fig. 5a–d and supplementary datasets). Overall, RNA-seq data captured close to 90% of the predicted annotated genes in the assembled *B. duncani* genome (Fig. 5a,b), indicating that most genes are expressed during the intraerythrocytic stages of the parasite life cycle and are potentially needed for parasite survival within host erythrocytes. We further examined the possible relationship between gene expression and genome organization. The genes of *B. duncani* were binned into 20 groups on the basis of their distance from the centroid of telomeres. The average gene expression using RNA-seq data was calculated (Fig. 5c) and the normalized average gene expression values across the whole chromosomes were colour-coded (Fig. 5b). Similar to what was observed in the *P. falciparum* genome that possesses gene families involved in antigenic variation, we detected a significant relationship between gene expression and 3D location relative to the telomeres, with noticeable repression near the telomeric ends of Chr I, IV and V, which harbour gene clusters belonging to the BdUMGF1 (Fig. 3c), and suggests that the *B. duncani* genome may contain a heterochromatin cluster near the telomere ends allowing for mono-allelic expression of the BdUMGF1.

Our analysis revealed that among the most highly expressed genes are genes involved in protein translation, protein degradation, cell cycle, ion transport, carbohydrate metabolism and histone core proteins (Fig. 5d).

### Epigenomic landscape of *B. duncani*

The expression profile of the *B. duncani* multigene families is reminiscent of that of the *P. falciparum var* gene family, which consists of ~60 genes that encode a major virulence antigen, PfEMP1, with a single variant expressed in each infected red blood cell^25^. PfEMP1-mediated antigenic variation has been suggested to play a role in *P. falciparum* immune evasion^26^. The control of mutually exclusive *var* gene expression in the parasite relies on epigenetic and chromatin structural changes that are critical for pathogenesis and immune evasion^27,28^. To assess whether members of the BdUMGF family undergo similar epigenetic regulation and thus could undergo antigenic variation, we conducted chromatin immunoprecipitation assays (ChIP) to identify both the specific histone marks and the affected genes. ChIP was conducted using antibodies against tri-methylated histone 3 lysine 9 (H3K9me3) and acetylated histone 3 lysine 9 (H3K9ac) as markers for heterochromatin and euchromatin marks, respectively. The immuno-precipitated DNA and input used as a positive control were purified, amplified and subjected to next-generation sequencing on the Illumina Novaseq sequencing platform. Reads were mapped to the *B. duncani* genome and normalized per million of mapped reads for each sample (Fig. 5e). Pearson correlation coefficients between each ChIP-seq sample confirmed the reproducibility of our data (Supplementary Table 2) and demonstrated that euchromatin and heterochromatin marks are mutually exclusive (Fig. 5e–g). To identify genomic regions that are associated with hetero- and euchromatin, we visualized the ChIP-seq data using the Integrative Genomic Viewer (IGV) (Fig. 5e). Similar to findings in *Plasmodium*, the heterochromatin is localized to telomeric and subtelomeric regions of all chromosomes, except for the right end of Chr I which is depleted of multigene families. Heterochromatin marks were also observed on multigene families found in internal chromosome clusters as well as in centromeric regions, a profile similar to that observed in *T. gondii*^29^. Statistical analyses were then used to determine whether genes from multigene families were significantly enriched with H3K9me3 marks compared with other genes encoded in the *B. duncani* genome. Our data demonstrate that in *B. duncani*, genes that belong to multigene families (UMGFs or OMGFs) are significantly enriched in H3K9me3 marks (Fig. 5f). Additional histone H3K9me3 marks were also observed in genes throughout the genome (Fig. 5g); however, most of these genes were not expressed during the intraerythrocytic life cycle. Our analysis further identified additional genes that are annotated as hypothetical proteins as they lack any homologues in other organisms and potentially belong to novel multigene families. Some of them are localized on the right end of chromosome III and in the Hi-C experiment were shown to interact with telomeric and subtelomeric regions of the four other chromosomes, allowing possible mono-allelic expression between the BdUMGF and BdOMGF genes. The euchromatic mark, H3K9ac, on the other hand, is detected in all other chromosomal regions and found to be enriched in the promoters of active genes (Fig. 5g). Our analysis further showed that H3K9ac intensity correlates with transcript abundance (Fig. 5g). Altogether, the epigenetic analysis provides new insights into *Babesia* gene expression and regulation. This study which revealed a unique epigenetic silencing associated with multigene families in *B. duncani*, suggests that antigenic variation might occur in this parasite.

### Genome mining to identify antibabesial inhibitors

**Fig. 6:**
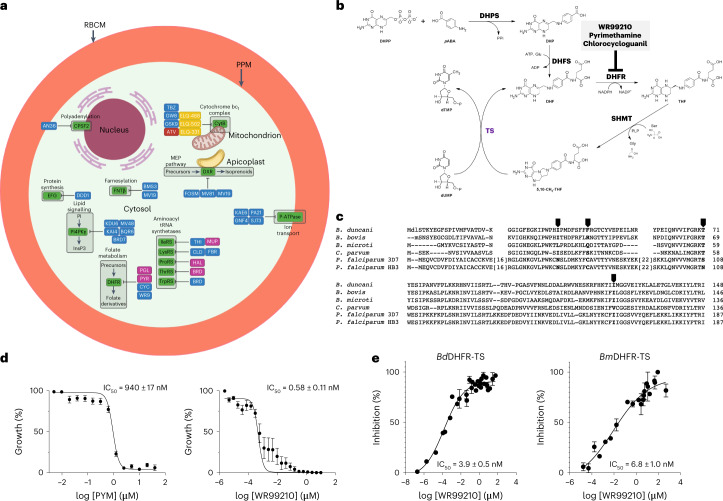
Potential targets for therapeutics development in *B. duncani*. **a**, Drugs are categorized as follows: Red, effective against *Babesia* parasites and used clinically; Yellow, effective against *Babesia* parasites but have not been evaluated clinically; Pink, clinically approved drugs for other diseases but have not been tested against *Babesia* parasites; and Blue, drugs under clinical evaluation for the treatment of other diseases but have not yet been tested against *Babesia* parasite inhibitors. Drug and protein abbreviations are as follows: Lys-TRNAS, Lysyl tRNA Synthase; Pro-TRNAS, Prolyl tRNA Synthase; Thre-TRNAS, Theronyl/alanyl tRNA Synthase; Tryp-TRNAS, Tryptophanyl tRNA Synthase; DHFR, Dihydrofolate reductase; P-ATPase, P-type ATPase; CytB, Cytochrome bc1 complex subunit 7 superfamily; Translation EFG/EF2, Elongation factor EFG (EFG); PI4PK𝛄, Phosphatidylinositol 4-kinase gamma; FNTB, Farnesyltransferase subunit beta; CPSF2, Polyadenylation specificity factor subunit 2; DXR, MEP Synthase; FBR, Febrifugine; HAL, Halofuginone; CLD, Cladosporin; MUP, Mupirocin; BRD, Borrelidin; DDD1, DDD107498; KDU6, KDU691; MV48, MMV048; KAI4, KAI4KAI407; BQR6, BQR695; BRD, BRD73842; ATV, Atovaquone; TBZ, Tetracyclic Benzothiazepine; GW8, GW844520; GSK9, GSK932121; ELQ, Endochin-like quinolones; KAE6, KAE609; GNF4, GNF-Pf-4492; PA21, PA21A092; SJ73, SJ733; AN36, AN3661; FOSM, Fosmidomycin; MV81, MMV008138; BMS3, BMS-388891; MV19, MMV019066; PYR, Pyrimethamine; PGL, Proguanil; MV27, MMV027634. **b**, Overview of folate metabolism and inhibitors. Synthesis of DHF precursor by DHP(F)S and reduction of DHF to tetrahydrofolate (THF) catalysed by the bifunctional enzyme DHFR by using NADPH as electron donor. The thymidylate synthase (TS) catalyses the reductive methylation of deoxyuridine monophosphate (dUMP) in deoxythymidine monophosphate (dTMP) using the tetrahydrofolate (5,10-Methylene THF) as cofactor via a hydroxymethyl transfer, mediated by serine hydroxy methyltransferase (SHMT). Common DHFR inhibitors are marked in grey box. **c**, Sequence comparison of DHFR-TS sequences between *B. duncani, B. microti, B. bovis* and *P. falciparum* pyrimethamine-sensitive (3D7) and resistant (HB3) parasites with highlighted resistance-associated residues in their primary sequences (black arrows). **d**, Dose-response curves with IC_50_ values of antifolates pyrimethamine and WR-99210 and their inhibitory effect on *B. duncani* intraerythrocytic parasite growth. Data presented as mean ± s.d. from three independent experiments performed in triplicates. **e**, Enzymatic activity inhibition of BdDHFR-TS and BmDHFR-TS by WR-99210. Data presented as mean ± s.d. from three independent experiments performed in triplicates.

Mining of the annotated *B. duncani* proteome identified several potential drug targets (Fig. 6a and Supplementary Table 3). One of these is dihydrofolate reductase-thymidylate synthase (DHFR-TS) (Fig. 6b). Residue T71 in *B. duncani* DHFR-TS (BdDHFR-TS) sequence is equivalent to residue 108 in DHFR-TS from *P. falciparum* (PfDHFR-TS) (Fig. 6c)^30–34^. In *P. falciparum*, the serine residue in this position is associated with susceptibility to pyrimethamine, whereas an asparagine residue is associated with resistance to this drug^35^. Consistent with our genome analysis, drug sensitivity and in vitro safety assays revealed that *B. duncani* is sensitive to pyrimethamine and WR-99210, with 50% inhibitory concentration (IC_50_) values of 940 and 0.58 nM, respectively, with both compounds showing excellent in vitro therapeutic indices when tested against four human cell lines (Fig. 6d and Extended Data Table 7). The specificity of inhibition by these drugs was further examined biochemically using recombinant DHFR-TS enzymes from *B. duncani* and *B. microti* (BmDHFR-TS) (Fig. 6e). WR-99210 was found to inhibit BdDHFR-TS and BmDHFR-TS enzymes, with IC_50_ of 3.9 and 6.8 nM, respectively. Our biochemical assay results showed that both enzymes have similar kinetic properties (*K*_m_ and V_max_), but different turnover numbers (*k*_cat_) (Extended Data Table 8).

The availability of a complete genome sequence and annotation enables the understanding of the mechanism of action of antiparasitic drugs. As proof of concept, we used a single-step drug selection approach^36^, we selected *B. duncani* parasites that are refractory to atovaquone in vitro (Supplementary Fig. 6), and their drug susceptibility was compared to that of the parental isolate WA1 (IC_50_ of 0.072 µM). Whole genome sequencing of clone BdATV^R^, which was found to be 15x more resistant to atovaquone compared with WA1 (Supplementary Fig. 6a), identified a single nucleotide polymorphism associated with the resistance profile in the form of a non-synonymous mutation L117F in the parasite *Bdcyt-B* gene, which encodes a component of the mitochondrial bc1 complex and is the main target of atovaquone and endochin-like quinolones (Supplementary Fig. 6b)^37,38^. A similar mutation was previously found to confer resistance to atovaquone in *P. falciparum*^39^. Altogether, these data highlight the advantages of genomic analyses in the search for potential new therapies for the treatment of human babesiosis and in the identification of the mechanism of action of antiparasitic drugs.

## Discussion

In this study, we report the first genome sequence assembly, 3D structure, annotation, transcriptomic profile and metabolic reconstitution of the human intraerythrocytic pathogen *B. duncani*. This parasite species is unique within Piroplasmida and belongs to Clade II^21^. The high GC content of this parasite, its ability to propagate continuously in human red blood cells in vitro and the availability of a mouse model of virulence make it an ideal system to study the basic requirements for intraerythrocytic parasitism and to identify conserved and essential metabolic processes that could be targeted for the development of pan-antiparasitic drugs. Inhibitors of such processes could then be evaluated in mice for safety and efficacy to select the best candidates to advance towards clinical evaluation. Several new targets and potential drugs that could inhibit their function have been identified in this study and will stimulate future efforts to evaluate the activity of new inhibitors using the *B. duncani* in culture-in mouse model system (Fig. 6a). As a proof of feasibility, we have validated the DHFR-TS enzyme of *B. duncani* and *B. microti* as excellent targets for the development of antibabesial drugs (Fig. 6e and Extended Data Table 8). Our study further examined the utility of the *B. duncani* genome sequence in validating the mode of action and determining possible mechanisms of resistance of known and newly developed antibabesial drugs.

Our annotation of the *B. duncani* genome provides invaluable insights into the biology, pathogenesis and drug susceptibility of this important human pathogen. Notable among these is the presence of classes of multigene families that could be important in host–pathogen interactions and virulence of the parasite in mice and humans. The BdOMGF families encompass genes with orthologues in other hematozoan parasites and thus might have evolved to support survival of the parasite in mammalian red blood cells and escape from host immune attacks as was shown for the *var* and variant erythrocyte surface antigen (*vesa*) gene families of *P. falciparum* and *B. bovis*^26,40^.

Evolutionary analysis of the *B. duncani* genome (Fig. 2) revealed its unique genetic relationships with other haemoparasites. This analysis, which confirmed the membership of *B. duncani* in clade II of Piroplasmida^21^, is also consistent with a recent phylogenomic analysis of the parasite^41^. The evolution of Piroplasm parasites could be shaped by several factors such as geography, tropism, as well as other genomic features such as genome size and chromosome number. However, our analysis using orthologous proteins with functional annotation and shared among all analysed species suggests that *B. duncani* has a distant relationship with the rest of *Babesia* species, as well as unique metabolic, virulence and pathogenic features that can be studied through the genomic information presented in this work. A classification of selected organisms and OrthoMCL groups based on the presence or absence of these orthologues is consistent with the proposed evolution of *Babesia* species and the phylogenetic position of *B. duncani*. Binary distance between OrthoMCL groups places *B. duncani* at the root of the clade of *Babesia* (Supplementary Fig. 7).

Another unique property of *B. duncani* is the presence of a large array of tandemly duplicated functions. We found 139 genes with orthology groups that contain unique genes in other piroplasmida but have two or up to four genes in *B. duncani*. These genes vary in size and sequence, suggesting that duplication events occurred early during the evolution of the parasite and continue to occur in the genome. No specific enrichment of specific functions was observed. Two DNA polymerase subunits and the RPB11 subunit of polymerase II were duplicated, as well as transcription factor IIA (TFIIA) basal factor and other RNA binding and/or modifying proteins. Duplicated functions were also associated with cellular trafficking, ubiquitinylation and stress response. No enzymes from the central metabolic pathways or subunits of the ribosome were part of this specific group of duplicated genes. We also identified 73 unique gene families in *B. duncani* (BdUMGFs) that have no homologues in other Apicomplexa based on BLAST and OrthMCL analyses (Fig. 3a). RNA-seq data indicate that some of the BdUMGFs, such as BdUMGF3, are not expressed during the asexual life cycle of the parasite and are potentially important for parasite development during other stages of the life cycle. Interestingly, transcriptional repression was observed in the case of the BdUMGF3 gene family, which are distant from the telomeres and centromeres. DNA FISH and Hi-C analyses confirmed the distribution of the BdUMGF3 gene family on Chr III at a position distinct from that of BdUMGF1 on Chr I and IV (Fig. 4).

We further performed ChIP-seq analysis in *B. duncani* to investigate genomic histone marks, which are often associated with regulated cell biological events such as gene expression, cell differentiation and antigenic variation (Fig. 5e–g). The dynamic distribution of acetyl or methyl marks on the histone tails are essential to the maintainence of the heterochromatin and euchromatin structure and are known to regulate gene expression needed for parasite survival. In the *P. falciparum* asexual blood stages, a transcriptionally silent heterochromatin is defined by the presence of H3K9me3 and is restricted to the telomeric and subtelomeric regions, as well as some internal chromosome clusters associated with multigene protein families involved in antigenic variation or genes involved in sexual differentiation^42^. Conversely, euchromatin marks are characterized by a different set of histone marks including H3K9ac localized in the promoter regions of active genes. The binding of transcription factors with their DNA binding domains is heavily dependent on the state of the chromatin structure surrounding the promoter regions. Inhibition of histone writers and erasers such as histone acetyltransferases, histone methyltransferase, histone deacetylases and histone demethylases has been shown to lead to inhibition of *Plasmodium* growth in vitro and in vivo^43–45^. Our ChIP-seq analysis in *B. duncani* revealed trends similar to those observed in *T. gondii* and *P. falciparum*^28,46^. In particular, the epigenetic profile of multigene families in *B. duncani* is reminiscent of that of *var* genes in *P. falciparum*, suggesting that a similar mechanism of antigenic variation might occur in this parasite and could account for its high virulence.

In summary, our analysis of the *B. duncani* nuclear genome and transcriptome has shed light into the biology, evolution and drug susceptibility of this important human pathogen. It is anticipated that this knowledge will help advance new strategies to develop reliable, sensitive and specific diagnostic tools as well as therapeutic strategies for better management of human babesiosis. The close relationship between *B. duncani* and other haemoparasites including malaria parasites will help future efforts to better understand the evolution of virulence in Apicomplexa.

## Methods

### Parasite strain

The *B. duncani* WA1 used in this study was obtained from BEI Resources (NR-12311) and propagated either in vitro or in mice and hamsters as previously described^12^.

### In vitro parasite culture of *B. duncani*

The in vitro *B. duncani* parasites were cultured in human red blood cells (hRBCs) as reported earlier^12^. Briefly, parasites were cultured in a complete HL-1 medium (base medium of HL-1 (Lonza, 344017) supplemented with 20% heat-inactivated FBS, 2% 50X HT Media Supplement Hybrid-MaxTM (Sigma, H0137), 1% 200 mM l-glutamine (Gibco, 25030-081), 1% 100X penicillin/streptomycin (Gibco, 15240-062) and 1% 10 mg ml^−1^ gentamicin (Gibco, 15710-072)) in 5% haematocrit A^+^ RBCs. The cultures were maintained at 37 °C under a 2% O_2_/5% CO_2_/93% N_2_ atmosphere in a humidified chamber. The culture medium was changed daily and parasitemia was monitored by light microscope examination of Giemsa-stained thin-blood smears.

### Chemicals

Unless otherwise stated, chemicals were purchased from commercial suppliers and used as received. WR-99210 was ≥95% pure as determined by reversed-phase high-performance liquid chromatography (HPLC) and was purchased from Jacobus.

### DNA prep for PacBio sequencing

Genomic DNA was isolated from 100 ml in vitro culture of *B. duncani* (15% parasitemia and 5% haematocrit) using DNeasy Blood and Tissue kit (Qiagen, 69506), and quality control and concentration determination were performed using nanodrop and qubit. DNA integrity was evaluated using Blue Pippin pulse gel and the DNA was then used for library preparation using Pacific Biosciences SMRTbell Express template prep kit 2.0 (100-938-900) according to manufacturer instructions. The Pacific Biosciences Smart Link software was used to determine loading concentration and proper stoichiometric measurements. The gDNA library was then annealed to the Pacific Biosciences V5 primer for 1 h at 20 °C. The annealed library was then bound to polymerase using Pacific Biosciences Polymerase 2.2 for 1–4 h at 30 °C and loaded onto the Sequel II Instrument as an adaptive sequencing run. At least one smart cell was sequenced for each gDNA library with a move time of 30 h and a pre-extension of 2 h. After the DNA library sequencing was completed, the loading metrics were evaluated by mean read length, polymerase read length, data yield and P1 values (a productive zero-mode waveguide (ZMW) with a high quality (HQ) sequencing region detected within the read) to ensure the sample ran as expected and data had met Yale’s gold standards (polymerase read length between 50–60 kb, data yield (HiFi) of around 2–4 million reads of a total of 10–20 Gb and P1 between 60–70%).

### DNA preparation for Bionano optical mapping

A total of 60 ml in vitro culture of *B. duncani* at 15% parasitemia and 5% haematocrit was collected and centrifuged at 500 × *g* to obtain a packed pellet. For analysis, pellet was used to isolate ultra-high molecular weight gDNA for use in genomic optical mapping by Histogenetics using the Bionano Prep Blood and Cell Culture DNA Isolation kit (Bionano Genomics, 80004). Following this, DNA was quantified using Qubit dsDNA BR Assay kit. A total of 0.75 g of high molecular weight DNA was then labelled using the Bionano Prep direct label and stain method (Bionano Genomics, 80005) and loaded onto a flow cell to run on the Saphyr optical mapping system (Bionano Genomics). Approximately 1,177 Gb of data were generated per run. Raw optical mapping of molecules in the form of BNX files were run through a preliminary bioinformatics pipeline that filtered out molecules less than 150 kb in size and less than 9 motifs per molecule to generate a de novo assembly of the genome maps.

### Genome sequencing and assembly

An in vitro culture of *B. duncani* (100 ml) in hRBCs was propagated to a parasitemia of 10% at 5% haematocrit. DNA was isolated from infected hRBCs and sequenced at the Yale Center for Genome Analysis using Illumina HiSeq 2500 and PacBio HiFi (CCS) sequencing. A total of 177,189 PacBio HiFi reads were obtained. The average HiFi read length was 8,814 bp, and the longest read was 29,067 bp. The HiFi reads totalled 1.56 B bases, which translated to an expected ~156x coverage of the *B. duncani* genome (assuming a ~13 Mb genome). HiFi reads were mapped using Minimap2^47^ to the *B. duncani* mitochondrion and apicoplast. Only 0.41% of the reads mapped to these organelles, which were then discarded to enrich the data for nuclear DNA. HiFi reads were also mapped to the human genome to determine possible host contaminations, but only 1.48% of them were flagged. Cleaned reads were assembled using HiCANU v2.2^13^ using default parameters. HiFi reads were also tested on HiFiASM v0.16^48^ and Wengan^49^, but they generated slightly inferior assemblies (based on assembly statistics, comparison with the optical map and transcript isoform annotations). The Bionano Hybrid Scaffolding pipeline v1.7 was used to compare the draft assembly with the Bionano optical map to detect possible mis-joins and create scaffolds. The scaffolded assembly was polished using two rounds of PolyPolish^14^. At every step of the assembly (draft, scaffolds, polished), a series of quality control steps were carried out to ensure that no imperfections were introduced. The quality control steps included (1) mapping of all the Illumina WGS and HiFi reads to the assembly, (2) carrying out a BUSCO^15^ completeness analysis (at the genome level) and (3) determining the number of detected gene loci from the mapping of RNA-seq IsoSeq reads.

### DNA preparation for Hi-C

An in vitro culture of *B. duncani* WA1 (100 ml) in A^+^ hRBCs was collected at parasitemia of 15% and 5% haematocrit. The culture was centrifuged at 500 × *g*, and the pellet was crosslinked in 1.25% formaldehyde for 25 min at 37 °C. Crosslinking was quenched in a final concentration of 150 mM glycine for 15 min at 37 °C, followed by a 15 min incubation at 4 °C. Parasite pellets were then resuspended in lysis buffer (10 mM Tris-HCl, pH 8.0, 10 mM NaCl, 2 mM 4-(2-aminoethyl) benzenesulfonyl fluoride HCl, 0.25% Igepal CA-360 (v/v) and EDTA-free protease inhibitor cocktail (Roche)), and incubated on ice for 30 min. Nuclei were isolated after homogenization by 15 gauge needle passages. In situ Hi-C protocol was conducted as previously described^50^. Briefly, nuclei were permeabilized using 0.5% SDS; the DNA was digested with 100 units of Mbol (NEB), and the ends of restriction fragments were filled using biotinylated nucleotides and ligated using T4 DNA ligase (NEB). After reversal of crosslinks, the ligated DNA was purified and sheared to a length of ~300–500 bp using the Covaris ultrasonicator S220 (settings: 10% duty factor, 200 cycles per burst and a peak incident power of 140). Ligated fragments were pulled down using streptavidin beads (Invitrogen) and prepared for Illumina sequencing by subsequent end-repair addition of A-overhangs and adapter ligation. Libraries were amplified for a total of 12 PCR cycles (45 s at 98 °C, 12 cycles of 15 s at 98 °C, 30 s at 55 °C, 30 s at 62 °C and a final extension of 5 min at 62 °C) and sequenced with the NOVASeq platform (Illumina), generating 100 bp paired-end sequence reads at the University of California San Diego core facility.

### Hi-C data processing

The Illumina sequencing of the four *B. duncani* clones (WA1, A6, B7 and B1) yielded over one billion Hi-C paired-end reads in total. Hi-C reads were processed using the command-line version of the HiCExplorer pipeline. HiCExplorer is a comprehensive and versatile pipeline that processes Hi-C reads and generates normalized chromatin conformation contact maps. The pipeline started by mapping all Hi-C single reads to the *B. duncani* assembly using ‘BWA mem -A1 -B4 -E50 -L0’^51^. The percentage of reads mapped ranged between 83% and 96%. After sorting and merging the reads from the four data sets, the command ‘hicBuildMatrix’ was used with default parameters and a bin size of 10 Kb. A diagnostic plot created using ‘hicCorrectMatrix diagnostic_plot’ indicated that a threshold of −4.5 would be appropriate to remove GC and open chromatin biases. The correction step, carried out by the command ‘hicCorrectMatrix correct’, also normalized the number of restriction sites per bin. The contact maps were plotted using ‘hicPlotMatrix’ as part of the same pipeline.

### 3D modelling

The 3D model of the *B. duncani* genome was generated and visualized using PASTIS^52^ and ChimeraX^53^. Briefly, interaction data were first manually fitted to a three-column interaction count matrix where the first two columns indicate the two separate bins within the genome and the third is the number of interactions between those two bins. The 3D coordinate matrices were then generated using PASTIS and converted to .PDB format, with each line being one of the coordinate outputs by PASTIS. The data were visualized using ChimeraX^53^. To investigate the spatial conformation of the *B. duncani* genome, a 3D model of the three chromosomes was first built using PASTIS^52^ and then improved using the Bezier curve smoothing method. PASTIS infers a consensus 3D structure from the genome-wide Hi-C contact frequency matrix using the following probability model. The programme models the observed chromatin contact frequencies as independent Poisson random variables and infers the model parameters as well as the 3D coordinates of the genome structure via the maximum-likelihood estimation approach. Specifically, PASTIS assumes the Poisson parameter *λ*_*ij*_ for chromatin interaction frequency *c*_*ij*_ between loci i and j as a decreasing function of *d*_*ij*_,

1 $${\lambda}_{ij} = \beta \,d_{ij}\left(X\right)^\alpha$$

with parameters *β* > 0 and *α* < 0. Here, *d*_*ij*_ is the Euclidean distance between loci *i* and *j* in structure *X*^52^. Therefore, the likelihood of observing *c*_*ij*_s can be formulated as:

2 $$L\left({X,\alpha ,\beta}\right) = \mathop {\prod}\limits_{\_}{(i,j)} {\mathrm{Poisson}}\left({\lambda _{ij}}\right) = \mathop {\prod}\limits_{\_ }{(i,j)} \frac{{\left({\beta d_{ij}^\alpha}\right)^{c_{ij}}}}{{c_{ij}!}}e^{\left({- \beta d_{ij}^\alpha}\right)}$$

By maximizing the log-likelihood function, PASTIS optimizes the 3D structure *X* as well as the distance decay parameters *α* and *β*. Since the 3D model predicted by PASTIS contained a set of discrete points, cubic Bezier curve smoothing was applied for the purpose of visualization. Cubic Bezier curves guarantee that the interpolated curve passes through all points in the original structure and the first-order geometric continuity at all points is maintained.

### PacBio IsoSeq processing

PacBio IsoSeq reads were mapped to the *B. duncani* genome using Minimap2^47^ with options ‘splice:hq -uf –secondary=no -C5’. The resulting SAM files were sorted using ‘sort -k 3,3 -k 4,4n’, then fed into the PacBio cDNA_Cupcake ToFU pipeline (https://github.com/Magdoll/cDNA_Cupcake) using the Python code ‘collapse_isoforms_by_sam.py’ to collapse redundant isoforms (more details about this pipeline can be found at https://github.com/Magdoll/cDNA_Cupcake/wiki). The resulting isoform sequences were used in the gene finding pipeline below.

### Gene prediction and annotation

The genome was first soft masked with RepeatMasker (http://www.repeatmasker.org), then processed using the FunAnnotate (v1.8.9) gene annotation pipeline (https://github.com/nextgenusfs/funannotate). As transcript evidence, we provided the IsoSeq-based isoforms computed via the cDNA_cupcake pipeline (see PacBio IsoSeq processing). As protein sources, we provided the annotated protein sets of *B. bigemina*, *B. bovis*, *B. microti*, *P. falciparum*, *T. gondii*, *T. orientalis*, *T. parva* as well as all the UniProt/SwissProt protein models. FunAnnotate was run with default parameters and weights (augustus:2 hiq:4 transcripts:4 proteins:4). Functional annotations were obtained via InterProScan (v5.55-88) with default parameters^54^. The output of InterProScan was parsed by custom scripts for the downstream analyses.

### Gene orthology-based classification analysis

A maximum-likelihood gene orthology-based classification analysis was generated using 190 single-copy orthologous proteins from 19 species. Orthology was generated with Proteinortho (v6.0.33)^55^ Proteinortho: Detection of (Co-)Orthologs in large-scale analysis^55^ with the following parameters: -cpus=64 -ram=462144 -p=blastp+-singles. Proteins were concatenated for each species and a multiple alignment was generated using MAFFTv7.505 with the –maxiterate 1,000 parameter^56^. The resulting multiple alignment was used as input to generate a tree with Raxml (v8.2.12) with the following parameters: -p 070378 -T 48 -m PROTCATIJTTF -# 1000 -f a -x 070378. Finally, the tree was visualized and generated using iTOL (v6^57^). An upset plot was created using the UpSetR (v1.4.0) R package and a csv matrix created from the ProteinOrtho orthologous protein analysis^55^. Basic instruction to create the plot can be found at https://cran.r-project.org/web/packages/UpSetR/vignettes/basic.usage.html

### Synteny and gene localization plots

Synteny plots were obtained using mummer2circos (https://github.com/metagenlab/mummer2circos) with the promer algorithm, and Circos^58^. Gene localization plots were produced using custom Python scripts and the Biopython Bio.graphics library.

### Orthology detection and database searches

*B. duncani* genes were assigned to OrthoMCL (https://OrthoMCL.org) groups using the orthology assignment tool available through the VEuPathDB (https://VEuPathDB.org) Galaxy workspace^59,60^. Translated *B. duncani* proteins (4,170) in FASTA format were assigned to groups on the basis of the OG6r9 blast database using default settings. Output files generated by the OrthoMCL pipeline included a mapping file between *B. duncani* gene IDs and OrthoMCL v.6 group IDs (this file was used to query the OrthoMCL database to determine degrees of evolutionary conservation) and a file of leftover proteins that did not map to any OrthoMCL groups but did cluster with at least one other *B. duncani* protein (these were considered species-specific gene duplications or families). To identify genes that do not have any orthologues or paralogues, the original input FASTA file was parsed for *B. duncani* IDs that are not present in any of the OrthoMCL mapping output files. VEuPathDB resources including PlasmoDB.org, ToxoDB.org, CryptoDB.org and PiroplasmaDB.org were used to retrieve genome size, gene content and chromosome numbers.

A matrix containing the number of genes per OrthoMCL groups was made from the annotation of *B. duncani* and species present in PiroplasmaDB. Annotations were compared by selecting OrthoMCL groups presenting annotation in at least 4 species. *B. microti* drastically reduced the number of OrthoMCL IDs and was removed from the analysis. OrthoMCL IDs were compared using Euclidian distance and hierarchical classification was performed using the Ward method. Species annotations were compared using presence/absence of the OrthoMCL ID. Jaccard distance and Ward methods were used to make the tree. All analyses were performed in R. The ComplexHeatmap function was used to generate the heat map.

### Probe generation for DNA FISH

The following PCR primers were designed for the amplification of ~1 kb region from the genomic loci of interest: Probe 1_For (Chr I; UMGF1): ACTCAGAATGTTAATGGAACGTC, Probe 1_Rev (Chr I; UMGF1): AATATCACTTTGAATCTTGCTAAC; Probe 2_For (Chr IV, UMGF1): TCAATCGTTCAATTCATCATCG, Probe 2_Rev (Chr IV, UMGF1): TCAATAACTTGCTGCAAATCAC; Probe 3_For (Chr III, UMGF3): GAAACTAGCGTAATCCTGTG and Probe 3_Rev (Chr III, UMGF3): AATAATCAGAAATGGTGGGTTCG. Fluorescein-labelled Probes 1 and 2 were generated by PCR using *B. duncani* WA1 genomic DNA as template and HighFidelity Fluorescein PCR labelling kit (APP-101-FAMX-L, Jena Biosciences). Biotin-labelled Probe 3 was generated by PCR using *B. duncani* WA1 genomic DNA as template and Biotin-16-dUTP kit (11093070910, Roche).

### DNA FISH

*B. duncani* WA1 parasites were cultured in vitro in 20 ml volume and maintained until 20% parasitemia was reached. The culture was treated with 0.015% saponin for 20 min in cold PBS, followed by centrifugation at 4,200 × *g* for 10 min. Following this, the parasite pellet was washed 7 times with 1X PBS. After the final wash, the cells were resuspended in 4% paraformaldehyde in PBS at room temperature and incubated on ice for 15 min. The parasite pellet was washed twice in 10 ml ice-cold PBS and resuspended in 0.25 ml ice-cold PBS. A monolayer of parasites (roughly 1 × 10^7^ parasites) was deposited on a 9 × 9 mm frame-seal slide chamber (AB-0576, Thermo Fisher) on a standard microscopy slide and allowed to dry for 1–2 h at r.t. Following this, the slides were washed with 50 μl PBS for 5 min at r.t. The parasites were then permeabilized in 50 μl 0.1% Triton X-100 (A16046.AP, Thermo Fisher) in PBS for 5 min at r.t. and then washed with 50 μl PBS for 5 min at r.t. The slides were equilibrated with 50 μl of hybridization solution (50% formamide (F5903, Teknova), 10% dextran sulfate (D6001, Sigma), 2x sodium saline phosphate EDTA (15591-043, Invitrogen), 250 μg ml^−1^ single-stranded DNA from salmon testes (D7656, Sigma)) for 30 min at 37 °C. In parallel, the PCR amplified DNA FISH probes (fluorescein-labelled probe 1, fluorescein-labelled probe 3 and biotin-labelled probe 2) were diluted to 1:20 in the hybridization solution, denatured at 95 °C for 5 min and immediately placed on ice for 5 min. The probes were then applied onto the slides; the slides were sealed with plastic frames (AB-0576, Thermo Fisher) and incubated at 80 °C for 30 min and then at 37 °C overnight. Next morning, the coverslip was removed, and the hybridization solution was discarded. The frame was also stripped out and the slides were washed in 15 ml 50% formamide/2xSSC (F5903, Teknova and 15557044, Invitrogen) buffer for 30 min at 37 °C. The slides were then washed sequentially in 15 ml each of 1xSSC, 2xSSC and 4xSSC at 50 °C for 15 min, and then equilibrated in M solution (100 mM maleic acid (M0375, Sigma-Aldrich), 150 mM NaCl (AB01915-01000, AmericanBio) and 1% bovine serum albumin (A9647, Sigma-Aldrich)) for 5 min at r.t. in a humid chamber protected from light. M solution was replaced with a new M solution containing avidin-rhodamine (1:5,000; A-6378, Thermo Fisher) and slides were incubated for 30 min at r.t. in a humid chamber protected from light. Following this step, the slides were washed 3 times in 30 ml TNT solution (100 mM Tris-HCl, pH 7.4 (AB14044-01000, AmericanBio), 150 mM NaCl (AB13198-01000, AmericanBio) and 0.5% Tween 20 (9005-64-5, Sigma-Aldrich)) at 10 min each at r.t. The slides were then mounted with coverslips using Vectashield mounting solution containing DAPI (H-1200-10, Vector Laboratories), and coverslips were sealed using nail polish. The slides were observed using a Nikon ECLIPSE TE2000-E microscope. A ×100 oil immersion objective was used for image acquisition. Excitation at 465–495 nm was used to detect fluorescein. Excitation at 510–560 nm was used to detect rhodamine, and excitation at 340–380 nm was used to detect DAPI positive cells. The images were acquired using Meta Vue with image size of 1,392 × 1,040 pixels and subsequently analysed using ImageJ.

### RNA-seq processing for gene-expression analysis

RNA-seq FASTQ files were assessed for quality using FastQC (v0.11.8). Adapter sequences as well as the first 11 bp of each read were trimmed using Trimmomatic (v0.39). Tails of reads were trimmed using Sickle with a Phred base quality threshold of 25, and reads shorter than 18 bp were removed. Reads were then aligned to the *B. duncani* genome assembly using HISAT2 (v2.2.1). Only properly paired reads with a mapping quality score of 40 or higher were retained, with filtering done using Samtools (v1.11). StringTie (v2.2.1) was run with the -e parameter to estimate the abundance of each gene in TPM (transcripts per million).

### ChIP-seq data analysis

Quality of the reads was analysed by FastQC (https://www.bioinformatics.babraham.ac.uk/projects/fastqc/). Sequence adaptors were removed using Trimmomatic v0.39 (http://www.usadellab.org/cms/?page=trimmomatic). Bases with Phred quality scores below 20 were trimmed using Sickle v1.330 (https://github.com/najoshi/sickle). Reads were mapped against the *B. duncani* assembly using Bowtie2 (v2.4.4) using default parameters, and PCR duplicates were removed by PicardTools MarkDuplicates v2.18.0 (Broad Institute). Only properly paired reads were retained using Samtools v1.11 (http://samtools.sourceforge.net/). To obtain the read coverage per nucleotide and generate genome browser tracks, we used BedTools (v2.27.1) and custom scripts to normalize the counts by millions of mapped reads. Chromosome tracks were viewed using IGV (Broad Institute). To compare H3K9me3 levels between MGF genes and other genes, we used bedtools multicov to calculate H3K9me3 (and IgG control) read counts within each gene body. These counts were then normalized to millions of mapped reads for each library and gene length in kb. Background signal from the IgG control was subtracted from H3K9me3 counts, with negative values set to 0. Similarly, H3K9ac read counts were also determined by bedtools multicov, but 300 bp on the 5’ side were included in addition to the gene body since histone H3K9 acetylation often occurs in promoter regions. Pearson correlations between ChIP samples were determined by taking genome-wide per-nucleotide read counts for each sample, normalizing by millions of mapped reads using R. To compare histone modifications with gene expression profiles, we generated a heat map using H3K9me3 and H3K9ac read counts, as well as the RNA-seq TPM levels for each gene. Read counts for H3K9me3 and H3K9ac were normalized to millions of mapped reads and gene length, while TPM was determined by Stringtie (see RNA-seq methods). Log scaling of all counts was used for the heat map, and genes were sorted from high to low TPM.

A separate Excel spreadsheet of ChIP-seq data is provided in Supplementary Information (Supplementary Data file 2).

### Evaluation of drug efficacy in vitro

Drug efficacy against *B. duncani* in vitro was determined using the SYBR Green assay as previously described^7^. Briefly, a culture of *B. duncani*-infected human erythrocytes at 0.1% parasitemia and 5% haematocrit was maintained for 60 h in vitro in a 96-well plate in the absence or presence of varying concentrations of the drug of interest at concentrations ranging between 0.6 nM and 10 µM. An equal volume of lysis buffer (0.008% saponin, 0.08% Triton X-100, 20 mM Tris-HCl (pH 7.5) and 5 mM EDTA) containing SYBR Green-I (0.01%) was added to each well and the plate incubated at 37 °C for 1 h in the dark. Fluorescence counts in each well were measured at 480 nm (excitation) and 540 nm (emission) using a BioTek Synergy Mx microplate reader and background fluorescence from control wells containing uninfected RBCs was subtracted. A dose-response curve representing the fluorescence counts against the logarithm of the drug concentration was fitted by nonlinear regression and used to determine the IC_50_ of each drug. Analysis was conducted using GraphPad Prism 9.2.1. For each drug, two independent experiments, each with biological triplicates, were conducted. Data are shown as mean ± s.d.

### Assessment of drug cytotoxicity on human cell lines

HeLa, HepG2, HEK and HCT116 cell lines were obtained from the American Type Culture Collection and maintained in Dulbecco’s modified Eagle’s medium (Invitrogen 11995-065) containing 25 mM glucose, 1 mM sodium pyruvate and supplemented with 5 mM HEPES, 10% FBS and penicillin-streptomycin (50 U ml^−1^ penicillin, 50 µg ml^−1^ streptomycin). An estimated 20,000 cells per well were seeded in a 96-well tissue culture plate and allowed to adhere. After 24 h, cells were treated with either vehicle alone (0.1% dimethylsulfoxide) or vehicle containing different concentrations of the target drugs using a 2-fold serial dilution starting at 10 mM. Dimethylsulfoxide at 10% was used as a positive control. Cells were incubated at 37 °C for 72 h, after which each well was incubated with 0.5 mg ml^−1^ of MTT reagent (Cayman Chemical, 10009591) for 4 h in the dark at 37 °C. Following addition of 100 µl dimethylsulfoxide to each well, absorbance was measured at optical density (OD)_590 nm_ using Spectra max plate reader. From the obtained OD values, percent cell viability was calculated by normalizing to the mean of 10% dimethylsulfoxide wells (set as 100% toxicity) and the mean of the vehicle control wells (set as 0% toxicity). Dose-response curves were plotted using GraphPad Prism v9.1.2

### Steady-state kinetics of DHFR-TS activity

DHFR kinetic experiments were performed by incubating purified enzyme (0.1 μM) with a saturating concentration of DHFR ligand (NADPH or dihydrofolate (DHF) at 300 μM) and measuring the reaction rate at varying concentrations of the complementary ligand (DHF or NADPH, respectively). The experiments were performed in a 96-well plate in a reaction buffer consisting of 25 mM Tris pH 8, 20 mM NaCl, 50 mM l-arginine, 0.5% glycerol, 1 mM dithiothreitol and 1 mM EDTA. Enzyme activity was determined by measuring the decrease in absorbance at 340 nm as NADPH is converted to NADP+. The plates were incubated at 37 °C, and OD_340_ measurements were taken using a BioTek SynergyMx microplate reader for 1 min. Rate constants for steady-state kinetic experiments were estimated by fitting the data to a Michaelis−Menten hyperbolic curve ((*v* = *V*_max_[*S*]/(*K*_*m*_ + [S])), where *v* is the reaction rate, [*S*] the concentration of the substrate, and *K*_m_ is the Michaelis constant) using the curve-fitting programme in GraphPad Prism.

### The IC_50_ of DHFR enzymatic activity

The inhibition of selected compounds on DHFR activity was measured by incubating purified enzyme (0.1 μM) with rising concentrations of the drugs. The reaction buffer contained 300 μM DHF and 300 μM NADPH, and the OD_340_ reduction rate (OD_340_ min^−1^) was documented for further calculation. The % inhibition was calculated and normalized to dimethylsulfoxide (100% activity) and no enzyme (0% activity) wells accordingly: 1 − (((OD_340_ min^−1^)_sample_ − (OD_340_ min^−1^)_neg. control_)/((OD_340_ min^−1^)_pos. control_ − (OD_340_ min^−1^)_neg. control_)). The IC_50_ was determined from a sigmoidal dose-response curve using GraphPad Prism v9.1.2.

Additional methods used in this study are detailed in Supplementary Information.

### Reporting summary

Further information on research design is available in the Nature Portfolio Reporting Summary linked to this article.

### Supplementary information

Supplementary Information Supplementary Figs. 1–7, Tables 1–3 and methods.

Reporting Summary

Peer Review File

Supplementary Data Chromatin IP readcounts H3K9me3. *B. duncani* gene annotation. BdUMGFs with RNA expression levels.

### Source data

Source Data Fig. 1 Unprocessed PFGE and Southern-blot images.

## Figures and Tables

**Extended Data Table 1:**
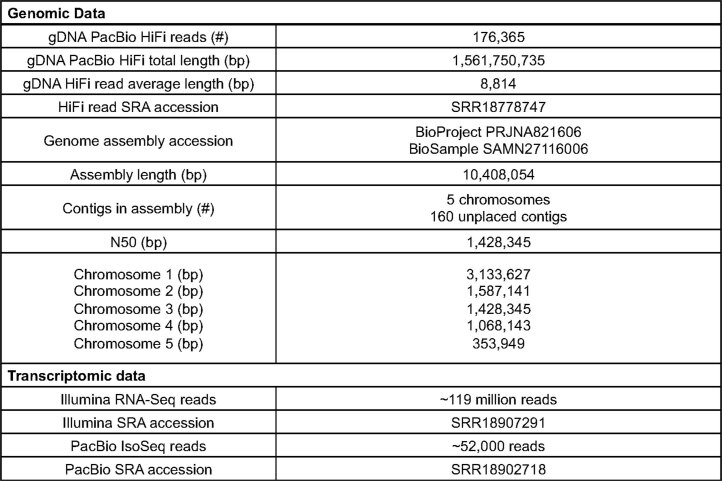
Genome & transcriptome data

**Extended Data Table 2:**
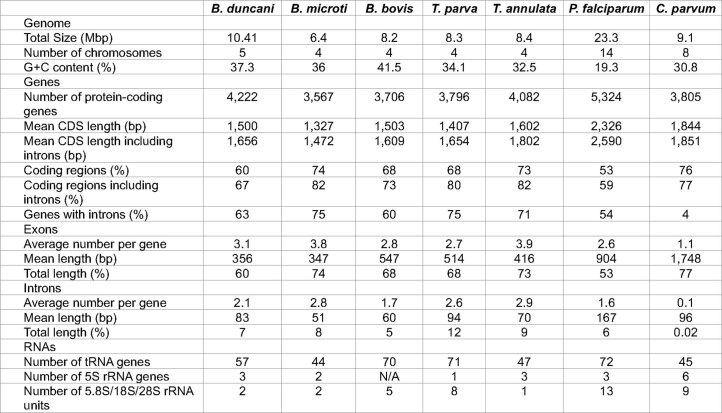
Genome comparison and gene statistics

**Extended Data Table 3:**
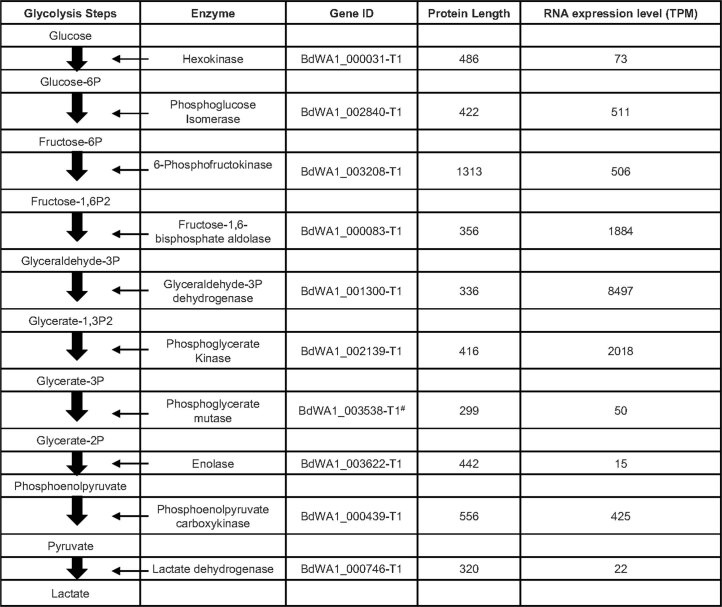
Glycolytic pathway in *B. duncani*

**Extended Data Table 4:**
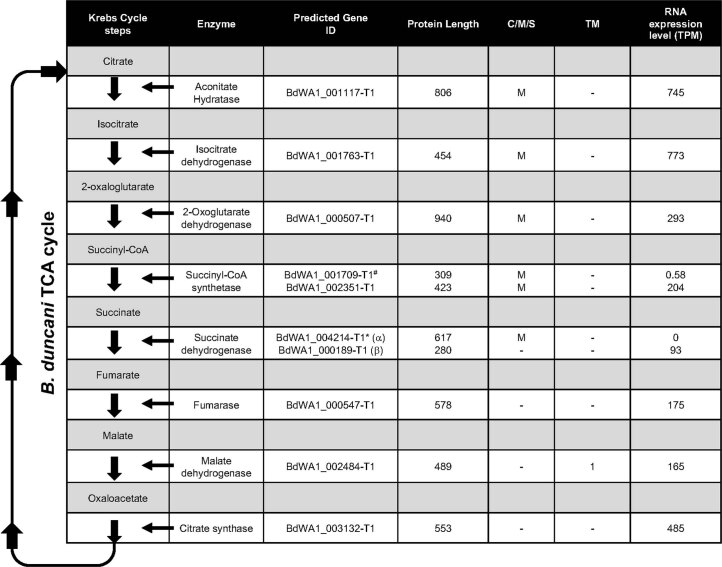
TCA cycle of *B. duncani*

**Extended Data Table 5:**
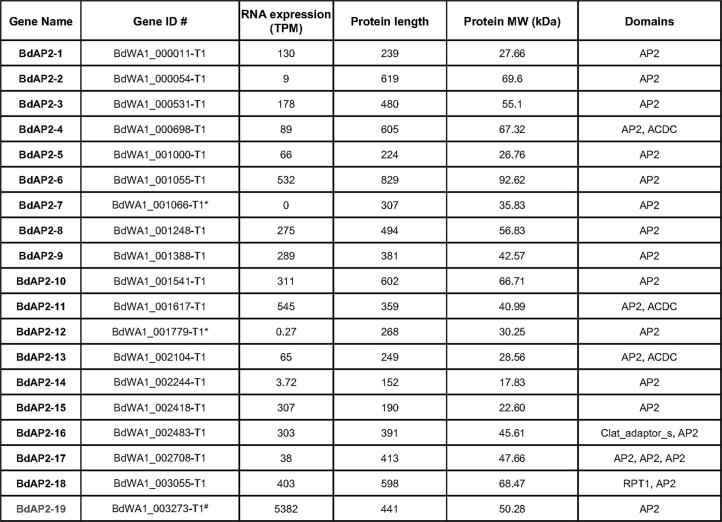
Predicted AP2 factors of *B. duncani*

**Extended Data Table 6:**
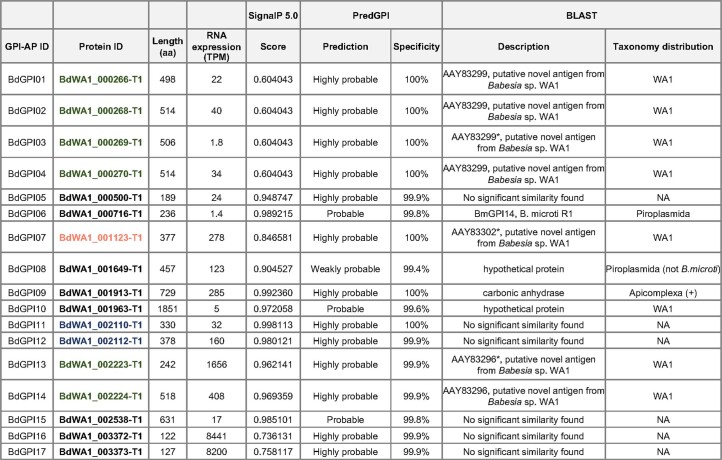
Predicted GPI-anchored proteins of *B. duncani*

**Extended Data Table 7:**
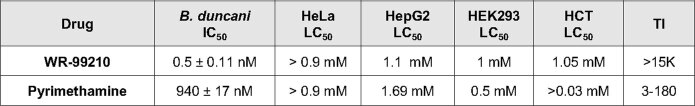
Therapeutic indexes of antifolates against different human cell lines cultured and tested in vitro

**Extended Data Table 8:**
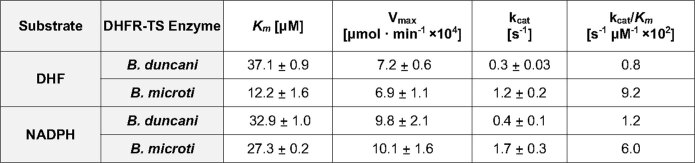
Kinetic parameters of purified recombinant DHFR-TS enzyme

## Data Availability

All the datasets generated for the current study are available in the NCBI/SRA repository, as Bioproject PRJNA821606, as follows: PacBio HiFi reads (SRA accession number SRR18778747), *B. duncani* genome assembly (NCBI Genome submission: SUB11253661), RNA-seq (SRA accession number SRR18907291), RNA Isoseq (SRA accession number SRR18902718), Hi-C reads (SRA accession number SRR19325692). Source data are provided with this paper.
