# Supplementary material for: Babesia duncani multi-omics identifies virulence factors and drug targets
Source: Nat Microbiol. 2023 Apr 13;8(5):845–59. doi: 10.1038/s41564-023-01360-8 (PMC10159843; doi:10.1038/s41564-023-01360-8)
Supplement: Supplementary file 1 — Supplementary Figs. 1–7, Tables 1–3 and methods. [file 41564_2023_1360_MOESM1_ESM.pdf]

nature microbiology

Article https://doi.org/10.1038/s41564-023-01360-8

Babesia duncani multi-omics identifies virulence factors and drug targets

In the format provided by the authors and unedited

Figure S1

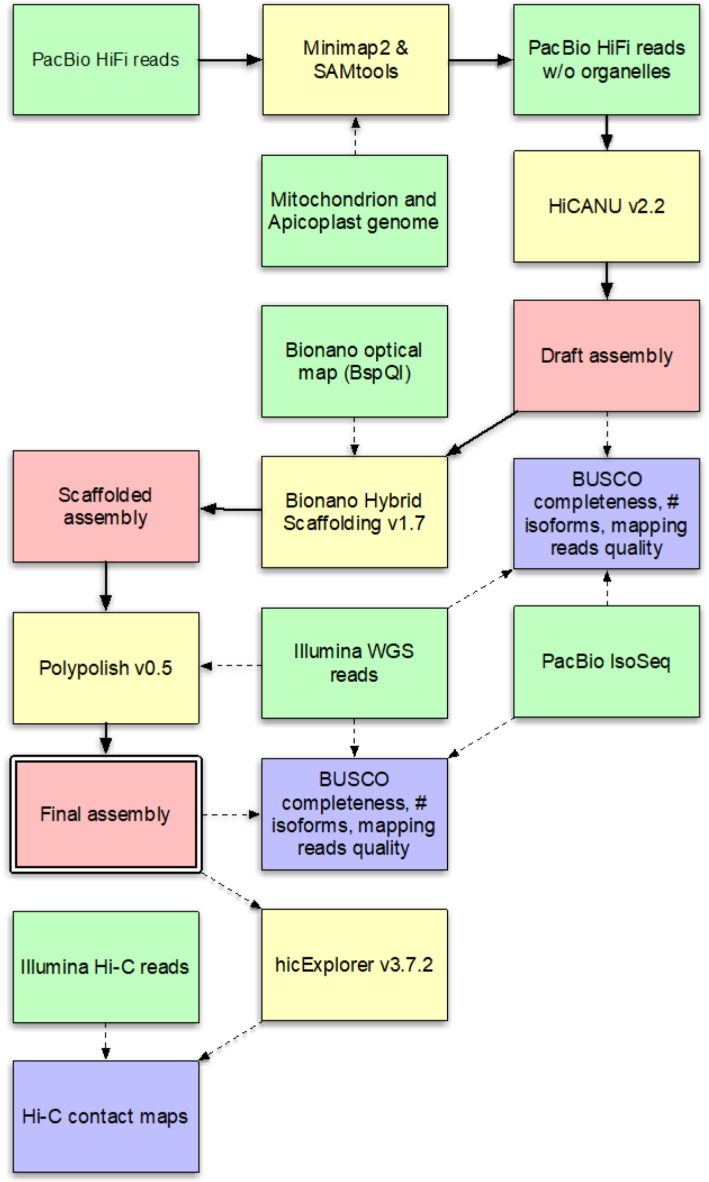

Figure S1: Software pipeline for the assembly of the *B. duncani* genome. Green blocks indicate genomics data sets; blue blocks indicate quality control terminal points; yellow blocks indicate software tools

Figure S2

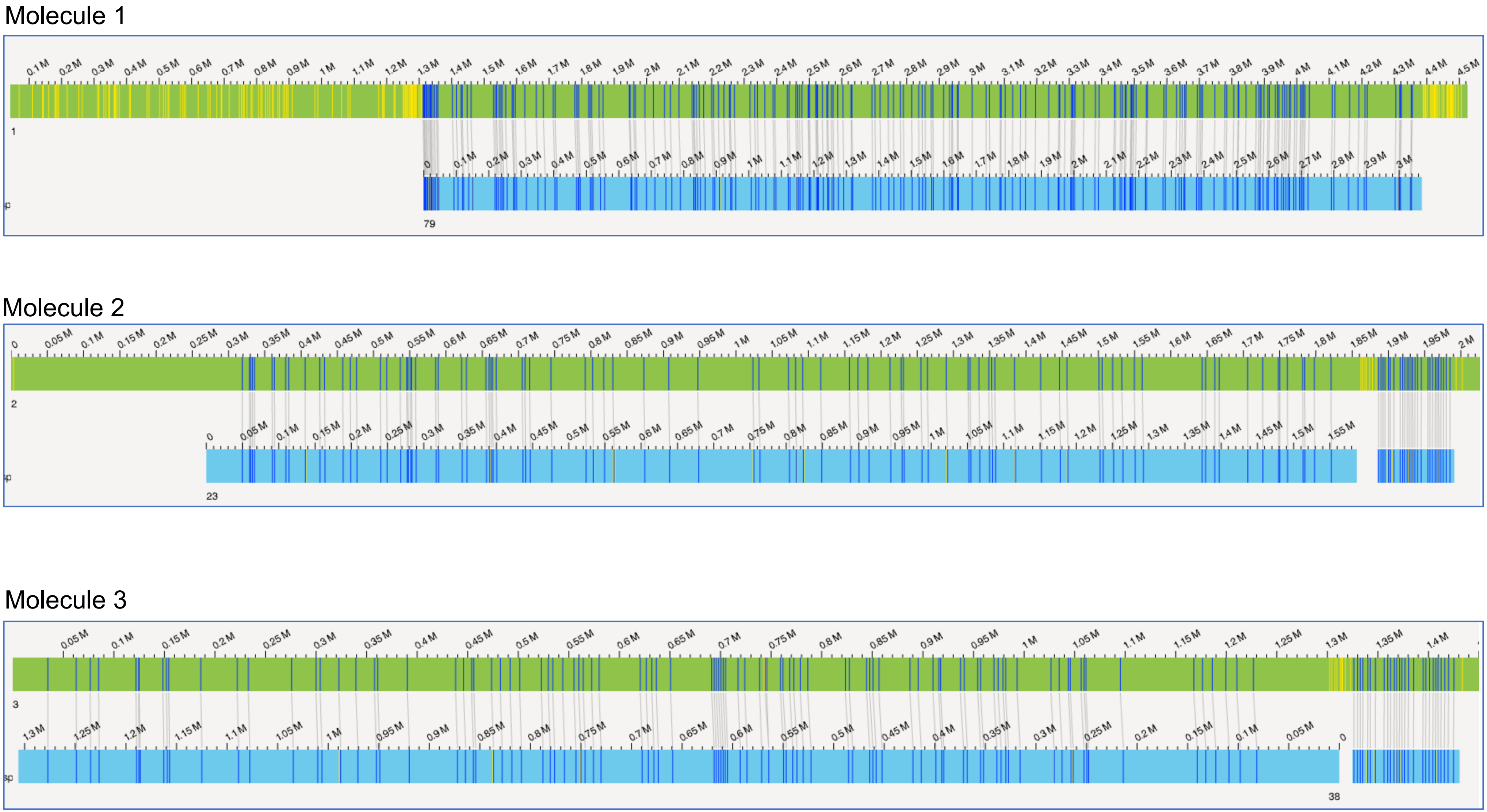

Figure S2: Optical Map (1/2). Alignment of the *Babesia duncani* genome assembly against molecule 1, 2 and 3 of the *B. duncani* optical map (BspQI).

Figure S2 continue

Molecule 4

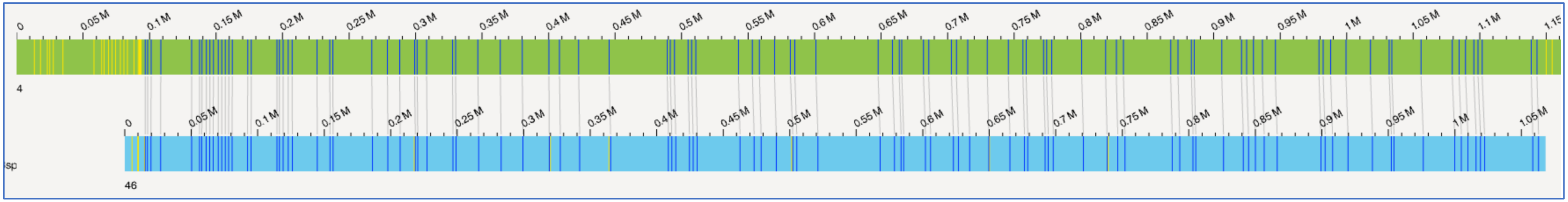

Molecule 5

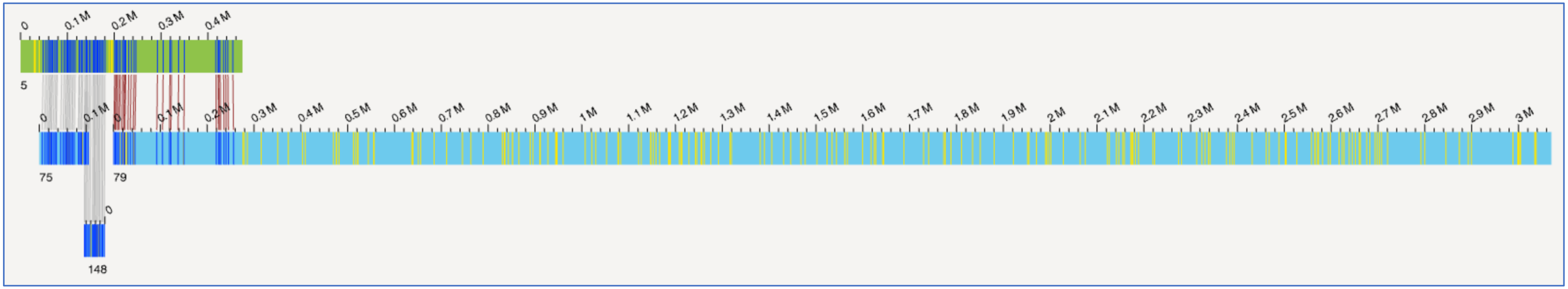

Molecule 6

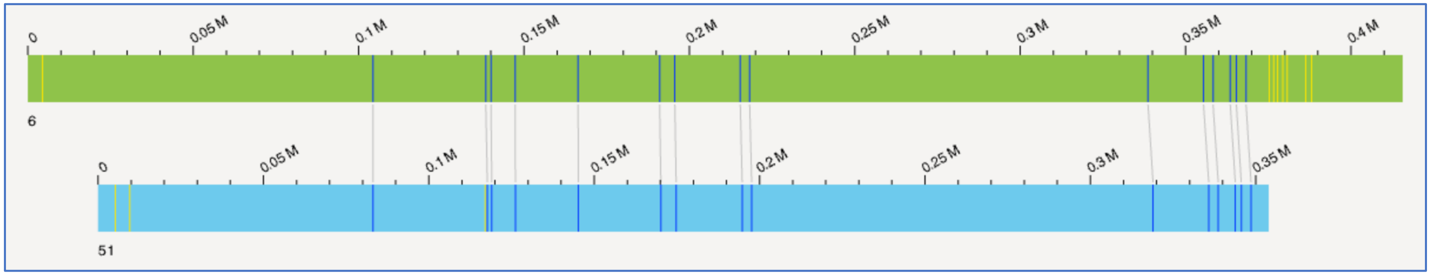

Figure S2 continue: Optical Map (2/2). Alignment of the *Babesia duncani* genome assembly against molecule 4, 5 and 6 of the *B. duncani* optical map (BspQI).

Figure S3

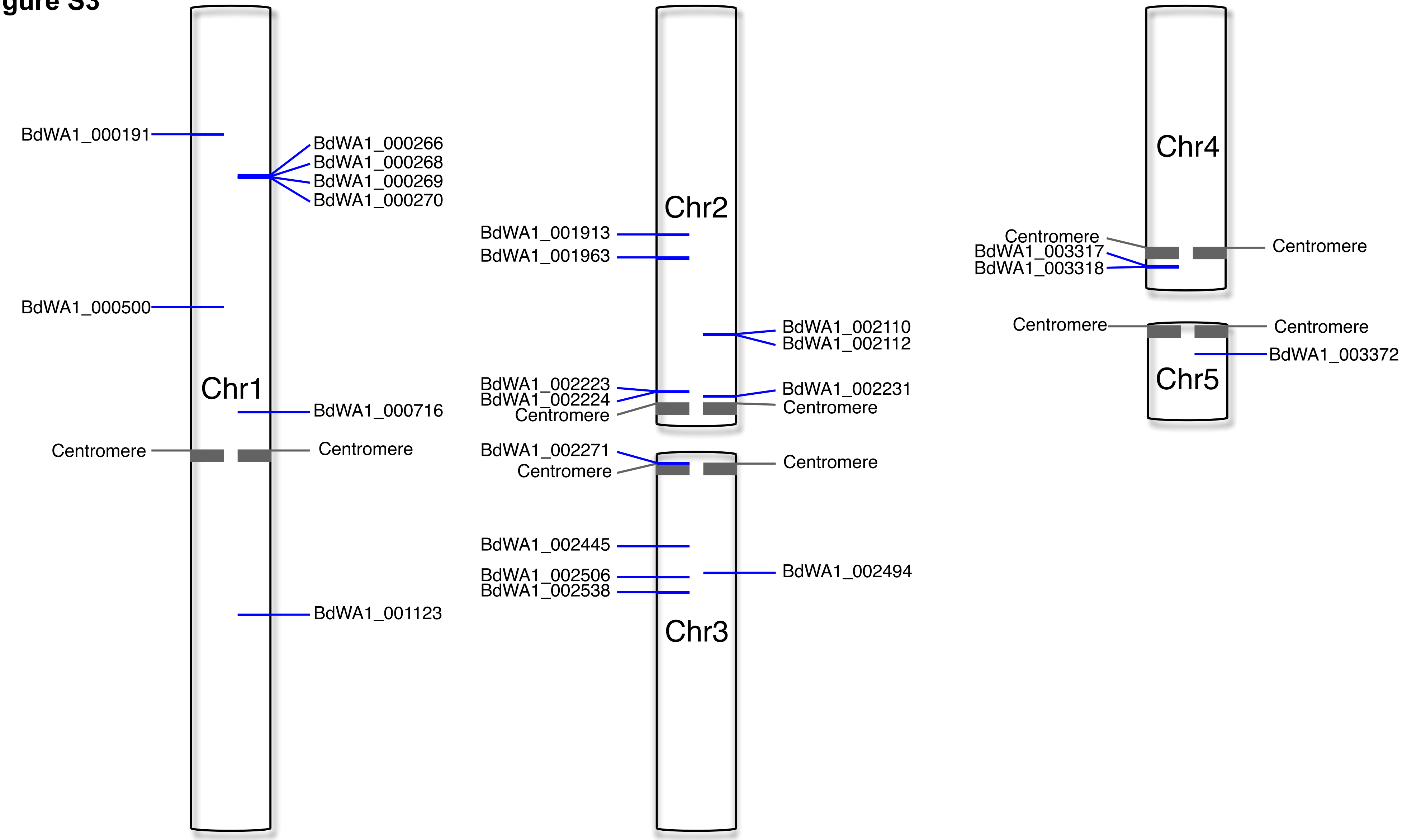

Figure S3: Localization of the genes in the gene families GPI-anchored proteins on the five *B. duncani* chromosomes (genes localized on unplaced contigs are ignored). genes on the right side of a chromosome are on the positive strand, genes on the left side are on the negative strand.

Figure S4

		Domains	Protein Length	TPM
**BdAP2-1**	BdWA1_000011-T1	AP2	239 aa	130
**BdAP2-2**	BdWA1_000054-T1	AP2	619 aa	9
**BdAP2-3**	BdWA1_000531-T1	AP2	480 aa	178
**BdAP2-4**	BdWA1_000698-T1	AP2	605 aa	89
**BdAP2-5**	BdWA1_001000-T1	AP2	224 aa	66
**BdAP2-6**	BdWA1_001055-T1	AP2	829 aa	532
**BdAP2-7**	BdWA1_001066-T1	AP2	307 aa	0
**BdAP2-8**	BdWA1_001248-T1	AP2	494 aa	275
**BdAP2-9**	BdWA1_001388-T1	AP2	381 aa	289
**BdAP2-10**	BdWA1_001541-T1	AP2	602 aa	311
**BdAP2-11**	BdWA1_001617-T1	AP2, ACDC	359 aa	545
**BdAP2-12**	BdWA1_001779-T1	AP2	268 aa	0.27
**BdAP2-13**	BdWA1_002104-T1	AP2, ACDC	249 aa	65
**BdAP2-14**	BdWA1_002244-T1	AP2	152 aa	3.72
**BdAP2-15**	BdWA1_002418-T1	AP2	190 aa	307
**BdAP2-16**	BdWA1_002483-T1	Clat_adaptor_s, AP2	391 aa	303
**BdAP2-17**	BdWA1_002708-T1	AP2, AP2, AP2	413 aa	38
**BdAP2-18**	BdWA1_003055-T1	RPT1, AP2	598 aa	403
**BdAP2-19**	BdWA1_003273-T1	AP2	441 aa	5382

Figure S4

		Protein Length	TPM
**BdAP2-1**	BdWA1_000011-T1	239 aa	130
**BdAP2-2**	BdWA1_000054-T1	619 aa	9
**BdAP2-3**	BdWA1_000531-T1	480 aa	178
**BdAP2-4**	BdWA1_000698-T1	605 aa	89
**BdAP2-5**	BdWA1_001000-T1	224 aa	66
**BdAP2-6**	BdWA1_001055-T1	829 aa	532
**BdAP2-7**	BdWA1_001066-T1	307 aa	0
**BdAP2-8**	BdWA1_001248-T1	494 aa	275
**BdAP2-9**	BdWA1_001388-T1	381 aa	289
**BdAP2-10**	BdWA1_001541-T1	602 aa	311
**BdAP2-11**	BdWA1_001617-T1	359 aa	545
**BdAP2-12**	BdWA1_001779-T1	268 aa	0.27
**BdAP2-13**	BdWA1_002104-T1	249 aa	65
**BdAP2-14**	BdWA1_002244-T1	152 aa	3.72
**BdAP2-15**	BdWA1_002418-T1	190 aa	307
**BdAP2-16**	BdWA1_002483-T1	391 aa	303
**BdAP2-17**	BdWA1_002708-T1	413 aa	38
**BdAP2-18**	BdWA1_003055-T1	598 aa	403
**BdAP2-19**	BdWA1_003273-T1	441 aa	5382

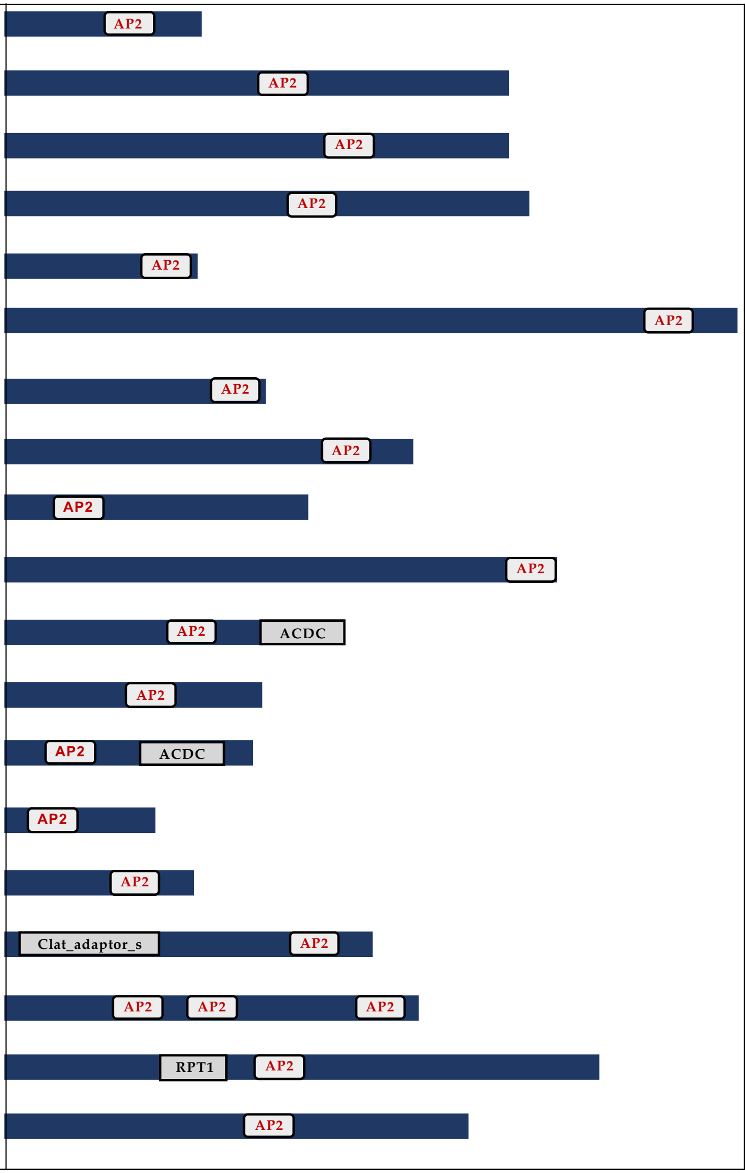

Figure S4: Schematic representation of AP2 protein family members in *B. duncani* WA1.

Figure S5

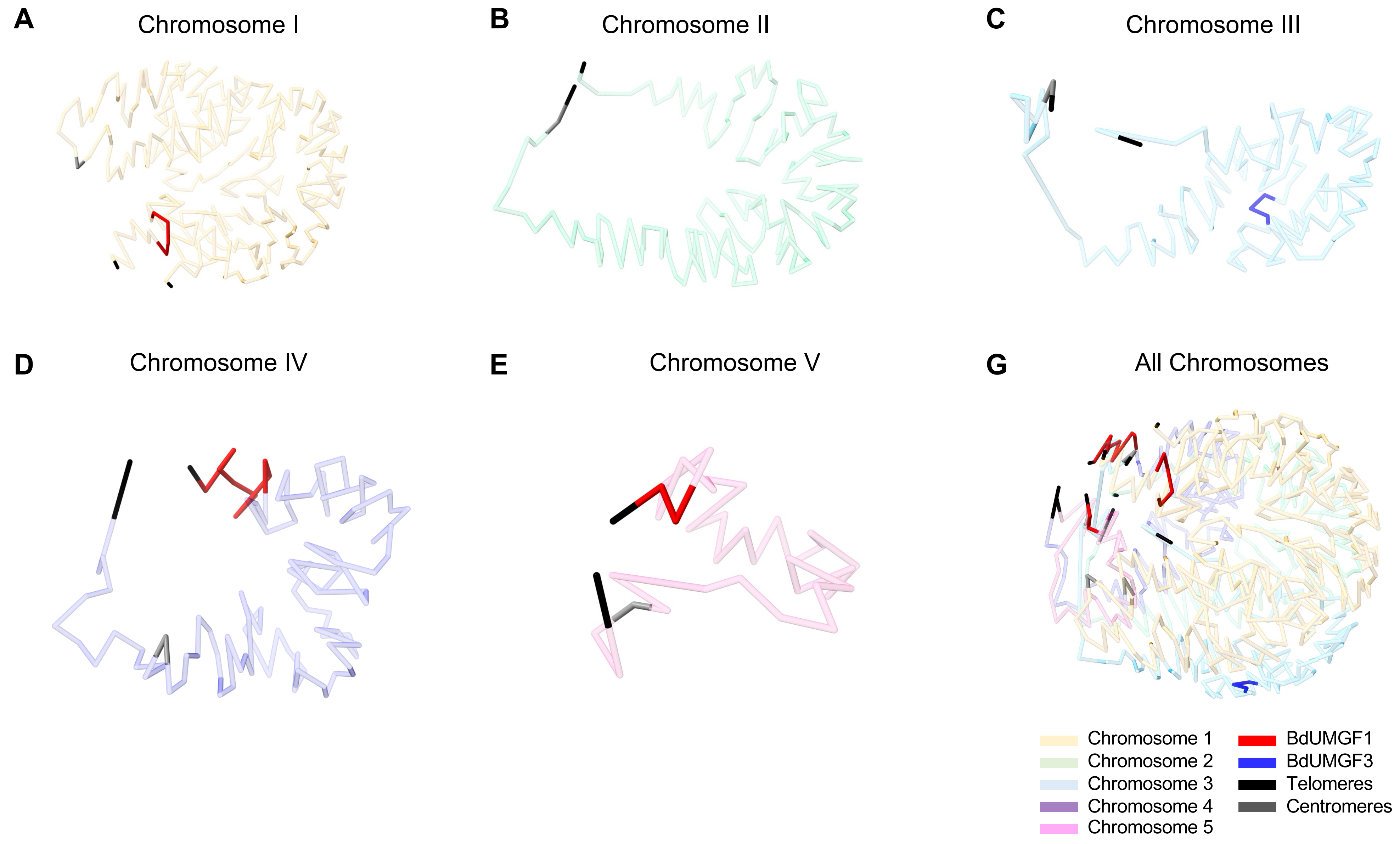

Figure S5: 3D-model representation of *B. duncani* chromosomes showing the location of different unique multigene families.

Figure S6

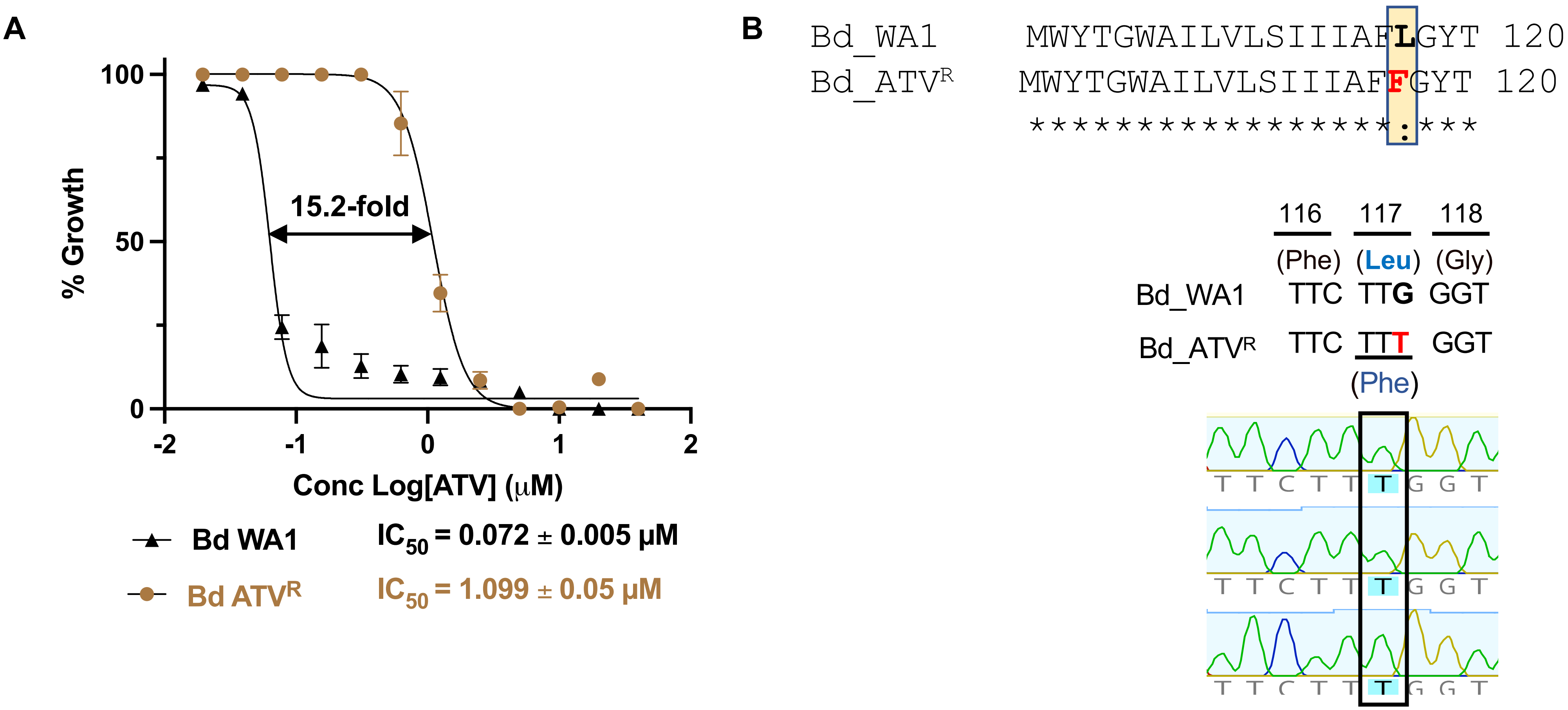

Figure S6: Atovaquone resistant *B. duncani* WA-1 parasites (BdATV^R) and the corresponding mutation in BdWA1 *Bdcytb* gene. **A.** Atovaquone dose-response curves for BdATV^R parasites and the corresponding parental line (BdWA1). **B.** a synonymous mutation in the *Bdcytb* gene that confers resistance to atovaquone. Data are shown as means ±SD of three independent experiments performed in biological triplicates.

Figure S7

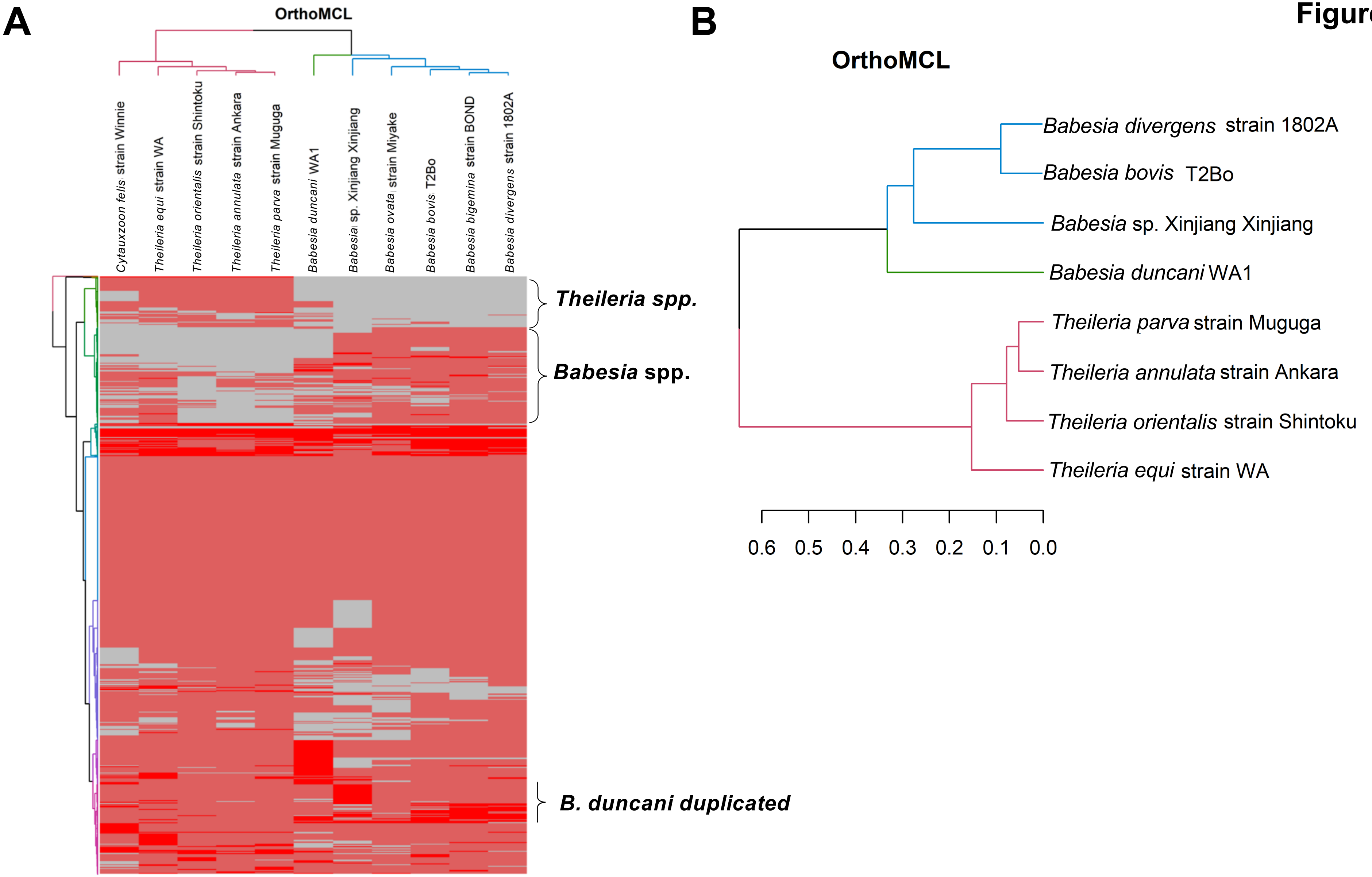

Figure S7: *B. duncani* OrthoMCL annotation and *Babesia* evolution. A. OrthoMCL annotation is summarized in a heatmap format. Groups were clustered based on the number of present genes. Grey color was used when no genes were present in a species. Light red means that only one gene is present and dark red correspond to OrthoMCL groups presenting more than 1 gene. Two clusters were specific of *Theileria* spp. on one side and *Babesia* spp. on the other side. The top tree is the same as in B. **B.** Evolution of piroplasmida based on OrthoMCL annotation. The Jaccard distance was used for binary analysis of the presence/absence of OrthoMCL groups in each species.

Table S1: Length of the six molecules in the *Babesia duncani* optical map (BspQI)

BspQI	Length (bp)
1	4,525,065
2	2,026,521
3	1,450,238
4	1,160,926
5	474,067
6	415,637
SUM	10,052,454

Table S2: Genome-wide read count Pearson correlations- ChIP-Seq analysis

**H3K9ac_rep1**	1							
**H3K9ac_rep2**	0.996	1						
**H3K9me3_rep1**	-0.22	-0.22	1					
**H3K9me3_rep2**	-0.222	-0.221	0.983	1				
**IgG_rep1**	0.196	0.195	0.042	0.039	1			
**IgG_rep2**	0.197	0.196	0.036	0.034	0.435	1		
**Input_rep1**	0.223	0.219	0.087	0.079	0.485	0.465	1	
**Input_rep2**	0.223	0.219	0.087	0.079	0.485	0.465	1	1
	**H3K9ac_rep1**	**H3K9ac_rep2**	**H3K9me3_rep1**	**H3K9me3_rep2**	**IgG_rep1**	**IgG_rep2**	**Input_rep1**	**Input_rep2**

Table S3: Emerging drug targets for human babesiosis therapy

Drug Target	*B. duncani* Protein ID	Potential Inhibitor(s)
Prolyl-tRNA synthetase	BdWA1_000148-T1	Febrifugine[C], Halofuginone[#]
Lysyl-tRNA synthetase	BdWA1_001431-T1	Cladosporin[C]
Isoleucyl-tRNA synthetase	BdWA1_003237-T1	Mupirocin[#]
Threonyl/alanyl tRNA synthetase	BdWA1_002127-T1	Borrelidin[#]
Tryptophanyl-tRNA synthetase	BdWA1_002202-T1	Indolmycin[C]
Translation EFG/EF2, Elongation factor EFG	BdWA1_002454-T1	DDD107498[C]
Phosphatidylinositol 4-kinase gamma	BdWA1_001807-T1*, BdWA1_004106-T1*	KDU691[C], MMV048[C], KAI407[C], BQR695[C], BRD73842[C]
Cytochrome b-c1 complex subunit 7 superfamily	BdWA1_002037-T1	Atovaquone*, Decoquinate*, Tetracyclic Benzothiazepine[C], GW844520[C], GSK932121[C]
P-type ATPase	BdWA1_002091-T1	KAE609 (Cipargamin[)C,] GNF-Pf-4492[C], PA21A092[C], SJ733[C]
Polyadenylation specificity factor subunit 2	BdWA1_003046-T1	AN3661[C]
Cytidyltransferase-like domain	BdWA1_003708-T1	Fosmidomycin[C], MMV008138[C]
Farnesyltransferase subunit beta	BdWA1_000911-T1	BMS-388891[C], MMV019066[C]
Dihydrofolate reductase-Thymidylate synthase	BdWA1_001462-T1	Pyrimethamine[#], Proguanil[#], MMV027634[C]

* = Identical proteins   # = Clinically approved drugs   c = Drugs under clinical evaluation for malaria

Supplemental Methods

Sequencing and Assembly using PacBio HiFi reads, Bionano Genomics optical map, Illumina WGS, and Illumina Hi-C. A total of 177,189 PacBio HiFi reads were obtained; the average HiFi read length was 8,814 bp; the longest read was 29,067 bp. The HiFi reads totaled 1.56B bases which translated to an expected ~156x coverage of the *B. duncani* genome (assuming a ~13Mb genome). HiFi reads were mapped using Minimap2 [17] to the *B. duncani* mitochondrion and apicoplast. Only 0.41% of the reads mapped to these organelles, which were then discarded to enrich the data for nuclear DNA. A Bionano optical map was also generated to correct and scaffold the assembly: the map generated using the restriction enzyme identified six molecules of sizes 4.5Mb, 2Mb, 1.4Mb, 1.1Mb, 474Kb and 451Kb, respectively, for a total of 10Mb (**Table S1 and Fig. S2**). PacBio HiFi reads were first filtered of mitochondrial and apicoplast genomes[18] to enrich for nuclear DNA sequences, then assembled with HiCANU [19]. The *de novo* draft assembly produced by HiCANU consisted of 167 contigs, including four contigs over 1Mb. The total assembly length was 10.37Mb with a N50 of ~1.3Mb, L50 of 3 contigs, and a NG50 of ~1Mbp (**Table 1**). The draft assembly was compared with the Bionano optical map to detect possible mis-joins and to create scaffolds (**Fig. S2**). The Bionano Hybrid Scaffolding pipeline detected no chimeric contigs and created four scaffolds, two of which were composed by two HiCANU contigs with a 16-19Kb gap in-between them. The rest of the assembled HiCANU contigs were not scaffolded and kept for the final assembly. The scaffolded assembly was polished using Polypolish [20] : two rounds of polishing using Illumina WGS reads only corrected less than 2K bases in the genome, which confirmed the very high-quality nucleotide level assemblies obtained from HiFi reads. The final polished assembly had five chromosome-level scaffolds of 3.13Mb (Chromosome I), 1.58Mb (Chromosome II), 1.42Mb (Chromosome III), 1.07Mb (Chromosome IV), and 0.35Mb

(Chromosome V) with a total of only 35Kb in two gaps (see **Table 1** for other statistics). The assembly pipeline is illustrated in **Fig. S1**. Additional details of the pipeline are provided in Material and Methods.

We also checked the assembly for mis-joints and assembly errors using the long-range chromatin contact frequency information provided by Hi-C analysis. We recently used this methodology to correct the genome sequences of *Plasmodium knowlesi* [21] and *Toxoplasma gondii* [22]. The contact map for *B. duncani* WA1 is shown on **Fig. 2A-2B**. A close examination of the contact maps indicate that the assembly has no large mis-joints or mis-assemblies and is consistent with other apicomplexan parasites [22]. The centromeres interact strongly with each other (as illustrated in the diagram below **Fig. 2B**). Chromosome I was found to have a metacentric centromere (i.e., the centromere is located at the center of the chromosome and the two arms are almost equal in length); Chromosomes II and III have a telocentric profile (i.e. the centromeres are present at the very end of the chromosomal arm); and Chromosomes IV and V have acrocentric centromeres (i.e. the centromeres are present closer to one end of the chromosomal arm with one arm shorter than the other). A similar organization was previously observed in three of the four chromosomes of *Babesia bovis* [23]. Using BUSCO v5 [24], a software tool that measures completeness and redundancy of a genome in terms of expected gene content, we found that the *B. duncani* WA1 nuclear genome has 95.1% of the gene models derived from the apicomplexa_odb10 database (85.2% single-copy, and 9.9% duplicated) with another 1.8% fragmented gene models, and only 3.1% of the gene models missing.

RNA preparation for Illumina RNA-Seq. Total RNA was isolated from parasites using 5 volumes of 37°C pre-warmed Trizol LS Reagent (Life Technologies, Carlsbad, CA, USA) and

incubated at 37°C for 5 minutes. Total RNA isolation was then continued according to the manufacturer's instructions. Total RNA was then treated with DNA-free DNA removal kit (ThermoFisher; AM1906) followed by mRNA purification using NEBNext Poly(A) mRNA Magnetic Isolation Module (NEB, E7490S). RNA-seq library was constructed using NEBNext Ultra II RNA-library preparation kit (NEB, E7770S) according to the manufacturer's instructions. The libraries were amplified for 15 PCR cycles (45s at 98°C followed by 15 cycles of [15s at 98°C, 30s at 55°C, 30s at 62°C], 5 min 62°C). Libraries were sequenced at 150 bp paired-end sequenced on the Illumina Novaseq platform (Illumina, San Diego, CA) at the UCSD and Yale core facility.

RNA preparation for PacBio Iso-Seq. TRIzol reagent (Life Technologies, Carlsbad, CA, USA, No. 15596–026) was used to isolate total RNA from 100 ml *in vitro* culture of *B. duncani* (15% parasitemia and 5% hematocrit) according to the manufacturer's protocol. 1 µg of total RNA was used for the synthesis and amplification of cDNA using a combination of NEBNext Single Cell/Low Input cDNA Synthesis & Amplification module (Cat. No. E6421S), NEBNext High-Fidelity 2X PCR Master Mix (Cat. No. M0541S), Iso-Seq Express Oligo Kit (Cat. No. PN 101-737-500), and elution buffer (Cat. No. PN 101-633-500). SMRTbell libraries were constructed according to the Iso-Seq Express Template Protocol (Pacific Biosciences). Primer annealing and polymerase binding were performed following the SMRT Link v8.0 Sample Setup instructions and 90 pM of the SMRTbell templates were loaded for sequencing. One SMRT Cell 8M was used for each sample and sequencing was performed using the Sequel II system.

ChIP-Sequencing. *Babesia duncani* WA1 samples (approximately 20 million parasites per sample) were crosslinked with formaldehyde and resuspended in 1 mL of a nuclear extraction buffer (10 mM HEPES, 10 mM KCl, 0.1 mM EDTA, 0.1 mM EGTA, 1 mM DTT, 0.5 mM

AEBSF, 1X Roche protease inhibitor tablet, and 1X Roche phosphatase inhibitor tablet) and incubated on ice for 30 minutes. Following the incubation, 10% Igepal CA-630 was added to each sample and homogenized by passing the sample through a 26G × ½ needle. The nuclear pellet, obtained through centrifugation at 5,000 rpm, was then resuspended in shearing buffer (0.1% SDS, 1 mM EDTA, 10 mM Tris–HCl (pH 7.5), 1X Roche protease inhibitor tablet, and 1X Roche phosphatase inhibitor tablet) and transferred into a 130 µl Covaris sonication tube. Each sample was subjected to mechanical shearing by a Covaris S220 Ultrasonicator for 6 minutes (duty cycle: 5%, intensity peak power: 140, cycles per burst: 200, bath temperature: 6°C). To each sample, equal volume ChIP dilution buffer (30 mM Tris–HCl (pH 8), 3 mM EDTA, 0.1% SDS, 30 mM NaCl, 1.8% Triton X-100, 1X protease inhibitor tablet, 1X phosphatase inhibitor tablet) was added before being subjected to centrifugation for 10 minutes at 13,000 rpm at 4°C and collecting the supernatant. For each sample, 13 µl of protein A agarose/salmon sperm DNA beads were washed 3 times with 500 µl with ChIP dilution buffer. Each sample was pre-cleared by adding the diluted chromatin to the 13 µl of beads which were incubated for 1 hour at 4°C with agitation. For each of the pre-cleared samples, approximately 10% by volume was kept as input when needed, after which the appropriate antibody (anti-H3K9me3 (Abcam ab8898), anti-H3K9ac (Diagenode C15410003), or IgG (Abcam ab46540)) was added with rotation overnight at 4°C. For each sample, 25 µl of protein A agarose/salmon sperm DNA beads were washed with unsupplemented ChIP dilution buffer, before being blocked with a 1 mg/ml BSA solution at 4°C for 1 hour. After blocking, the beads were washed 3 times, each time with unsupplemented ChIP dilution buffer. 25 ul of the washed and blocked beads was added to each sample for 1hr at 4°C with rotation. Samples were removed from beads, and the bead/antibody/protein complex then underwent a series of eight 15 minute 1 mL washes with rotation. The washes are as follows: twice with low-salt immune

complex wash buffer (1% SDS, 1% Triton X-100, 2 mM EDTA, 20 mM Tris–HCl (pH 8), 150 mM NaCl), twice with high-salt immune complex wash buffer (1% SDS, 1% Triton X-100, 2 mM EDTA, 20 mM Tris–HCl (pH 8), 500 mM NaCl), twice with LiCl wash buffer (0.25M LiCl, 1% NP-40, 1% Na-deoxycholate, 1mM EDTA, 10mM Tris-HCl (ph 8)) and twice with TE buffer. Then the antibody-protein complex was eluted from the beads with two 250 ul washes of freshly prepared elution buffer (1% SDS, 0.1 M sodium bicarbonate). To each sample, a 55ul 5M NaCl solution was added, and the samples were left in a 45°C bath to reverse the crosslinking overnight. Following this, 15 µl of 20 mg/mL RNAse A was added to each sample which then incubated in a 37°C bath for 30 minutes followed by a 2 hr incubation with 10 µl 0.5 M EDTA, 20 µl 1 M Tris–HCl (pH 7.5), and 2 µl 20 mg/mL proteinase K in a 45°C bath. DNA recovery was performed via phenol/chloroform extractions and ethanol precipitation. Two extractions were done with phenol/chloroform/isoamyl alcohol (25:24:1), followed by a final chloroform extraction. The samples then precipitated overnight at a temperature of -20°C. After precipitation, samples were subjected to a 13,000 rpm centrifugation for 30 minutes at 4°C, pelleting out the DNA. The pellet was washed with an 80% ethanol solution and pelleted out again with centrifugation at 13,000 rpm for 15 minutes. After the final ethanol wash, the pellet was air dried and resuspended in 50 ul of nuclease-free water. DNA was purified using Agencourt AMPure XP beads (Beckman Coulter, CA, USA). Libraries were then prepared from this DNA using a KAPA library preparation kit (KK8230 & KK8500) and sequenced on an Illumina NovaSeq 6000 machine.

Phylogenetic tree reconstruction. To determine the phylogenetic placement of *B. duncani* among a selected group of apicomplexa, a conserved helicase from OrthoMCL group OG6_100305 (PMID: 26738725) was used for the alignments. Proteins from the group that were most similar

to *B. duncani* gene BdWA1_002520 were selected and aligned using the MUSCLE (1). Evolutionary history was inferred using the Neighbor-Joining method (2). Bootstrapping analysis was conducted 1,000 times to determine phylogenetic tree reliability. Evolutionary analysis was conducted using MEGA X software (3).

Pulse field gel electrophoresis (PFGE). *B. duncani* WA1 cultures were centrifuged at 1300 x g for 5 min to yield pellets containing intact cells. Pellets, were embedded in 1% (w/v) SeaKem Gold Agarose (Lonza, Rockland, ME, USA) to an approximately concentration of $1x10^8$ infected RBCs/ml. The resultant agarose plugs were incubated in lysis solution (100mM EDTA, pH8.0, 0.2% sodium deoxycholate, 1% sodium lauryl sarcosine) supplemented with 1 mg/ml of proteinase K (Thermo Fisher Scientific, Vilnius, Lithuania) for 24 h at 50°C. Finally, plugs were washed 4 times for 30 min each in wash buffer (20 mM Tris, pH 8.0, 50 mM EDTA).

Intact chromosomes were separated on a 0.8% Megabase Agarose gel (Bio-Rad Labs Inc., Hercules, CA, USA) in 1X TAE buffer chilled at 14°C for 48 h on a CHEF Mapper™ XA pulsed field electrophoresis system (Bio-Rad). The switch time was 500 sec at 3V/cm with an include angle of 106º. The agarose gel was stained with GelRed (Biotium, Fremont, CA, USA) and visualized under ultraviolet.

Southern Blot Analysis. Telomeric ends of *B. duncani* WA1 chromosomes were analyzed by Southern blot using a nucleotide repeat sequence (CCCTGAACCCTAAA) of the telomeric ends of *Plasmodium berghei* chromosomes (4,5). The telomeric probe was labeled using the DIG Oligonucleotide Tailing Kit, 2nd Generation (Cat. No. 03353383910, Roche, Mannheim, Germany). After PFGE and before transfer, DNA from the gel was depurinated (20 min in 0.25 M HCL),

denatured (2 X 20 min in 0.5N NaOH; 1.5 M NaCl) and neutralized (2 X 20 min in 0.5 M Tris-HCl, pH 7.5; 1.5 M NaCl). Southern blotting was done on nylon membrane, positively charged (Cat. No. 1417240, Roche) using 10X SSC and followed by UV crosslinking of transferred DNA. The membrane was hybridized overnight at 26ºC with the telomere probe and then washed twice for 5 min each in 2X SSC and for 20 min each in 0.5X SSC at 26°C. Bound probe was detected with disodium-2-chloro-5(4 methoxyspiro (1,2-dioxetane-3.2′-[5-chloro]tricycle[3.3.1.1.$^{3.7}$] decan)-4-yl)-1-phenyl phosphate (CDP-StarTM, Cat. No.12041677001, Roche) according to the manufacturer's instructions. The membrane was visualized using an Amersham ImageQuant 800 system (GE Healthcare Bio-Science AB, Uppsala, Sweden).

Cloning, Expression, and Purification of recombinant *B. duncani* and *B. microti* DHFR-TS enzymes. Dihydrofolate reductase-thymidylate synthase (DHFR-TS) gene from *B. duncani* (*Bd*DHFR-TS) and *B. microti* (*Bm*DHFR-TS) were cloned into pMAL-c4x by GenScript USA Inc. Both enzymes were expressed as fusion proteins with N'-terminal maltose-binding protein (MBP) tag. Constructs of pMAL-c4x-*Bd*DHFR-TS and pMAL-c4x-*Bm*DHFR-TS were transformed by heat shock into *E. coli* BL-21(DE3). *E. coli* BL-21(DE3) harboring pMAL-c4x-DHFR-TS was grown overnight in 2 ml LB ampicillin (100 µg/mL). Cultures were diluted 100-fold in fresh LB medium with 0.2% glucose and grown to OD_{600nm} of ~0.5 at 37°C. DHFR-TS expression was induced by the addition of 0.5 mM isopropyl thiogalactoside (IPTG) followed by growth for overnight at 16°C or at room temperature (RT). The cells were harvested by centrifugation (8,000 × g × 10 min, 4°C), washed by resuspension in water, and re-centrifuged. Cells were used directly for purification or kept frozen at -20°C. Prior to enzyme purification, cells were re-suspended in binding buffer containing 25 mM Tris-HCl pH 8, 500 mM NaCl, 0.5%

glycerol, and 50 mM L-arginine. Cells were supplemented benzonase DNAse (250U/µl) and 3-((3-cholamidopropyl) dimethylammonium)-1-propane sulfonate (CHAPS) (0.002%) and were disrupted by sonication on ice using Omni Sonic Ruptor 400 Ultrasonic Homogenizer (15-sec burst at 70% amplitude, 3 times, with 30-sec cooling intervals). A soluble supernatant was prepared by centrifugation (16,000 × g × 20 min) of the cells sonicate.

Recombinant DHFR-TSs were purified from the clear cell extract supernatant using amylose resin (NEB), methotrexate-agarose beads, and in some cases, size exclusion chromatography (SEC). Briefly, the soluble supernatant was incubated with amylose beads in binding buffer with gentle agitation for 1h at 4°C. Following enzyme adsorption, the affinity matrix was transferred to a column, and unbound proteins were removed by washing with a binding buffer. Purified enzymes were eluted with elution buffer containing 10 mM maltose. Amylose elution fractions were loaded onto MTX-agarose resin, and purified DHFR-TS enzymes were eluted using 2 mM dihydrofolic acid (DHF). Maltose or DHF residuals were removed by dialysis or using a PD-10 column (GE Healthcare), and the Purified protein stocks were then adjusted to 25% glycerol v/v, aliquoted, flash-frozen, and stored at -80˚C.

GPI-associated proteins (GPI-AP) identification. GPI-AP were selected based on a two transmembrane domain model, SignalP 5.0 and PredGPI online predictions. TMPred software was used to validate the presence of hydrophobic regions associated with the N-terminal signal peptide (ER-targeting) and C-terminal GPI-anchor signal (recognized by the transamidase). The maximum distance between the N-terminal TM with the start of the sequence was set to 10 with a minimal score of 500. The maximum number of residues between the C-terminal TM with the end of the sequence was 3 with a minimal score of 780. Internal hydrophobic helix should not have score

higher than 800. The proteins that were positive for all the three predictions were kept. Proteins lower than 100 aa were not considered.

References

1. Edgar, R.C. (2004) MUSCLE: multiple sequence alignment with high accuracy and high throughput. *Nucleic Acids Res*, **32**, 1792-1797.
2. Saitou, N. and Nei, M. (1987) The neighbor-joining method: a new method for reconstructing phylogenetic trees. *Mol Biol Evol*, **4**, 406-425.
3. Kumar, S., Stecher, G., Li, M., Knyaz, C. and Tamura, K. (2018) MEGA X: Molecular Evolutionary Genetics Analysis across Computing Platforms. *Mol Biol Evol*, **35**, 1547-1549.
4. Depoix, D., Carcy, B., Jumas-Bilak, E., Pages, M., Precigout, E., Schetters, T.P., Ravel, C. and Gorenflot, A. (2002) Chromosome number, genome size and polymorphism of European and South African isolates of large Babesia parasites that infect dogs. *Parasitology*, **125**, 313-321.
5. Ponzi, M., Pace, T., Dore, E. and Frontali, C. (1985) Identification of a telomeric DNA sequence in Plasmodium berghei. *EMBO J*, **4**, 2991-2995.
