# Supplementary material for: Babesia duncani multi-omics identifies virulence factors and drug targets
Source: Nat Microbiol. 2023 Apr 13;8(5):845–59. doi: 10.1038/s41564-023-01360-8 (PMC10159843; doi:10.1038/s41564-023-01360-8)
Supplement: Supplementary file 2 — Reporting Summary [file 41564_2023_1360_MOESM2_ESM.pdf]

nature portfolio

Corresponding author(s): DBPR, NMICROBIOL-22051126B

Last updated by author(s): Feb 22, 2023

Reporting Summary

Nature Portfolio wishes to improve the reproducibility of the work that we publish. This form provides structure for consistency and transparency in reporting. For further information on Nature Portfolio policies, see our Editorial Policies and the Editorial Policy Checklist.

Statistics

For all statistical analyses, confirm that the following items are present in the figure legend, table legend, main text, or Methods section.

n/a	Confirmed	
☐	☒	The exact sample size (*n*) for each experimental group/condition, given as a discrete number and unit of measurement
☐	☒	A statement on whether measurements were taken from distinct samples or whether the same sample was measured repeatedly
☐	☒	The statistical test(s) used AND whether they are one- or two-sided<br>*Only common tests should be described solely by name; describe more complex techniques in the Methods section.*
☐	☒	A description of all covariates tested
☐	☒	A description of any assumptions or corrections, such as tests of normality and adjustment for multiple comparisons
☐	☒	A full description of the statistical parameters including central tendency (e.g. means) or other basic estimates (e.g. regression coefficient) AND variation (e.g. standard deviation) or associated estimates of uncertainty (e.g. confidence intervals)
☐	☒	For null hypothesis testing, the test statistic (e.g. F, t, r) with confidence intervals, effect sizes, degrees of freedom and P value noted<br>*Give P values as exact values whenever suitable.*
☒	☐	For Bayesian analysis, information on the choice of priors and Markov chain Monte Carlo settings
☒	☐	For hierarchical and complex designs, identification of the appropriate level for tests and full reporting of outcomes
☐	☒	Estimates of effect sizes (e.g. Cohen's *d*, Pearson's *r*), indicating how they were calculated

Our web collection on statistics for biologists contains articles on many of the points above.

Software and code

Policy information about availability of computer code

Data collection	DNA Prep for PacBio<br>* Pacific Biosciences Smart Link v10.2 (https://www.pacb.com/support/software-downloads/)<br><br>DNA Prep for Optical Map<br>* Bionano Solve v3.71 (https://bionanogenomics.com/support/software-downloads/)
Data analysis	Assembly<br>* Minimap2 v2.17 (https://github.com/lh3/minimap2)<br>* HiCANU v2.2 (https://github.com/marbl/canu)<br>* HiFiASM v0.16 (https://hifiasm.readthedocs.io/en/latest/index.html)<br>* Wengan v0.2 (https://github.com/adigenova/wengan)<br>* Bionano Hybrid Scaffolding pipeline v1.7 (https://bionanogenomics.com/support/software-downloads/)<br>* PolyPolish v0.50 (https://github.com/rrwick/Polypolish)<br>* BUSCO v5.3.2 (https://busco.ezlab.org/)<br><br>* Hi-C analysis<br>* HiCExplorer v3.7.2 (https://hicexplorer.readthedocs.io/en/latest/)<br>* BWA v0.7.17 (https://github.com/lh3/bwa)<br><br>3D modeling<br>* PASTIS v0.5.0 (https://github.com/hiclib/pastis/)<br>* ChimeraX v1.3 (https://www.cgl.ucsf.edu/chimerax/)

PacBio IsoSeq
* PacBio cDNA_Cupcake v28.0.0 (https://github.com/Magdoll/cDNA_Cupcake)

Annotation
* RepeatMasker v4.0.6 (http://repeatmasker.genome.washington.edu/)
* FunAnnotate v1.8.9 (https://funannotate.readthedocs.io/en/latest/)
* InterProScan v5.55-88 (https://www.ebi.ac.uk/interpro/download/)

Synteny and gene localization plots
* mummer2circos docker image 9/7/2021 (https://github.com/metagenlab/mummer2circos)
* Circos v0.69-8 (http://circos.ca/)
* Biopython v1.79 (https://biopython.org/)
* Gene localization custom scripts (https://github.com/ucrbioinfo/GeneLocalizationBduncani)

Orthology detection
* OrthoMCL v6.9 (https://orthomcl.org/)
* OrthoMCL mapping pipeline implemented as a Galaxy workflow (https://orthomcl.org/orthomcl/app/galaxy-orientation)
* Database searches were conducted in:
  * VEuPathDB releases 56 and 57 (https://VEuPathDB.org)
  * PiroplasmaDB releases 56 and 57 (https://PiroplasmaDB.org)
  * PlasmoDB releases 56 and 57releases 56 and 57 (https://PlasmoDB.org)
  * ToxoDB releases 56 and 57 (https://ToxoDB.org)
  * CryptoDB releases 56 and 57 (https://CryptoDB.org)
* Venn diagrams of orthology group overlap InteractiVenn (http://www.interactivenn.net)

RNA-Seq processing and analysis
* FastQC v0.11.8 (https://github.com/s-andrews/FastQC)
* Trimmomatic v0.39 (https://github.com/timflutre/trimmomatic)
* Sickle v1.33 (https://github.com/najoshi/sickle)
* HISAT2 v2.2.1 (http://daehwankimlab.github.io/hisat2/)
* Samtools v1.11 (https://github.com/samtools/samtools)
* StringTie v2.2.1 (https://github.com/gpertea/stringtie)
* TopGO v2.40.0 (https://bioconductor.org/packages/release/bioc/html/topGO.html)
* Custom scripts (https://github.com/Sabel14/Babesia_RNAseq_2022)

Drug efficacy study, Assessment of drug cytotoxicity, Steady-state kinetics, half-maximal inhibitory concentration
* Graph Pad Prism v9.2.1 (https://www.graphpad.com/)

Phylogenetic tree reconstruction
* MUSCLE v5.1 (https://github.com/rcedgar/muscle)
* MEGA X v11 (https://www.megasoftware.net/)

Gene orthology based classification analysis
*MAFFTv7.505 (https://mafft.cbrc.jp/alignment/software/)
* UpSetR v1.4.0 (https://cran.r-project.org/web/packages/UpSetR/index.html)
*ProteinOrtho v6 (https://gitlab.com/paulklemm_PHD/proteinortho/)
* Raxml v8 (https://cme.h-its.org/exelixis/web/software/raxml/)
* iTOL v6 (https://itol.embl.de/)

For manuscripts utilizing custom algorithms or software that are central to the research but not yet described in published literature, software must be made available to editors and reviewers. We strongly encourage code deposition in a community repository (e.g. GitHub). See the Nature Portfolio guidelines for submitting code & software for further information.

Data

Policy information about availability of data

All manuscripts must include a data availability statement. This statement should provide the following information, where applicable:

- Accession codes, unique identifiers, or web links for publicly available datasets
- A description of any restrictions on data availability
- For clinical datasets or third party data, please ensure that the statement adheres to our policy

All the datasets generated for the current study are available in the NCBI/SRA repository, as Bioproject PRJNA821606, as follows
* PacBio HiFi reads (SRA accession number SRR18778747)
* B. duncani genome assembly (NCBI Genome submission: SUB11253661)
* RNA-Seq (SRA accession number SRR18907291)
* RNA Iso-Seq (SRA accession number SRR18902718)
* Hi-C reads (SRA accession number SRR19325692)

Field-specific reporting

Please select the one below that is the best fit for your research. If you are not sure, read the appropriate sections before making your selection.

☒ Life sciences ☐ Behavioural & social sciences ☐ Ecological, evolutionary & environmental sciences

For a reference copy of the document with all sections, see nature.com/documents/nr-reporting-summary-flat.pdf

Life sciences study design

All studies must disclose on these points even when the disclosure is negative.

Sample size	Different biological samples from the same Babesia duncani WA1 strain were used for each of the following applications: PacBio HiFi sequencing, Nanopore sequencing (but not included in the study), Illumina sequencing, IsoSeq, RNA-Seq, Hi-C, Bionano optical map.
Data exclusions	No data was excluded from the analysis.
Replication	Each of the drug efficacy experiment was repeated three times for each compound, with each experiment including three biological replicates.
Randomization	No animal study, clinical data, or study/ies that require randomization was conducted.
Blinding	No animal study, clinical data, or study/ies that require blinding was conducted.

Reporting for specific materials, systems and methods

We require information from authors about some types of materials, experimental systems and methods used in many studies. Here, indicate whether each material, system or method listed is relevant to your study. If you are not sure if a list item applies to your research, read the appropriate section before selecting a response.

Materials & experimental systems

n/a	Involved in the study	
☐	☒	Antibodies
☐	☒	Eukaryotic cell lines
☒	☐	Palaeontology and archaeology
☒	☐	Animals and other organisms
☒	☐	Human research participants
☒	☐	Clinical data
☒	☐	Dual use research of concern

Methods

n/a	Involved in the study	
☐	☒	ChIP-seq
☒	☐	Flow cytometry
☒	☐	MRI-based neuroimaging

Antibodies

Antibodies used	anti-H3K9me3 (abcam; catalog #ab8898), anti-H3K14ac (diagenode; catalogue #C15210005)
Validation	*Describe the validation of each primary antibody for the species and application, noting any validation statements on the manufacturer's website, relevant citations, antibody profiles in online databases, or data provided in the manuscript.*

Eukaryotic cell lines

Policy information about cell lines

Cell line source(s)	HeLa (CCL-2) from ATCC, Hep G2 [HEPG2] from ATCC, HEK293T (CRL-11268) from ATCC, HCT116 (CCL-247) from ATCC.
Authentication	Authenticated cell lines were purchased from ATCC.
Mycoplasma contamination	All cell lines are negative of Mycoplasma contamination.
Commonly misidentified lines (See ICLAC register)	*Name any commonly misidentified cell lines used in the study and provide a rationale for their use.*

ChIP-seq

Data deposition

☐ Confirm that both raw and final processed data have been deposited in a public database such as GEO.

☒ Confirm that you have deposited or provided access to graph files (e.g. BED files) for the called peaks.

Data access links<br>*May remain private before publication.*	Data provided in the excel spreadsheer format.
Files in database submission	*Provide a list of all files available in the database submission.*
Genome browser session<br>(e.g. UCSC)	*Provide a link to an anonymized genome browser session for "Initial submission" and "Revised version" documents only, to enable peer review. Write "no longer applicable" for "Final submission" documents.*

Methodology

Replicates	*Describe the experimental replicates, specifying number, type and replicate agreement.*
Sequencing depth	*Describe the sequencing depth for each experiment, providing the total number of reads, uniquely mapped reads, length of reads and whether they were paired- or single-end.*
Antibodies	*Describe the antibodies used for the ChIP-seq experiments; as applicable, provide supplier name, catalog number, clone name, and lot number.*
Peak calling parameters	*Specify the command line program and parameters used for read mapping and peak calling, including the ChIP, control and index files used.*
Data quality	*Describe the methods used to ensure data quality in full detail, including how many peaks are at FDR 5% and above 5-fold enrichment.*
Software	*Describe the software used to collect and analyze the ChIP-seq data. For custom code that has been deposited into a community repository, provide accession details.*
