# Supplementary material for: Babesia duncani multi-omics identifies virulence factors and drug targets
Source: Nat Microbiol. 2023 Apr 13;8(5):845–59. doi: 10.1038/s41564-023-01360-8 (PMC10159843; doi:10.1038/s41564-023-01360-8)
Supplement: Supplementary file 5 — Unprocessed PFGE and Southern-blot images. [file 41564_2023_1360_MOESM5_ESM.pdf]

Pulsed-field gel electrophoresis (PFGE)

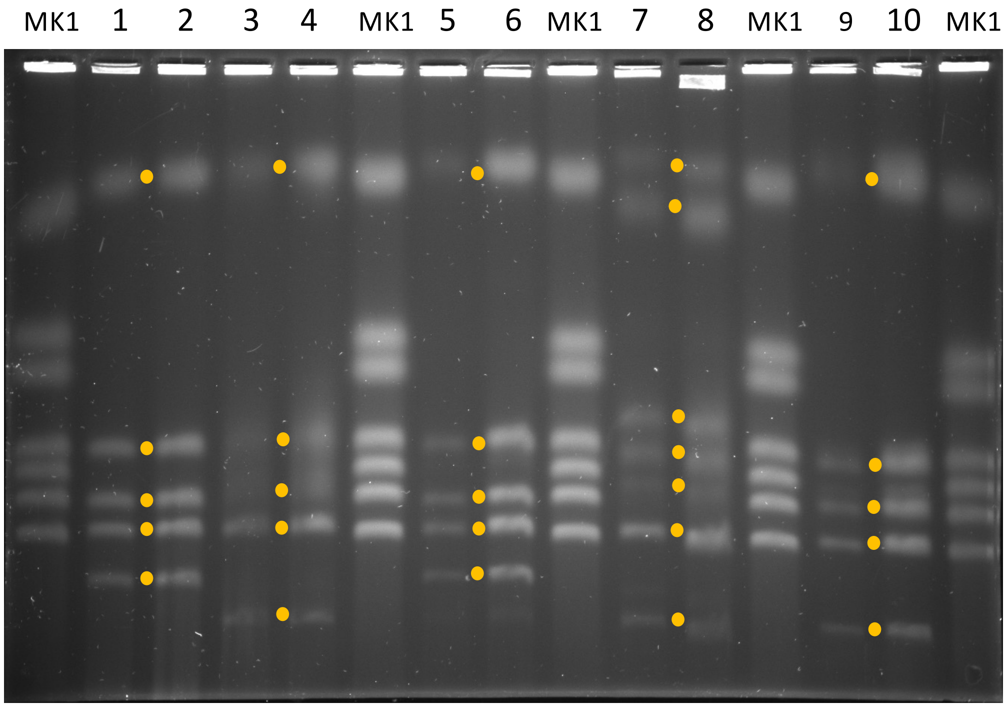

MK1 : *Hansenula wingei* chromosomes
1-2 : *B. duncani* WA1 Clone 1 (biological replicates)
3-4 : *B. duncani* WA1 Parental isolate (biological replicates)
5-6 : *B. duncani* clone A6 (biological replicates)
7-8 : *B. duncani* clone B11 (biological replicates)
9-10 : *B. duncani* clone C1 (biological replicates)

Southern blot

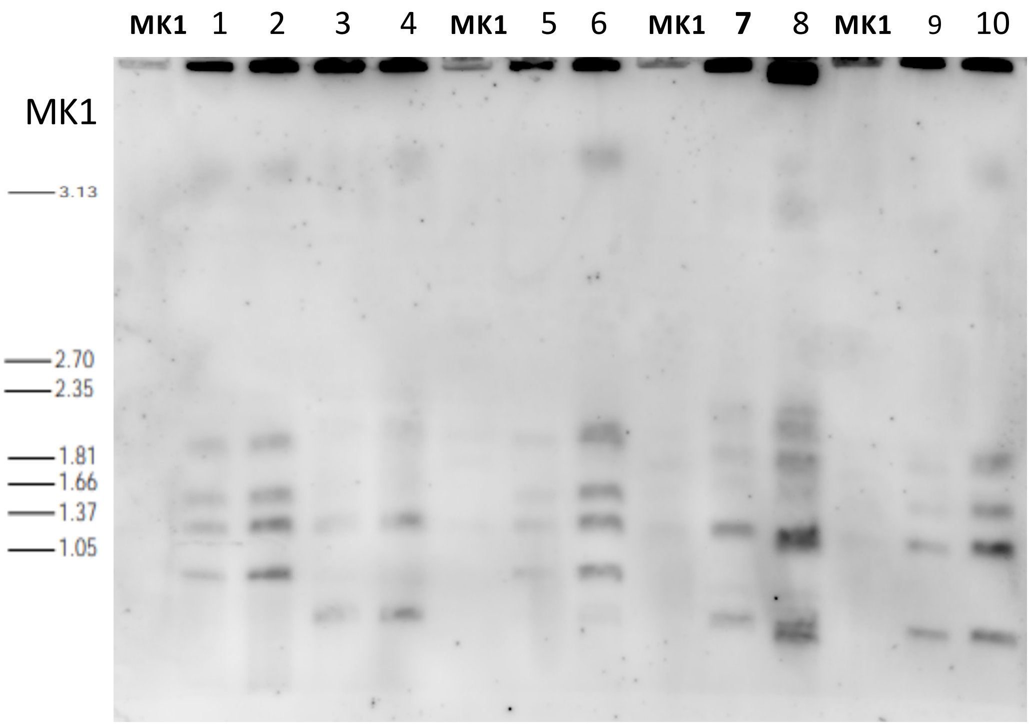

MK1 : *Hansenula wingei* chromosomes
1-2 : *B. duncani* WA1 Clone 1 (biological replicates)
3-4 : *B. duncani* WA1 Parental isolate (biological replicates)
5-6 : *B. duncani* clone A6 (biological replicates)
7-8 : *B. duncani* clone B11 (biological replicates)
9-10 : *B. duncani* clone C1 (biological replicates)
