# Supplementary material for: Babesia duncani multi-omics identifies virulence factors and drug targets
Source: Nat Microbiol. 2023 Apr 13;8(5):845–59. doi: 10.1038/s41564-023-01360-8 (PMC10159843; doi:10.1038/s41564-023-01360-8)
Supplement: Supplementary file 3 — Peer Review File [file 41564_2023_1360_MOESM3_ESM.doc]

Peer Review Information

**Journal: Nature Microbiology**

**Manuscript Title:** Babesia duncani multi-omics identifies virulence factors and drug targets.

**Corresponding author name(s): Dr Choukri Ben Mamoun, Karine G. Le Roch, Stefano Lonardi**

**Decision Letter, initial version:**

| **Message:** | 2nd August 2022  *Please ensure you delete the link to your author homepage in this e-mail if you wish to forward it to your co-authors.  Dear Dr Ben Mamoun,  Thank you for your patience while your manuscript "Decoding the Nuclear Genome of the Human Pathogen Babesia duncani Shed Light on its Virulence, Drug Susceptibility and Evolution Among Apicomplexa" was under peer-review at Nature Microbiology. It has now been seen by 4 referees, whose expertise and comments you will find at the end of this email. Although they find your work of some potential interest, they have raised a number of concerns that will need to be addressed before we can consider publication of the work in Nature Microbiology.  In particular, referee #1 has some concerns over the level of novelty provided by the study and especially by the genome sequence, and referee #2 states that the manuscript has significant shortcomings, especially with regards to the phylogenetic/evolutionary analysis. While the referees feel that the genome sequence will be of interest to the community, referee #2 says that “This makes the analysis of the genome of this piroplasmid species especially interesting and opens the possibility to compare piroplasmid genomes between each of the six reported piroplasmid lineages infecting domestic animals and/or man. It is unfortunate that the authors miss this point and its inclusion would strengthen the manuscript considerably.” Editorially, we would require this analysis to be included in a revised version of the study. Furthermore, referee #3 asks how readily drug resistance can be induced in vitro, and editorially, we would need this to be tested experimentally. We will also require all other referee comments to be addressed in full, and I should note that we would only consider sending the manuscript back for peer review if we felt that all concerns had been addressed in full. We understand that addressing all referee comments will likely take some considerable time, and if you prefer, I am also happy to consult with our colleagues at Nature Communications and Communications Biology for their potential interest in this study.  However, as mentioned, should further experimental data and text modifications allow you to address these criticisms in full, we would be happy to look at a revised manuscript.  We are committed to providing a fair and constructive peer-review process. Please do not hesitate to contact us if there are specific requests from the reviewers that you believe are technically impossible or unlikely to yield a meaningful outcome.  We strongly support public availability of data. Please place the data used in your paper into a public data repository, if one exists, or alternatively, present the data as Source Data or Supplementary Information. If data can only be shared on request, please explain why in your Data Availability Statement, and also in the correspondence with your editor. For some data types, deposition in a public repository is mandatory - more information on our data deposition policies and available repositories can be found at https://www.nature.com/nature-research/editorial-policies/reporting-standards#availability-of-data.  Please include a data availability statement as a separate section after Methods but before references, under the heading "Data Availability”. This section should inform readers about the availability of the data used to support the conclusions of your study. This information includes accession codes to public repositories (data banks for protein, DNA or RNA sequences, microarray, proteomics data etc…), references to source data published alongside the paper, unique identifiers such as URLs to data repository entries, or data set DOIs, and any other statement about data availability. At a minimum, you should include the following statement: “The data that support the findings of this study are available from the corresponding author upon request”, mentioning any restrictions on availability. If DOIs are provided, we also strongly encourage including these in the Reference list (authors, title, publisher (repository name), identifier, year). For more guidance on how to write this section please see: http://www.nature.com/authors/policies/data/data-availability-statements-data-citations.pdf   When revising your manuscript:  * Include a “Response to referees” document detailing, point-by-point, how you addressed each referee comment. If no action was taken to address a point, you must provide a compelling argument. This response will be sent back to the referees along with the revised manuscript.  * If you have not done so already we suggest that you begin to revise your manuscript so that it conforms to our Article format instructions at http://www.nature.com/nmicrobiol/info/final-submission. Refer also to any guidelines provided in this letter.  * Include a revised version of any required reporting checklist. It will be available to referees (and, potentially, statisticians) to aid in their evaluation if the manuscript goes back for peer review. A revised checklist is essential for re-review of the paper.   When submitting the revised version of your manuscript, please pay close attention to our href="https://www.nature.com/nature-portfolio/editorial-policies/image-integrity">Digital Image Integrity Guidelines.</a> and to the following points below:  -- that unprocessed scans are clearly labelled and match the gels and western blots presented in figures. -- that control panels for gels and western blots are appropriately described as loading on sample processing controls -- all images in the paper are checked for duplication of panels and for splicing of gel lanes.  Finally, please ensure that you retain unprocessed data and metadata files after publication, ideally archiving data in perpetuity, as these may be requested during the peer review and production process or after publication if any issues arise.   [redacted]  <strong>Note:</strong> This url links to your confidential homepage and associated information about manuscripts you may have submitted or be reviewing for us. If you wish to forward this e-mail to co-authors, please delete this link to your homepage first.  Nature Microbiology is committed to improving transparency in authorship. As part of our efforts in this direction, we are now requesting that all authors identified as ‘corresponding author’ on published papers create and link their Open Researcher and Contributor Identifier (ORCID) with their account on the Manuscript Tracking System (MTS), prior to acceptance. This applies to primary research papers only. ORCID helps the scientific community achieve unambiguous attribution of all scholarly contributions. You can create and link your ORCID from the home page of the MTS by clicking on ‘Modify my Springer Nature account’. For more information please visit please visit <a href="http://www.springernature.com/orcid">www.springernature.com/orcid</a>.  If you wish to submit a suitably revised manuscript we would hope to receive it within 6 months. If you cannot send it within this time, please let us know. We will be happy to consider your revision, even if a similar study has been accepted for publication at Nature Microbiology or published elsewhere (up to a maximum of 6 months).  In the meantime we hope that you find our referees' comments helpful.  [redacted]  ***************************************************** Reviewer Expertise:  Referee #1: Parasite genomics Referee #2: Babesiosis Referee #3: Babesiosis Referee #4: Babesiosis  Reviewer Comments:  Reviewer #1 (Remarks to the Author):  This paper is a description of the Babesia duncani genome sequence, coupled with some experimental characterisation of a hypothetical drug target. The genome sequencing, annotation and analysis is done in a conventional way and very well, and the manuscript is written to a very high standard.  The study provides new information on Babesia duncani, which is a rare, if unusually virulent, human pathogen. The paper describes its genome structure, gene content, and phylogeny. In all these respects, I found the genome sequence to be unremarkable and certainly not substantially different to other Babesia genomes. Thus, the paper provides an incremental corroboration of the consensus on apicomplexan genomics. The authors point to an excess of tandem gene arrays, but this may reflect the higher contiguity of this sequences and the use of long reads.  The authors also focus on the multi-copy gene families, typically surface-associated and restricted in taxonomic distribution to a lesser or greater extent. Some of these are novel, in that they are unique to B. duncani, but similar phenomena nearly always are observed in apicomplexan genomes, if not all parasite genomes, because such genes diverge very fast. The paper is quiet on Variant Erythrocyte Surface Antigens (VESA), which are the major surface antigens in other Babesia sensu stricto genomes, I'm not sure why when they are critical to immune evasion. As far as I can see, the authors have not double-checked that none of their gene families do not correspond to VESA. I am inclined to think some of them will. The methods used to characterise and compare gene families here (OrthoMCL) will not cluster VESAs from different species together due to rapid divergence resulting in mutually exclusive repertoires. Thus, B. duncani VESAs would look species-specific. More sensitive comparisons (HMMs, alphafold) are required to expose homology. But in terms of the genomic disposition, as the authors suggest, some of these gene families look like VESAs. I tried to check myself but none of the sequences or assemblies are available via the NCBI database accessions provided.  I think there is a missed opportunity here to extend the genome sequence in a genuinely novel way, that is, to look at chromatin structure in concert with the expression of these arrayed, parasite-specific genes (monoallelic expression probably?) in vitro, or at the single-cell level, or better in vivo. This would add a new and valuable perspective for Babesia.  Overall, this is rigorous science that has created a high quality resource for B. duncani, but the findings themselves are quite familiar. The work to characterise the drug target is the most innovative and valuable part but needs to be pursued further, as the authors discuss, to generate substantial impact.   Reviewer #2 (Remarks to the Author):  General comment The key result is the description of the genome structure of this Babesia parasite species, which is analyzed in high quality and should be a valuable resource for the scientific community. It is not clear if the genome will be uploaded in GenBank or another accessible public repository with publication of the manuscript. Few human cases are caused by B. duncani compared to B. microti. Unfortunately, authors try to make the reader without previous knowledge of human babesiosis believe that there is a high prevalence, though it is, in comparison to B microti, negligible. However, the fact that the parasite can be cultured in human red blood cells in vitro and causes also a fulminant infection in mice make this parasite a particularly interesting model for research in human babesiosis. An interesting feature is the presence of a var (Plasmodium) and VESA (Babesia bovis) related gene family possibly responsible for antigenic variation in B. duncani. Authors suggest that the described gene family in B. duncani may be responsible for the high virulence of this parasite in humans. Interestingly, corresponding gene families seem not to be not present in some other Babesia parasite species (e.g. B. bigemina). Another interesting feature of this manuscript is that authors identify and test possible drugs against targets encoded by the parasite genome in vitro. Noteworthy, at least six phylogenetic lineages of Piroplasmida are well known. Importantly, B. duncani belongs to the piroplasmid lineage commonly referred to as Clade II (or Western clade) and so far no parasite genome from this lineage is available. This makes the analysis of the genome of this piroplasmid species especially interesting and opens the possibility to compare piroplasmid genomes between each of the six reported piroplasmid lineages infecting domestic animals and/or man. It is unfortunate that the authors miss this point and its inclusion would strengthen the manuscript considerably. In this context, it must be mentioned that the manuscript is misleading, incompetent, and incomplete with respect to most of its content that is related to evolution or phylogeny. Important references to the literature on phylogeny of Piropasmida have been ignored. The reviewer will mention these in the following: A number of findings that authors claim to report here for the first time are not original: the placement of Babesia (correct would be Piroplasmida) among Apicomplexa is already well known and has been established by others. Authors do not add to this knowledge but at best confirm what has been already established. Thus, while there is merit in comparing the shared (or not shared genes) with other distantly related Apicomplexa parasites, such as Plasmodium, Toxoplasma etc., the manuscript does not add to already known phylogenetic/evolutionary relation of Piroplasmida with respect to distantly Apicomplexan taxons. Authors completely miss the point that what is of real interest is the relation of B. duncani within Piroplasmida, which is composed of at least six major lineages (Clade I to VI). This approach would at least have the potential to confirm previously reported placement of B. duncani among Piroplasmida. The message is here: before locating the earth with respect to other galaxies, first locate it with respect to the solar system. It comes with no surprise that all these shortcomings are accompanied by serious flaws in the tree presentation. The tree presentation suggests that Eimeria and Toxoplasma represent outgroups distantly related to all other taxons, though this is wrong. On the other hand, if authors by mistake forgot to mention in the legend that the tree is unrooted, then the tree cannot tell anything about evolutionary time. Taxon sampling (a number of Piroplasmid taxon representive for each lineage are not included in the tree!) is incomplete and therefore authors cannot confirm the reported relation of B. duncani with respect to the six well established phylogenetic lineages of Piroplasmida (Schnittger et al. 2012, 2022). Thus the phylogenetic findings reported are not original and, because they have many flaws, they can neither confirm what is already known. In addition, their findings are often overinterpreted and important references are missing. That is, their findings are not discussed in the context what is already known. This needs to be changed before publication is possible.  A number of Figure and Table numberings are wrong: please correct… Title Line 1-2 change to “…Babesia duncani sheds light…” With respect to the title “…evolution among apicomplexa” is misleading. The authors insinuate they reveal the evolutionary/phylogenetic relation of Babesia within apicomplexa. This is absolutely not true since the placing of Piroplasmida/Babesia within Apicomplexa is very well established and known and this manuscript does not add to this knowledge. I strongly recommend to change the title to “…evolution within Piroplasmida” and make reference to the phylogenetic placing of Babesia duncani in the context of the six well established phylogenetic lineages of Piroplasmida (Schnittger et al. 2012, 2022, Baneth et al 2015. Although the trees that they currently show in the manuscript do not show the placement of B. duncani in their tree; they should includ relevant taxons from each of the six phylogenetic lineages (Clade I to VI).  Introduction Line 69 -72 It would be important to make reference here to the known six phylogenetic lineages of Piroplasmida infecting domestic animals and man (Clade I to VI), Schnittger et al (2012) and which are still valid. In this study, B. duncani has been reported to belong to the phylogenetic lineage of piroplasmids of Clade II. Importantly, Baneth et al. (2020) showed that within Babesia spp. of Clade II, B. duncani is most closely related to the recently reported Babesia negevi, but not to B. conradae, using 18S RNA and mitochondrial genes. The author should mention this in the introduction since this is an important finding. This finding has been confirmed also by Schnittger et al. (2022). The authors should integrate the abovementioned findings. It is important to realize that the study of Virji et al (2018) due to the low number of samples does not allow to make statements such as that “B. duncani represents a novel lineage”. By means of a simple example, if the tree of Virji et al (2018) does not even include Cytauxzoon nor Babesia conradae, how can they claim that B. duncani is different from Cytauxzoon or B. conradae (and any other Piroplasmid species that has not been included in the tree?). Thus, the above statement is a typical example of overinterpretation. Furthermore, molecular evolutionary considerations allow to predict that a tree based on mitochondrial genes is not as suitable for deep phylogeny then the often used 18S RNA gene, since mitochondrial genes have an up to 20 time higher mutation rate.  Line 124 “….Hi-C analysis. We…” Line 195-197 as outlined above the tree in its present form is not suitable for such claims. Line 201 to 204 as stated above, the tree representation is wrong (there is no outgroup) and does not allow such a claim since taxons from a number of Piroplasmid lineage are missing (e.g. Theileria equi (Clade V), Cytauxzoon (Clade III)). Please include a species from each phylogenetic lineage and designate them with Clade I to VI. In order to improve the tree, include only Plasmodium as an outgroup and take out all other Apicomplexa such as Crypto, Eimeria, Toxoplasma. I anticipate that the tree will then improve. Line 271 Neither “spp.” nor “sp.” are not written in italics. Thoroughly search the manuscript for this Line 300 Please change “in silico” in italics. Line 310 there is a mistake with the chemical equation: please correct! Line 312 Apicomplexa is never written in italics. Please change. Line 334 change “in vitro” to italics Line 354 “the” should not be in italics. Line 360 change to: “None of these features were detecte…” Line 367 change to “…the parasite was less susceptible…” Line 389 to 390 Again a gross overinterpretation. It is incorrect or at least meaningless to say: “Recent studies have shown that this parasite is unique among hematozoa”. Note, that each parasite species belonging to hematozoa is unique within hematozoa! It is correct to say that “Within Piroplasmida this species belongs to Clade II and shows the following features that make it unique in comparison to other known species of this group.” Line 398 Please introduce “ICIM”. Search throughout the manuscript since a number of abbreviations have not been correctly introduced. Lien 404 Please introduce “hERT” Line 408 change to “…families that could be important in…” Line 415 Please introduce “CA” Line 417 Change “Babesia” into italics Line 423 Apicomplexa never in italics. Line 422 to 428 Again a gross overinterpretation; In Figure S3 (in the texts its Figure S2, please correct) they compare only some Babesia and Theileria species but claim to demonstrate evolution among Apicomplexa. There is a ongoing confusion by authors on phylogeny and evolution. Again not mentioned by authors, but this tree is unrooted (this needs to be mentioned in the legend!) and does therefore not allow interpretation based on evolutionary time but only on how closely related taxons are. Therefore, it is wrong to say “B. duncani is at the root of true Babesia”. Correct is B. duncani is most closely related (sister taxon) to Babesia sensu stricto. Please designate in the tree which are Babesia sensu stricto taxons (=Clade VI). Babesia microti belongs to a lineage of Piroplasmidais that is sister to all other Piroplasmida lineages. It would have been better to include B. microti in this analysis since then your tree would have an outgroup. Cornilleut et al (2012) is the wrong reference here since the incomplete number of taxons in the tree of the corresponding publication does not allow to interprete that B. microti is sister to all other Piroplasmida lineages. This is again an example of a serious overinterpretation. The manuscript suffers from self-citation which claim original findings while ignoring important references that reported original findings. Besides misinterpretation of their trees, authors also overinterprete genomic studies. Please note, a phylogenomic comparison cannot claim statements beyond the taxons it has included. This tells basic scientific reasoning. As to the reviewers knowledge, so far no phylogenomic comparison has added knowledge on phylogenetic relations within Piroplasmida with respect to what has been previously already known based on the use of single genes as phylogenetic markers. Line 429 Please do not use Theileridae and Babesidae in this manuscript. It sounds very educated but nobody knows to which group of parasites each term refers (e.g. Babesidae: Babesia microti (Clade I)?, Babesia bovis (Clade VI)?, Babesia duncani (Clade II)?; Theileridae: Theileria equi (Clade IV)? Theileria annulata (Clade V)?). And what about Cytauxzoon, which also belong to piroplasmida (Clade III). Each of this species corresponds to another phylogenetic lineages and to allow to distinguish between them they have been designated Clade I to VI. Line 534 Delete: “Created with Biorender.com” Line 561 Change “Babesia” into italics    Reviewer #3 (Remarks to the Author):  The manuscript by Lonardi et al, ‘Decoding the nuclear genome of the human pathogen Babesia duncani shed light on its virulence, drug susceptibility and evolution among Apicomplexa’ is a new, detailed genomic study of an emerging zoonotic pathogen transmitted by ticks. I would like to commend the authors on the high quality (PacBio and HiSeq) genome and transcriptome data as well as the use of cutting-edge chromatin structure resolution techniques. Having good ‘omics data on emerging pathogens is critical to understand and tackle the diseases they cause. These ‘omic approaches described in the manuscript have generated a large pool of data rich for interpretation, and for drawing comparison to related pathogens in the phylum of Apicomplexa. A thorough exploration from multi-gene families to energy generation based on transcription data, to potential drug target discovery followed. A wealth of information has been described in this manuscript exploring a novel human pathogen.  Major comments: •Fig 3A: representation is too small and lacks for that reason clarity. Maybe a representation as table would be more helpful. Given that both unique and conserved genes are distributed across all chromosomes the purpose of a visual representation seems unnecessary. •Fig3B: Venn diagram is too complicated and figure legend is not helpful in interpreting the diagram. Of the 1943 orthologues genes, what does the figure of 155 present in the B.bovis only circle? And what does the figure in brackets below the species name mean? What do the 5, 7, 4, 15 etc mean and to which cross-section do they belong. This data needs clarification and simplification. Same goes for Fig3C. •Fig3D: correct multiple errors relating to chromosome numbers and potentially #genes. •Fig 4: increase species and chromosome numbers on outer circle so that they become legible. •Fig5 => orthologue groups: o Lack of description of orthologue groups, and which genes belong two which group in the supplementary Excel file needs correction. A column within the Excel file with gene IDs of different species’ orthologues should also be provided in the supplementary. In addition any information of protein localisation, predicted or confirmed protein function of orthologues from different species should be listed here too. oFig5A: what is the x-axis dimension? Level of transcription? oGene product models of different orthologue families should be provided. What do the 51 genes in UMGF1 have in common? Graphical representation might be the best way to depict this. oFig5C is too small. Also where are the genes belonging to UMGF2 on the graphic? GPI-anchored gene products are unnecessarily displayed here, I would argue. The distribution of GPI-anchored gene products across all chromosomes is not really surprising, and does not need emphasis. Maybe to allow better visualisation, the authors could provide this graphical information of gene locations on chromosomes in a larger, separate supplementary figure, linked to the Excel sheet? o“genes in the BdUMGF family are not only in closed proximity to each other at the chromatin 3D level…” should be visually demonstrated on a chromatin blot based on the colours chosen for the families in Fig5. Are dark blue centrally localised UMGF3 genes adjacent to red subtelomeric UMGF1 genes in this chromatin blot given they are both transcribed at low level? Where in the chromatin structure of the nucleus are these gene families located? DNA and RNA-Fish data would also have been useful here. o“considering the similarity observed between the var and BdUMGF gene families at the chromatin 3D and gene expression level, it is likely that genes that belong to BdUMGF are involved in antigenic variation…” is a strong statement, based a limited data presented here. This needs experimental validation. Do the authors say that all BdUMGF families are transcriptionally repressed? Where can the reader see this in Fig 5A? Do the gene products show similarities across the different UMGF groups and do they have conserved features when looking at well characterised genes involved in antigenic variation (var, vir, Pfam2TM, ves1)? To speculate on antigenic variation based on the subtelomeric location of gene families and low transcription levels is overinterpreted and needs to be substantiated experimentally. For example, single cell RNAseq on B.duncani isolated from hamsters would be able to demonstrated single, or small numbers of genes from individual UMGF groups transcribed and which genes are transcribed would be different in different cells. I would encourage the authors to conduct these experiments to prove their hypothesis of antigenic variation in B. duncani. oLines 356-359: discusses gene repression on telomeric ends of chr 1, 3, 4 and 5 and links this with the localisation of genes belonging to the BdUMGF1 multigene family. However, there appears to be no UMGF1 gene family members localized to chromosome 3 so why is the repression seen there? Could it be that there is generally repression of transcription in genes localised in telomeric regions rather than the repression being linked to genes hypothesised to be involved in antigenic variation?  Minor Comments: -Line 156 – Table I is missing. -Line 202 reads Fig3D but should be Fig3E. -Lines 216-17: large synteny is between Bd Chr4 and B. bigemina chr1 and not chr 4 as stated. -Line 288: what does ‘var gene family consists of ~7x genes’ mean? Rephrase. -Lines 317-18: BdUMGF9 group is not indicated in Fig5 which is listed here. -Line 426: there is no phylogenetic tree in Fig S3. -Fig 8F: explain in figure legend the confidence interval (+-) in the enzyme kinetics data.   Reviewer #4 (Remarks to the Author):  While this manuscript presents new and interesting data on the genomic aspects of Babesia duncani there are numerous inconsistencies, grammatical errors, and poorly described methods and results unbefitting this journal. Please see my specific comments to the pdf manuscript and additional line comments.  Grammatical errors have been added to the pdf version in red color. Please be consistent in using the Oxford comma, the meaning of the sentence is altered without it.  Introduction: Can the authors provide details about what is known on the genetic similarities and differences between Babesia species? A recent review from Goethert 2021, might provide some helpful information.  Line 57: Please be specific about which species of Babesia is being referenced here with the 9% mortality rate. Reference 4 is specific to B. microti.  Line 84: Suggest starting a new paragraph with “Despite the highly…”  Line 97: Spell out the genus name at the beginning of a sentence.  Line 97-100: It would be helpful to explain the overall results here instead of listing the items depicted in Fig 1. Can the results in Fig 1 be explained a bit more here?  Line 128: The last part of this sentence seems out of place. It would be better to end the sentence after reference 21 and begin a new sentence about the centromeres.  Line 128: What “feature” are the authors referring to? Many features were stated in the previous sentence, please be specific which one or ones this sentence is regarding. Separating the centromere comment from the previous sentence will also make it clearer that this is the feature that is important for the following sentence.  Line 129-130: Above the chromosomes are listed using roman numerals, please be consistent here and use roman numerals for chromosome number.  Line 129-131: Can the types of centromeres be explained more? What is the difference and the significance of these three types of centromeres?  Line 140: Spell out the genus name the first time it is used.  Line 174: Spell out the genus name the first time it is used.  Lines 173-177: Would be better suited in the Introduction.  Line 179: Suggest rewording the beginning of this sentence so the reader doesn’t feel commanded. Some examples: “We observed…” or “ The genome size…was higher in B. duncani compared to…”  Line 189: Bold Fig 3A to be consistent.  Line 195 and 196: orthologous groups?  Line 198: Capitalize Apicomplexa  Lines 200-201: What do the authors mean by “gene content did not reveal any surprises”? Please use more technical language. What constitutes a “surprise” for the authors?  Lines 211-217: Be consistent with chromosome numbering, roman numerals  Line 219: Italicize genus name  Line 231: Do not capitalize lactate  Line 236: Spell out CDS at first use  Line 239: Numbers should be spelled out when they start a sentence.  Lines 242-245: The authors state that 4 aspartic proteases are expressed in P. falciparum and B. duncani, are they the same aspartic proteases in both species? The following sentence lists 4 proteases and their function, but are these the same ones expressed by P. falciparum?  Lines 267 and 270: Apicomplexa should not be italicized  Line 271: Do not italicize “spp”  Line 273: Spell out genus the first time it’s used  Line 276 and 279: Be consistent with figure abbreviations (Fig. X). Also, spell out GPI and AP families on the first use.  Line 276: See above comment about starting a sentence with a command (Observe…)  Lines 278-282: Chromosome numbering  Line 283: Do the authors mean to say “close proximity”?  Line 285: Bold Fig reference to be consistent  Line 289-292: References are needed for the information about the var gene family in P. falciparum.  Line 291: Do the authors mean to say “structural changes”?  Line 286-292: Please be consistent and pay close attention to how you are writing genes and gene families (var, Var, var)  Line 298: Spell out MSP the first time it’s used  Line 306: Add references for this information (B. microti ortholog and conserved region in other piroplasmida)  Lines 309-311: Add references for this information  Line 312: Apicomplexa should not be italicized  Lines 297-317: Several sentences in this paragraph would be better suited for the Discussion section.  Line 322: Please clarify what is meant by “…tick vector families…” Ticks belong to either the Ixodidae or Argasidae family, but I’m guessing the authors mean gene families. Please reword the sentence to make this more clear.  Lines 327-329: Please clarify how capturing almost 90% of the genome indicates that most genes are needed for survival in host RBCs. How do you know some of these genes aren’t needed to survive in the tick? Or for transmission from tick-to-host? Seq should not be capitalized.  Line 339: Please be consistent with narrative, third person has been used throughout the manuscript however fist person (we) is used here.  Line 341: Capitalize and italicize genus name  Line 343: Clarify which “genes” are being reference here  Line 344: lncRNAs should be spelled out the first time it is used, not the second.  Line 347: Reference needed  Line 347: What is “their roles…” referring to? lncRNAs? It would be better to say “The roles of lncRNAs have…”  Line 349: The narrative has changed. It seems someone else wrote this section, please be consistent with tense and narrative throughout the manuscript.  Line 354: Do not italicize “the”  Lines 358-359: Chromosome numbering  Line 372: Reference needed  Line 384: Bold Fig. for consistency  Line 390: Reference needed  Line 401: Do the authors mean “studies”  Line 405: Please clarify who “they” is in this sentence.  Line 416: References needed  Line 417: Italicize genus name  Line 419: References needed  Line 423: Apicomplexa should not be italicized Line 430: References needed  Line 431: References needed  Line 435: Spell out RAP upon first use  Line 437: Spell out numbers when they start a sentence  Line 449: Spell out TFIIA upon first use  Line 483: Don’t italicize sp.  Line 512: Spell out GO upon first use. Too many spaces in front of GO?  Line 517 and 519: Italicize genus name  Line 545: Do not italicize “and”  Line 552: Spell out Bd and Bm, I assume these are B. duncani and B. microti, but these abbreviations have not been used previously.  Line 561: Italicize genus name  Materials and Methods: It is best to use past tense because the experiments have been completed.  Line 579: Add a space between B. duncani  Line 586: Suggest rewording the sentence as “…changed daily and parasitemia was monitored by…”  Line 590: 95% pure what? Please clarify. Do the authors mean to say “purity”? Or change “with” to “were”  Line 605: Do the author mean to say “move” instead of movie?  Line 607: Explain what “Yale’s gold standard” is, what are the measurements?  Line 609: Please explain the frozen pellets. Was B. duncani frozen into pellets and packed into 3ml packaging? Why? How were these frozen pellets of B. duncani used to isolate molecular weight? Please be clear about this method.  Lines 624-629: A lot of this seems like Results.  Line 677: PASTIS should be in all capital letters  Line 682: It looks like reference 42 is in a different font.  Line 695: Replace ran with run  Line 707: Why is translated capitalized? Numbers should be spelled out when at the beginning of a sentence. I suggest rewording this sentence.  Line 720: Suggest rewording “Babesia microti drastically reduced…”  Lines 736-754: Can this section be combined with the 3D modeling section on lines 673-679? If not, please explain the differences between these two 3D modeling sections.  Lines 762-763: Poorly worded sentences, please reword.  Line 767: Please be consistent, intraerythrocytic was not hyphenated previously.  Line 768: Please be consistent, references should not be italicized.  Line 770: Do not capitalize hrs. Please be consistent with the units, h, hrs, hr, hours have all been used.  The authors state in the Abstract that they performed “efficacy studies” however this was not explained in the results. How did the authors challenge a host for the efficacy studies? What host was used? Where are the details about these studies and the results? In the last section of the Results, the authors used human cell lines, but not live hosts. In the Discussion, they say their results could be used to test efficacy in mice. Please clarify if these studies were conducted and provide better methods and results of these trials.  Highlighted sentences in the Results section need to be better addressed and explained in the Discussion section.  The authors should be consistent with how they reference the figures in the figure legend. Sometimes a period is used, sometimes a colon, and sometimes a single parenthesis (A. text. B: text. C) text.).  Please check the references as several have mistakes (i.e. scientific names not italicized).  Figures 3, 4, 5: The font size is too small in several figures making it difficult to read.  Table 6: Is the SP/TM column necessary to include as no data is presented in this column? Could this be better as a footnote?  Figures S3: Scientific names should be italicized |
| --- | --- |

**Author Rebuttal to Initial comments**

**Response to reviewers:**

We thank the reviewers for their thorough review of our manuscript and for their constructive remarks. We have addressed all the comments of the reviewers, conducted all the requested experiments, added supporting data and information to strengthen our conclusions, and updated the manuscript accordingly. The Abstract, Introduction, Results, Discussion, Methods, and Supporting Information chapters were all updated. The corrections and changes are highlighted in yellow in the revised version of the manuscript, supporting information, and figures.

**Editor:**

*In particular, referee #1 has some concerns over the level of novelty provided by the study and especially by the genome sequence, and referee #2 states that the manuscript has significant shortcomings, especially with regards to the phylogenetic/evolutionary analysis. While the referees feel that the genome sequence will be of interest to the community, referee #2 says that “This makes the analysis of the genome of this piroplasmid species especially interesting and opens the possibility to compare piroplasmid genomes between each of the six reported piroplasmid lineages infecting domestic animals and/or man. It is unfortunate that the authors miss this point and its inclusion would strengthen the manuscript considerably.” Editorially, we would require this analysis to be included in a revised version of the study. Furthermore, referee #3 asks how readily drug resistance can be induced in vitro, and editorially, we would need this to be tested experimentally. We will also require all other referee comments to be addressed in full, and I should note that we would only consider sending the manuscript back for peer review if we felt that all concerns had been addressed in full.*

**Response:** This work represents the first genome discovery from a parasite belonging to clade II of piroplasmids. Our finding emphasizes the importance of *B. duncani* as an excellent model organism for human Babesiosis and other intraerythrocytic infections of humans and other mammals. The significance of model organisms in the pathogenesis of fungal infections, for example, was well established from the extensive research performed on *Saccharomyces cerevisiae*. Additionally, model systems for human diseases were previously developed in fruit fly (*Drosophila melanogaster)*, *Caenorhabditis elegans*, and zebrafish (*Danio rerio*) organisms. To date, *Toxoplasma gondii* is considered one of the model organisms for protozoan parasite-borne diseases. A recent report by Pal et al. 2022 demonstrates that *B. duncani* can serve as a model organism to study the development, virulence, and drug susceptibility of intraerythrocytic parasites *in vitro* and *in vivo*. The complete annotated *B. duncani* genome will advance the knowledge in studying the parasite cell biology and drug targetability towards the design of novel therapeutics approaches.

Furthermore, the phylogenetic tree (Figure 3C) in our study was modified and rooted according to the suggestions of the reviewers. An updated tree is now included in the manuscript (pages 12-13) and includes representative species from the six clades I-VI.

As suggested by the reviewer and highlighted in the editor’s summary, we conducted drug selection in *B. duncani* to address the specific comment as to whether drug resistant parasites could be selected in vitro. The new data in our manuscript presented in Figure S6 demonstrates the feasibility of generating *in vitro* drug resistant strains from *B. duncani* WA-1 parent strain to atovaquone and whole genome sequencing to map the specific mutation, which validates both the mechanism of action and mode of resistance to this drug. The results presented in Figure S6 show a 15.2-fold increase in IC_50_ of atovaquone in the *B. duncani* resistant clone with a single mutation in the parasite cytochrome b *BdCytb* gene responsible for the reduced susceptibility to the drug. The experimental approach used to generate the resistant clones has been added to the manuscript (pages 23-24) with methods description on supplementary information.

Additional experiments conducted including those suggested by the reviewers:

- Hi-C based 3D model (Figure 6A): in response to the reviewers’ comments about the distribution of the multigene families on the chromosomes, we used the Hi-C data to confirm the localization, which was also further supported by the FISH analysis.
- FISH analysis (Figure 6B): we designed specific probes to members of the newly discovered multigene families and demonstrated the distribution of these genes in the different chromosomes as was initially described in the original submission.
- ChIP-Seq analysis (Figure 8): Similar to the FISH data, ChIP-Seq analysis was successful and far exceeded our own expectations. It provided very clear evidence that members of the *B. duncani* multigene families are under epigenetic regulation. We have now included an additional figure summarizing these data (Figure 8). We sincerely thank the reviewers and the editor for suggesting this work.
- Drug selection *in vitro* (Figure S6): as highlighted above, we now can confirm that drug resistant parasites can be selected in vitro and assist in understanding the mode of action and resistance to antiparasitic drugs.
- Phylogenetic analysis: as mentioned above, we conducted a new analysis using a larger set of *Babesia* species including a member from each of the previously described clades of Piroplasmida.

**Reviewer #1:**

*This paper is a description of the Babesia duncani genome sequence, coupled with some experimental characterisation of a hypothetical drug target. The genome sequencing, annotation and analysis is done in a conventional way and very well, and the manuscript is written to a very high standard.*

**Response:** We would like to thank Reviewer #1 for this feedback and thorough review for our submitted manuscript.

*The study provides new information on Babesia duncani, which is a rare, if unusually virulent, human pathogen. The paper describes its genome structure, gene content, and phylogeny. In all these respects, I found the genome sequence to be unremarkable and certainly not substantially different to other Babesia genomes. Thus, the paper provides an incremental corroboration of the consensus on apicomplexan genomics. The authors point to an excess of tandem gene arrays, but this may reflect the higher contiguity of this sequences and the use of long reads.*

**Response:** The genomic analysis and cell biological studies reported in our manuscript unraveled multiple unknowns in *Babesia* biology and therapy including genomic content and 3D structure, gene composition and expression, phylogeny and a novel multigene family composition. The study further highlights the importance of genomic-based target mining to identify potential drugs that could be rapidly repurposed or developed for the treatment of human babesiosis. None of this knowledge would have been possible without this work and our understanding of the biology of this pathogen would have been based solely on assumptions most which would have been wrong. Of course *B. duncani* is an intraerythrocytic parasite and a member of the Apixomplexa phylum but that does not necessarily mean that a finding in one pathogen can be blindly extrapolated to a related pathogen. *P. falciparum* and *P. vivax* are also close relatives, yet one causes far more deaths than the other and their pathobiology and virulence are completely different. The same is true for *S. cerevisiae* and *C. albicans*, one is not pathogenic and the other is and yet they share high degree of identity and similarity in their genomic information, composition and structure. The statement that the study reflects “an incremental corroboration of the consensus on apicomplexa genomics” is an unfair statement in light of the major findings made in this paper and the fact that there is now enough evidence to suggest that *B. duncani* can serve as an excellent model to study Babesia, and intraerythrocytic parasitism in general, due to the availability of a continuous in vitro culture system as well as a mouse model of lethal infection. The information gained from this study will greatly facilitate the advancement of research on *Babesia* and other intraerythrocytic parasites. Finally, the tandem gene arrays have been validated by various analyses (Nanopore, PacBio, HiC and Optical mapping), which are all described in details in the Methods section of the paper.

*The authors also focus on the multi-copy gene families, typically surface-associated and restricted in taxonomic distribution to a lesser or greater extent. Some of these are novel, in that they are unique to B. duncani, but similar phenomena nearly always are observed in apicomplexan genomes, if not all parasite genomes, because such genes diverge very fast. The paper is quiet on Variant Erythrocyte Surface Antigens (VESA), which are the major surface antigens in other Babesia sensu stricto genomes, I'm not sure why when they are critical to immune evasion. As far as I can see, the authors have not double-checked that none of their gene families do not correspond to VESA. I am inclined to think some of them will. The methods used to characterise and compare gene families here (OrthoMCL) will not cluster VESAs from different species together due to rapid divergence resulting in mutually exclusive repertoires. Thus, B. duncani VESAs would look species-specific. More sensitive comparisons (HMMs, alphafold) are required to expose homology. But in terms of the genomic disposition, as the authors suggest, some of these gene families look like VESAs. I tried to check myself but none of the sequences or assemblies are available via the NCBI database accessions provided.*

**Response:** Our sequence analysis of the multigene families of *B. duncani* used not only OrthoMCL with the advanced knowledge that VESA genes belong to multiple orthology groups, but also Blast analyses using specific sequences of VESA genes belong to different orthology groups. These analyses showed that *B. duncani* multigene families have evolved differently from those of other parasites. However, and as the reviewer rightly indicated, genomic distribution of the BdOMGF and BdUMGFs are similar to that of other variable surface antigens from *B. bovis*, *P. falciparum* and *Theileria* species. Regarding the data availability, the genome was deposited in GenBank and will be released once the manuscript is accepted for publication. We are also happy to provide a link to the reviewers if that is needed.

*I think there is a missed opportunity here to extend the genome sequence in a genuinely novel way, that is, to look at chromatin structure in concert with the expression of these arrayed, parasite-specific genes (monoallelic expression probably?) in vitro, or at the single-cell level, or better in vivo. This would add a new and valuable perspective for Babesia.*

**Response:** We thank the reviewer for the suggestion and as requested we conducted ChIP-Seq analyses in this parasite to assess whether the newly discovered multigene families undergo gene expression regulation similar to that of other variable surface antigens from other parasites (Figure 8). The ChIPSeq analysis data of *B. duncani* presented similar trends to the ones observed in other parasites while revealing novel epigenetic insights into *Babesia* gene regulation with multigene families most likely involved in antigenic variation. The data also identified additional gene sets that are under regulation but likely expressed in the tick or other stages of the parasite life cycle. This analysis was further described in the Results (page 20-21), Discussion (page 27-28), and Methods (pages 47-50) sections.

*Overall, this is rigorous science that has created a high quality resource for B. duncani, but the findings themselves are quite familiar. The work to characterise the drug target is the most innovative and valuable part but needs to be pursued further, as the authors discuss, to generate substantial impact.*

**Response:** We agree that the newly identified drug targets and the potential drugs should be pursued further. In this study we have demonstrated that *B. duncani* DHFR-TS enzyme is a suitable target with the discovery of WR-99210 as a potent inhibitor of parasite growth (Figure 10). This class of compounds is under further investigation by several groups to identify potent and safe compounds. These studies, however, are outside the scope of this specific genomic effort. Equally important, we note that *B. duncani* could be used to select parasites that are refractory to drugs as a strategy to gain further insights in the mechanism of action and resistance of specific antiparasitic drugs. ATV^R^ resistant *B. duncani* parasites were selected in this study as a proof of feasibility of this concept. Unlike other apicomplexan parasites such as *P. falciparum*, drug resistant parasites can be generated in *B. duncani* in a relatively shorter time frame (4 weeks to 8 weeks depending on the drug). Therefore, *B. duncani* can be used as a model system to identify the targets of currently used and novel anti-malarial and anti-babesial drugs and to predict possible mechanisms of resistance.

**Reviewer #2:**

*General comment*

*The key result is the description of the genome structure of this Babesia parasite species, which is analyzed in high quality and should be a valuable resource for the scientific community.*

**Response:** We would like to thank Reviewer #2 for this feedback and thorough review for our submitted manuscript.

*It is not clear if the genome will be uploaded in GenBank or another accessible public repository with publication of the manuscript.*

**Response:** The genome was deposited in GenBank and will be released once the manuscript will be accepted for publication.

*Few human cases are caused by B. duncani compared to B. microti. Unfortunately, authors try to make the reader without previous knowledge of human babesiosis believe that there is a high prevalence, though it is, in comparison to B microti, negligible. However, the fact that the parasite can be cultured in human red blood cells in vitro and causes also a fulminant infection in mice make this parasite a particularly interesting model for research in human babesiosis. An interesting feature is the presence of a var (Plasmodium) and VESA (Babesia bovis) related gene family possibly responsible for antigenic variation in B. duncani. Authors suggest that the described gene family in B. duncani may be responsible for the high virulence of this parasite in humans. Interestingly, corresponding gene families seem not to be not present in some other Babesia parasite species (e.g. B. bigemina). Another interesting feature of this manuscript is that authors identify and test possible drugs against targets encoded by the parasite genome in vitro.*

**Response:** We agree with the reviewer that *B. microti* is responsible for the majority of the clinical cases of human babesiosis. We have clearly mentioned this fact in line 65. If the reviewer is pointing to the statement that mentions the mortality rate from *Babesia* infections (line 59), then, we have rephrased this statement to include recent numbers on human babesiosis.

*Noteworthy, at least six phylogenetic lineages of Piroplasmida are well known. Importantly, B. duncani belongs to the piroplasmid lineage commonly referred to as Clade II (or Western clade) and so far no parasite genome from this lineage is available. This makes the analysis of the genome of this piroplasmid species especially interesting and opens the possibility to compare piroplasmid genomes between each of the six reported piroplasmid lineages infecting domestic animals and/or man. It is unfortunate that the authors miss this point and its inclusion would strengthen the manuscript considerably. In this context, it must be mentioned that the manuscript is misleading, incompetent, and incomplete with respect to most of its content that is related to evolution or phylogeny. Important references to the literature on phylogeny of Piropasmida have been ignored.*

**Response:** We would like to thank reviewer #2 for this important comment. According to reviewer #2 suggestion, we have re-analyzed the phylogenetic structure of piroplasmida (Figure 3C), added new species and rooted the tree as recommended. The section of the manuscript describing the phylogenetic analysis has been updated in the manuscript and the changes are highlighted in yellow (pages 12-13). Species from each of clades I-VI were included in this analysis and are shown in the new Figure 3C. We have also incorporated the required references according to reviewer #2 suggestions.

*The reviewer will mention these in the following:*

*A number of findings that authors claim to report here for the first time are not original: the placement of Babesia (correct would be Piroplasmida) among Apicomplexa is already well known and has been established by others. Authors do not add to this knowledge but at best confirm what has been already established. Thus, while there is merit in comparing the shared (or not shared genes) with other distantly related Apicomplexa parasites, such as Plasmodium, Toxoplasma etc., the manuscript does not add to already known phylogenetic/evolutionary relation of Piroplasmida with respect to distantly Apicomplexan taxons.*

**Response:** We thank the review of the comment. Although, the point is well-taken, we argue that the available of whole genome sequences and tools to analyze global patterns of evolution could help re-evaluate phylogenetic data established on the basis of specific markers such as 18S rRNA, CytB or Tubulin to name only a few. In fact, in many cases the phylogenetic trees established based on single markers can lead to different interpretations. Whereas a genome-wide analysis such as the one offered in this manuscript using a large set of single copy genes offers an unbiased examination of the phylogeny of a given species. In this specific case, however, our data were consistent with those previously reported by Schnittger and colleagues, which describe 6 clades of piroplasmidae with *B. duncani* representing a separate and distinct clade among these. We have now revised our manuscript and analysis to address this point.

*Authors completely miss the point that what is of real interest is the relation of B. duncani within Piroplasmida, which is composed of at least six major lineages (Clade I to VI). This approach would at least have the potential to confirm previously reported placement of B. duncani among Piroplasmida. The message is here: before locating the earth with respect to other galaxies, first locate it with respect to the solar system.*

**Response:** We agree with the reviewer and have incorporated changes in the manuscript by including a new phylogenetic tree that shows the placement of *B. duncani* within Piroplasmida. The new tree contains members from each of the six clades. The text was updated accordingly in our manuscript (pages 12-13).

*It comes with no surprise that all these shortcomings are accompanied by serious flaws in the tree presentation. The tree presentation suggests that Eimeria and Toxoplasma represent outgroups distantly related to all other taxons, though this is wrong. On the other hand, if authors by mistake forgot to mention in the legend that the tree is unrooted, then the tree cannot tell anything about evolutionary time. Taxon sampling (a number of Piroplasmid taxon representive for each lineage are not included in the tree!) is incomplete and therefore authors cannot confirm the reported relation of B. duncani with respect to the six well established phylogenetic lineages of Piroplasmida (Schnittger et al. 2012, 2022). Thus the phylogenetic findings reported are not original and, because they have many flaws, they can neither confirm what is already known. In addition, their findings are often overinterpreted and important references are missing. That is, their findings are not discussed in the context what is already known. This needs to be changed before publication is possible.*

**Response:** A new rooted phylogenetic tree has been generated and incorporated in the manuscript that answers all the concerns raised by the reviewer. The tree description was adjusted in our manuscript (pages 12-13). The six piroplasmids from clades I-VI were compared in the new figure (new Figure 3C).

*A number of Figure and Table numberings are wrong: please correct…*

**Response:** Thank you for this important note. The figures and tables numbering was reviewed again and was changed accordingly.

*Title: Line 1-2 change to “…Babesia duncani sheds light…”*

**Response:** Corrected.

*With respect to the title “…evolution among apicomplexa” is misleading. The authors insinuate they reveal the evolutionary/phylogenetic relation of Babesia within apicomplexa. This is absolutely not true since the placing of Piroplasmida/Babesia within Apicomplexa is very well established and known and this manuscript does not add to this knowledge.*

*I strongly recommend to change the title to “…evolution within Piroplasmida” and make reference to the phylogenetic placing of Babesia duncani in the context of the six well established phylogenetic lineages of Piroplasmida (Schnittger et al. 2012, 2022, Baneth et al 2015. Although the trees that they currently show in the manuscript do not show the placement of B. duncani in their tree; they should includ relevant taxons from each of the six phylogenetic lineages (Clade I to VI).*

**Response:** Thank you for your comment about the manuscript title. The title was corrected accordingly.

*Introduction*

*Line 69 -72 It would be important to make reference here to the known six phylogenetic lineages of Piroplasmida infecting domestic animals and man (Clade I to VI), Schnittger et al (2012) and which are still valid. In this study, B. duncani has been reported to belong to the phylogenetic lineage of piroplasmids of Clade II. Importantly, Baneth et al. (2020) showed that within Babesia spp. of Clade II, B. duncani is most closely related to the recently reported Babesia negevi, but not to B. conradae, using 18S RNA and mitochondrial genes. The author should mention this in the introduction since this is an important finding. This finding has been confirmed also by Schnittger et al. (2022). The authors should integrate the above mentioned findings.*

**Response:** Thanks for highlighting this important observation. The suggested changes have been incorporated into the manuscript.

*It is important to realize that the study of Virji et al (2018) due to the low number of samples does not allow to make statements such as that “B. duncani represents a novel lineage”. By means of a simple example, if the tree of Virji et al (2018) does not even include Cytauxzoon nor Babesia conradae, how can they claim that B. duncani is different from Cytauxzoon or B. conradae (and any other Piroplasmid species that has not been included in the tree?). Thus, the above statement is a typical example of overinterpretation. Furthermore, molecular evolutionary considerations allow to predict that a tree based on mitochondrial genes is not as suitable for deep phylogeny then the often used 18S RNA gene, since mitochondrial genes have an up to 20 time higher mutation rate.*

**Response:** We thank the reviewer for the constructive comment. We have updated the text to highlight other studies that support that *B. duncani* represents a new lineage within piroplasmida (Schnittger et al (2012, 2022) and Baneth et al. (2020)). Moreover, we have generated a new phylogenetic tree that supports the above fact.

*Line 124 “….Hi-C analysis. We…”*

**Response:** Corrected.

*Line 195-197 as outlined above the tree in its present form is not suitable for such claims.*

**Response:** A new tree has been generated that supports these claims.

*Line 201 to 204 as stated above, the tree representation is wrong (there is no outgroup) and does not allow such a claim since taxons from a number of Piroplasmid lineage are missing (e.g. Theileria equi (Clade V), Cytauxzoon (Clade III)). Please include a species from each phylogenetic lineage and designate them with Clade I to VI. In order to improve the tree, include only Plasmodium as an outgroup and take out all other Apicomplexa such as Crypto, Eimeria, Toxoplasma. I anticipate that the tree will then improve.*

**Response:** As suggested, we have generated a new tree that includes a species from each Clade.

*Line 271 Neither “spp.” nor “sp.” are not written in italics. Thoroughly search the manuscript for this*

**Response:** Corrected.

*Line 300 Please change “in silico” in italics.*

**Response:** Corrected.

*Line 310 there is a mistake with the chemical equation: please correct!*

**Response: Corrected.**

*Line 312 Apicomplexa is never written in italics. Please change.*

**Response:** Corrected throughout the manuscript.

*Line 334 change “in vitro” to italics*

**Response:** Corrected throughout the manuscript.

*Line 354 “the” should not be in italics.*

**Response:** Corrected.

*Line 360 change to: “None of these features were detecte…”*

**Response:** Corrected.

*Line 367 change to “…the parasite was less susceptible…”*

**Response:** Corrected.

*Line 389 to 390 Again a gross overinterpretation. It is incorrect or at least meaningless to say: “Recent studies have shown that this parasite is unique among hematozoa”. Note, that each parasite species belonging to hematozoa is unique within hematozoa! It is correct to say that “Within Piroplasmida this species belongs to Clade II and shows the following features that make it unique in comparison to other known species of this group.”*

**Response:** Corrected.

*Line 398 Please introduce “ICIM”. Search throughout the manuscript since a number of abbreviations have not been correctly introduced.*

**Response:** Corrected. ICIM model referred to in culture-in mouse model.

*Lien 404 Please introduce “hERT”*

**Response:** Corrected.

*Line 408 change to “…families that could be important in…”*

**Response: Corrected.**

*Line 415 Please introduce “CA”*

**Response:** Corrected.

*Line 417 Change “Babesia” into italics*

**Response:** Corrected.

*Line 423 Apicomplexa never in italics.*

**Response:** Corrected throughout the manuscript.

*Line 422 to 428 Again a gross overinterpretation; In Figure S3 (in the texts its Figure S2, please correct) they compare only some Babesia and Theileria species but claim to demonstrate evolution among Apicomplexa. There is a ongoing confusion by authors on phylogeny and evolution.*

**Response:** We agree with the reviewer and the manuscript has been modified accordingly (page 25, from line 556).

*Again not mentioned by authors, but this tree is unrooted (this needs to be mentioned in the legend!) and does therefore not allow interpretation based on evolutionary time but only on how closely related taxons are. Therefore, it is wrong to say “B. duncani is at the root of true Babesia”. Correct is B. duncani is most closely related (sister taxon) to Babesia sensu stricto. Please designate in the tree which are Babesia sensu stricto taxons (=Clade VI).*

**Response:** A new phylogenetic tree has been generated that answers the concerns raised by the reviewer. The tree description was adjusted in our manuscript (pages 12-13). The six piroplasmids from clades I-VI were compared in the new figure (new Figure 3C).

*Babesia microti belongs to a lineage of Piroplasmidais that is sister to all other Piroplasmida lineages. It would have been better to include B. microti in this analysis since then your tree would have an outgroup. Cornilleut et al (2012) is the wrong reference here since the incomplete number of taxons in the tree of the corresponding publication does not allow to interprete that B. microti is sister to all other Piroplasmida lineages. This is again an example of a serious overinterpretation. The manuscript suffers from self-citation which claim original findings while ignoring important references that reported original findings. Besides misinterpretation of their trees, authors also overinterprete genomic studies. Please note, a phylogenomic comparison cannot claim statements beyond the taxons it has included. This tells basic scientific reasoning. As to the reviewers knowledge, so far no phylogenomic comparison has added knowledge on phylogenetic relations within Piroplasmida with respect to what has been previously already known based on the use of single genes as phylogenetic markers.*

**Response:** A new orthology-based classification (in the previous manuscript version was described as phylogenetic tree) has been generated that answers the concerns raised by the reviewer and the text has been adjusted accordingly (pages 12-13). As indicated in our previous responses, species for each of the 6 clades of piroplasmida have now been included in this analysis. Regarding the citations, we have updated the references to highlight other studies that investigated the phylogeny of piroplasmida. We would like to indicate that such large-scale genomic studies require collaborations with various groups with expertise in a wide range of research areas. We recognize that some of the deficiencies in the manuscript in the interpretation of the evolution of *B. duncani* might be due to lack of expertise in this specific area of research. We have now sought assistance from colleagues who work in this area and their help was instrumental in updating the tree and interpretations outlined in the revised manuscript.

***Line 429*** *Please do not use Theileridae and Babesidae in this manuscript. It sounds very educated but nobody knows to which group of parasites each term refers (e.g. Babesidae: Babesia microti (Clade I)?, Babesia bovis (Clade VI)?, Babesia duncani (Clade II)?; Theileridae: Theileria equi (Clade IV)? Theileria annulata (Clade V)?). And what about* *Cytauxzoon, which also belong to piroplasmida (Clade III). Each of this species corresponds to another phylogenetic lineages and to allow to distinguish between them they have been designated Clade I to VI.*

**Response:** As suggested by the reviewer, the terminology has been changed from *Theileridae* to *Theleria* spp. and *Babesidae* to *Babesia* spp. to make it easy for the readers to understand which species are used in the analysis (Figure S7). Phylogenetic analysis and OrthoMCL annotation both suggest that Cytauxzoon is at the basal lineage in the *Theileria* spp. including *T. equi*. Further investigations will be needed to investigate the relationship between Cytauxzoon specific markers and evolution of piroplasmida. Our analysis confirmed that this species is in a different clade from *B. duncani*. This was also added and described in the manuscript (page 26).

*Line 534 Delete: “Created with Biorender.com”*

**Response:** Deleted.

*Line 561 Change “Babesia” into italics*

**Response:** Corrected throughout the manuscript.

**Reviewer #3:**

*The manuscript by Lonardi et al, ‘Decoding the nuclear genome of the human pathogen Babesia duncani shed light on its virulence, drug susceptibility and evolution among Apicomplexa’ is a new, detailed genomic study of an emerging zoonotic pathogen transmitted by ticks. I would like to commend the authors on the high quality (PacBio and HiSeq) genome and transcriptome data as well as the use of cutting-edge chromatin structure resolution techniques. Having good ‘omics data on emerging pathogens is critical to understand and tackle the diseases they cause. These ‘omic approaches described in the manuscript have generated a large pool of data rich for interpretation, and for drawing comparison to related pathogens in the phylum of Apicomplexa. A thorough exploration from multi-gene families to energy generation based on transcription data, to potential drug target discovery followed. A wealth of information has been described in this manuscript exploring a novel human*

*pathogen.*

**Response:** We would like to thank Reviewer #3 for this feedback and thorough review for our submitted manuscript.

*Major comments:*

*Fig 3A: representation is too small and lacks for that reason clarity. Maybe a representation as table would be more helpful. Given that both unique and conserved genes are distributed across all chromosomes the purpose of a visual representation seems unnecessary.*

**Response:** We have generated and included a new Figure 3A in the revised manuscript and the data are presented in format that is accessible to all readers as requested by the reviewer.

*•Fig3B: Venn diagram is too complicated and figure legend is not helpful in interpreting the diagram. Of the 1943 orthologues genes, what does the figure of 155 present in the B.bovis only circle? And what does the figure in brackets below the species name mean? What do the 5, 7, 4, 15 etc mean and to which cross-section do they belong. This data needs clarification and simplification. Same goes for Fig3C.*

**Response:** The figure has been replaced with a new figure that is easier to visualize and interpret (new Figure 3A).

*•Fig3D: correct multiple errors relating to chromosome numbers and potentially #genes.*

**Response:** The number of chromosomes in various species are correct based on some of the recent publications.

*•Fig 4: increase species and chromosome numbers on outer circle so that they become legible.*

**Response:** Done.

*•Fig5 => orthologue groups:*

*Lack of description of orthologue groups, and which genes belong two which group in the supplementary Excel file needs correction. A column within the Excel file with gene IDs of different species’ orthologues should also be provided in the supplementary. In addition any information of protein localisation, predicted or confirmed protein function of orthologues from different species should be listed here too.*

**Response:** The additional Excel file contains more than 4,200 annotated genes prediction from the *B. duncani* genome information which will be released when the manuscript is published. We agree with Reviewer #3 about the importance of protein localization and function with respect to the orthologues from different species. We would like to indicate that Dr. Omar Harb (EuPathDB) is one of the co-authors on the manuscript and his team will soon be uploading the data to EuPathDB where most of the analyses described by the reviewer will be easy to mine and will allow reader to select those proteins on the basis of specific criteria such as signal and transit peptides, transmembrane domains, putative cellular localization, expression levels to name only a few. The EupathDB option is a much more efficient way to mine the data and we will make sure that this information becomes available as soon as the manuscript is published.

*Fig5A: what is the x-axis dimension? Level of transcription?*

*Gene product models of different orthologue families should be provided. What do the 51 genes in UMGF1 have in common? Graphical representation might be the best way to depict this.*

**Response:** The x-axis represents the number of unique multigene families of *B. duncani* (BdUMGFs). Considering the space limitation, it is not feasible to show the information on level of transcription of these genes in the same figure. However, we have generated a separate excel file that lists the members of the BdUMGFs (column 1 denotes different BdUMGFs and column 2 depicts gene IDs) and their corresponding transcription levels (TPM values (column 3)).

*oFig5C is too small. Also where are the genes belonging to UMGF2 on the graphic? GPI-anchored gene products are unnecessarily displayed here, I would argue. The distribution of GPI-anchored gene products across all chromosomes is not really surprising and does not need emphasis. Maybe to allow better visualisation, the authors could provide this graphical information of gene locations on chromosomes in a larger, separate supplementary figure, linked to the Excel sheet?*

**Response:** The genes belonging to UMGF2 are on chromosome V. This information has been emphasized in the revised manuscript. Moreover, the distribution of GPI-anchored genes was separated into Figure S5 for better representation.

*Genes in the BdUMGF family are not only in closed proximity to each other at the chromatin 3D level…” should be visually demonstrated on a chromatin blot based on the colours chosen for the families in Fig5. Are dark blue centrally localised UMGF3 genes adjacent to red subtelomeric UMGF1 genes in this chromatin blot given they are both transcribed at low level? Where in the chromatin structure of the nucleus are these gene families located? DNA and RNA-Fish data would also have been useful here.*

**Response:** We thank the reviewer for the suggestion, and we have now conducted DNA fluorescent in-situ hybridization (DNA-FISH) experiments with *B. duncani* using probes specific to UMGF1 (Chr1) and UMGF3 (Chr3) genes (new Figure 6B). Our results indicate that those multigene families are found in distinct chromosomal locations, consistent with our previous prediction. Furthermore, we have generated an additional chromatin 3D model, showing only the chromosomes I, III and IV (Figure 6A and S4).

With regards to transcription repression, we have conducted ChIP-Seq analysis, which showed that even though BdUMGF3 family members are present far from the telomeric or centromeric regions, these genes experience transcriptional repression. The RNA expression data indicate that none of the members of this family are expressed in the blood stage of the parasites and are transcriptionally silenced. It is possible that this multigene family is expressed in the tick or other mammalian hosts.

*Considering the similarity observed between the var and BdUMGF gene families at the chromatin 3D and gene expression level, it is likely that genes that belong to BdUMGF are involved in antigenic variation…” is a strong statement, based a limited data presented here. This needs experimental validation. Do the authors say that all BdUMGF families are transcriptionally repressed? Where can the reader see this in Fig 5A? Do the gene products show similarities across the different UMGF groups and do they have conserved features when looking at well characterised genes involved in antigenic variation (var, vir, Pfam2TM, ves1)?*

*To speculate on antigenic variation based on the subtelomeric location of gene families and low transcription levels is overinterpreted and needs to be substantiated experimentally. For example, single cell RNAseq on B.duncani isolated from hamsters would be able to demonstrated single, or small numbers of genes from individual UMGF groups transcribed and which genes are transcribed would be different in different cells. I would encourage the authors to conduct these experiments to prove their hypothesis of antigenic variation in B. duncani.*

**Response:** Following review #3 comments, we have performed an extensive ChIPSeq analysis on *in vitro* culture of *B. duncani* parasites (Figure 8). The ChIPSeq analysis data of *B. duncani* presented similar trends to the ones observed in other parasites while revealing novel epigenetic insights into babesia gene regulation with multigene families most likely involved in antigenic variation. This analysis was further described in the Results (page 20-21), Discussion (page 27-28), and Methods (pages 47-50) sections.

*Lines 356-359: discusses gene repression on telomeric ends of chr 1, 3, 4 and 5 and links this with the localisation of genes belonging to the BdUMGF1 multigene family. However, there appears to be no UMGF1 gene family members localized to chromosome 3 so why is the repression seen there? Could it be that there is generally repression of transcription in genes localised in telomeric regions rather than the repression being linked to genes hypothesised to be involved in antigenic variation?*

**Response:** We thank the reviewer for pointing out that there are no BdUMGF1 members on chromosome III. We have corrected the statement to exclude chromosome III from this hypothesis. Regarding the 2^nd^ point about gene repression, in many apicomplexan parasites that possess multigene families located either near the telomeric or centromeric regions, gene repression has been reported. However, there are a few exceptions such as in *P. falciparum* and *P. knowlesi* where gene repression has been reported even in the internal *var* gene clusters.

*Minor Comments:*

*-Line 156 – Table I is missing.*

**Response:** Corrected.

*-Line 202 reads Fig3D but should be Fig3E.*

**Response:** Corrected.

*-Lines 216-17: large synteny is between Bd Chr4 and B. bigemina chr1 and not chr 4 as stated.*

**Response:** Corrected.

*-Line 288: what does ‘var gene family consists of ~7x genes’ mean? Rephrase.*

**Response:** That for noticing this error. The correct number (60) has now been entered in the revised version of the manuscript.

*-Lines 317-18: BdUMGF9 group is not indicated in Fig5 which is listed here.*

**Response:** BdUMGF9 is indicated in fig 5A (column number 7).

*-Line 426: there is no phylogenetic tree in Fig S3.*

**Response:** The phylogenetic tree is in Fig S3 panel B.

*-Fig 8F: explain in figure legend the confidence interval (+-) in the enzyme kinetics data.*

**Response:** The confidential interval was added to the figure legend (CI= 95%).

**Reviewer #4:**

*While this manuscript presents new and interesting data on the genomic aspects of Babesia duncani there are numerous inconsistencies, grammatical errors, and poorly described methods and results unbefitting this journal. Please see my specific comments to the pdf manuscript and additional line comments.*

**Response:** We would like to thank reviewer #4 for the extensive and thorough feedback on our manuscript. We have addressed the reviewer notes and comments and included new information incorporated in the manuscript and figures files.

*Grammatical errors have been added to the pdf version in red color. Please be consistent in using the Oxford comma, the meaning of the sentence is altered without it.*

**Response:** Corrected.

*Introduction: Can the authors provide details about what is known on the genetic similarities and differences between Babesia species? A recent review from Goethert 2021, might provide some helpful information.*

**Response:** We would like to thank Reviewer #4 for this note and the proposed review, which we now cite in the manuscript, is indeed very helpful for *B. microti* diversity (Goethert 2021). A revised Figure 3 now addresses this specific comments with 3A showing major similarities and differences between the parasites. Furthermore, in Table 2 we provide genomic data, which include comparisons between *B. duncani* and other parasites including *B. microti* (human parasite) and *B. bovis* (bovine parasite).

*Line 57: Please be specific about which species of Babesia is being referenced here with the 9% mortality rate. Reference 4 is specific to B. microti.*

**Response:** We have modified the statement to include a recent epidemiological study on hospitalized human babesiosis patients. Moreover, we have indicated in line 66 that the dominant species associated with human Babesiosis cases and mortality rate in North America is *B. microti*.

*Line 84: Suggest starting a new paragraph with “Despite the highly…”*

**Response:** Done.

*Line 97: Spell out the genus name at the beginning of a sentence.*

**Response:** Done.

*Line 97-100: It would be helpful to explain the overall results here instead of listing the items depicted in Fig 1. Can the results in Fig 1 be explained a bit more here?*

**Response:** Thanks for pointing this out. The parasite life cycle has been explained in the results as depicted in Fig 1.

*Line 128: The last part of this sentence seems out of place. It would be better to end the sentence after reference 21 and begin a new sentence about the centromeres.*

**Response:** Corrected.

*Line 128: What “feature” are the authors referring to? Many features were stated in the previous sentence, please be specific which one or ones this sentence is regarding. Separating the centromere comment from the previous sentence will also make it clearer that this is the feature that is important for the following sentence.*

**Response:** This sentence has now been corrected in the revised version of the manuscript as suggested by the reviewer

*Line 129-130: Above the chromosomes are listed using roman numerals, please be consistent here and use roman numerals for chromosome number.*

**Response:** Corrected.

*Line 129-131: Can the types of centromeres be explained more? What is the difference and the significance of these three types of centromeres?*

**Response:** The types of the chromosomes have been explained with respect to the position of the centromere on the chromosomal arm.

*Line 140: Spell out the genus name the first time it is used.*

**Response:** Done.

*Line 174: Spell out the genus name the first time it is used.*

**Response:** Done.

*Lines 173-177: Would be better suited in the Introduction.*

**Response:** The observation that *B. duncani* has 37.32% GC content is a result from the genome sequencing and not based on previous knowledge. Therefore we have included this information and the associated statement under the result section.

*Line 179: Suggest rewording the beginning of this sentence so the reader doesn’t feel commanded. Some examples: “We observed…” or “The genome size…was higher in B. duncani compared to…”*

**Response:** Corrected.

*Line 189: Bold Fig 3A to be consistent.*

**Response:** Corrected.

*Line 195 and 196: orthologous groups?*

**Response:** Corrected.

*Line 198: Capitalize Apicomplexa*

**Response:** Done.

*Lines 200-201: What do the authors mean by “gene content did not reveal any surprises”? Please use more technical language. What constitutes a “surprise” for the authors?*

**Response:** Rephrased as follows: Gene content did not reveal any surprises and largely correlated with genome size

*Lines 211-217: Be consistent with chromosome numbering, roman numerals*

**Response:** Done.

*Line 219: Italicize genus name*

**Response:** Done.

*Line 231: Do not capitalize lactate*

**Response:** Done.

*Line 236: Spell out CDS at first use*

**Response:** Corrected.

*Line 239: Numbers should be spelled out when they start a sentence.*

**Response:** Done.

*Lines 242-245: The authors state that 4 aspartic proteases are expressed in P. falciparum and B. duncani, are they the same aspartic proteases in both species? The following sentence lists 4 proteases and their function, but are these the same ones expressed by P. falciparum?*

**Response:** As mentioned in lines 255-259, of the 4 asexually expressed aspartic proteases present in *B. duncani*, 3 of them shares homology with different *P. falciparum* plasmepsins. BdWA1_003366 shares ~45% identity with plasmepsins IX and X. BdWA1_00223 shares sequence similarity with plasmepsin V, and BdWA1_001984 shares homology with all the *P. falciparum* aspartic proteases (23-27% identity).

*Lines 267 and 270: Apicomplexa should not be italicized*

**Response:** Corrected.

*Line 271: Do not italicize “spp”*

**Response:** Corrected.

*Line 273: Spell out genus the first time it’s used*

**Response:** Corrected.

*Line 276 and 279: Be consistent with figure abbreviations (Fig. X). Also, spell out GPI and AP families on the first use.*

**Response:** Corrected.

*Line 276: See above comment about starting a sentence with a command (Observe…)*

**Response:** Corrected.

*Lines 278-282: Chromosome numbering*

**Response:** Corrected.

*Line 283: Do the authors mean to say “close proximity”?*

**Response:** Yes, corrected.

*Line 285: Bold Fig reference to be consistent*

**Response:** Corrected.

*Line 289-292: References are needed for the information about the var gene family in P. falciparum.*

**Response:** Added.

*Line 291: Do the authors mean to say “structural changes”?*

**Response: Yes, corrected.**

*Line 286-292: Please be consistent and pay close attention to how you are writing genes and gene families (var, Var, var)*

**Response: Corrected.**

*Line 298: Spell out MSP the first time it’s used*

**Response: Corrected.**

*Line 306: Add references for this information (B. microti ortholog and conserved region in other piroplasmida)*

**Response: Added.**

*Lines 309-311: Add references for this information*

**Response: Added.**

*Line 312: Apicomplexa should not be italicized*

**Response: Corrected.**

*Lines 297-317: Several sentences in this paragraph would be better suited for the Discussion section.*

**Response:** Thanks for the suggestion. We believe that the sentences in this paragraph are needed in this section because they are directly linked with the findings.

*Line 322: Please clarify what is meant by “…tick vector families…” Ticks belong to either the Ixodidae or Argasidae family, but I’m guessing the authors mean gene families. Please reword the sentence to make this more clear.*

**Response:** By tick vector families, we mean tick vectors. The term family has been removed to clarify the sentence.

*Lines 327-329: Please clarify how capturing almost 90% of the genome indicates that most genes are needed for survival in host RBCs. How do you know some of these genes aren’t needed to survive in the tick? Or for transmission from tick-to-host? Seq should not be capitalized.*

**Response:** Clarified. The expression of 90% of the predicted genes during the asexual life cycle stage indicates that these genes may play role in parasite survival within the host red blood cells (RBCs). However, it is also possible that a small number of asexually expressed genes might be important for parasite life cycle in the tick vector.

*Line 339: Please be consistent with narrative, third person has been used throughout the manuscript however fist person (we) is used here.*

**Response:** Corrected.

*Line 341: Capitalize and italicize genus name*

**Response:** Corrected.

*Line 343: Clarify which “genes” are being reference here*

**Response:** Done.

*Line 344: lncRNAs should be spelled out the first time it is used, not the second.*

**Response:** Corrected.

*Line 347: Reference needed*

**Response:** Added.

*Line 347: What is “their roles…” referring to? lncRNAs? It would be better to say “The roles of lncRNAs have…”*

**Response:** Corrected.

*Line 349: The narrative has changed. It seems someone else wrote this section, please be consistent with tense and narrative throughout the manuscript.*

**Response:** Corrected.

*Line 354: Do not italicize “the”*

**Response:** Corrected.

*Lines 358-359: Chromosome numbering*

**Response:** Corrected.

*Line 372: Reference needed*

**Response:** Added.

*Line 384: Bold Fig. for consistency*

**Response:** Corrected.

*Line 390: Reference needed*

**Response:** Statement has been modified and the required reference is added.

*Line 401: Do the authors mean “studies”*

**Response:** Yes, corrected.

*Line 405: Please clarify who “they” is in this sentence.*

**Response:** Done.

*Line 416: References needed*

**Response:** Added.

*Line 417: Italicize genus name*

**Response:** Corrected.

*Line 419: References needed*

**Response:** Statement has been modified and the required reference is added.

*Line 423: Apicomplexa should not be italicized*

**Response:** Corrected. Moreover, the term apicomplexan has been replaced by piroplasmids.

*Line 430: References needed*

**Response:** Corrected.

*Line 431: References needed*

**Response:** Corrected.

*Line 435: Spell out RAP upon first use*

**Response:** Corrected.

*Line 437: Spell out numbers when they start a sentence*

**Response:** Corrected.

*Line 449: Spell out TFIIA upon first use*

**Response:** Corrected.

*Line 483: Don’t italicize sp.*

**Response:** Corrected.

*Line 512: Spell out GO upon first use. Too many spaces in front of GO?*

**Response:** Corrected.

*Line 517 and 519: Italicize genus name*

**Response:** Corrected.

*Line 545: Do not italicize “and”*

**Response:** Corrected.

*Line 552: Spell out Bd and Bm, I assume these are B. duncani and B. microti, but these abbreviations have not been used previously.*

**Response:** Done.

*Line 561: Italicize genus name*

**Response:** Corrected.

*Materials and Methods: It is best to use past tense because the experiments have been completed.*

**Response:** Corrected.

*Line 579: Add a space between B. duncani*

**Response:** Corrected.

*Line 586: Suggest rewording the sentence as “…changed daily and parasitemia was monitored by…”*

**Response:** Corrected.

*Line 590: 95% pure what? Please clarify. Do the authors mean to say “purity”? Or change “with” to “were”*

**Response:** Corrected.

*Line 605: Do the author mean to say “move” instead of movie?*

**Response:** Yes, corrected.

*Line 607: Explain what “Yale’s gold standard” is, what are the measurements?*

**Response:** The parameters for Yale’s gold standard were added in the revised manuscript. Briefly, Yale gold standards refer to polymerase read length between 50-60kb, data yield (HiFi) around 2-4 million reads of total 10-20Gb, and P1 between 60-70%.

*Line 609: Please explain the frozen pellets. Was B. duncani frozen into pellets and packed into 3ml packaging? Why? How were these frozen pellets of B. duncani used to isolate molecular weight? Please be clear about this method.*

**Response:** The method has been edited for clarity.

*Lines 624-629: A lot of this seems like Results.*

**Response:** The data provided here is supporting the PacBio HiFi whole genome sequencing method applied in this study. Nevertheless, this data was added to the results section as suggested by the reviewer.

*Line 677: PASTIS should be in all capital letters*

**Response:** Corrected.

*Line 682: It looks like reference 42 is in a different font.*

**Response:** Corrected.

*Line 695: Replace ran with run*

**Response:** Done.

*Line 707: Why is translated capitalized? Numbers should be spelled out when at the beginning of a sentence. I suggest rewording this sentence.*

**Response:** Done.

*Line 720: Suggest rewording “Babesia microti drastically reduced…”*

**Response:** Corrected.

*Lines 736-754: Can this section be combined with the 3D modeling section on lines 673-679? If not, please explain the differences between these two 3D modeling sections.*

**Response:** Combined.

*Lines 762-763: Poorly worded sentences, please reword.*

**Response:** Rephrased.

*Line 767: Please be consistent, intraerythrocytic was not hyphenated previously.*

**Response:** Corrected.

*Line 768: Please be consistent, references should not be italicized.*

**Response:** Corrected.

*Line 770: Do not capitalize hrs. Please be consistent with the units, h, hrs, hr, hours have all been used.*

**Response:** Corrected.

*The authors state in the Abstract that they performed “efficacy studies” however this was not explained in the results. How did the authors challenge a host for the efficacy studies? What host was used? Where are the details about these studies and the results? In the last section of the Results, the authors used human cell lines, but not live hosts. In the Discussion, they say their results could be used to test efficacy in mice. Please clarify if these studies were conducted and provide better methods and results of these trials.*

**Response:** The efficacy studies here refer to *in vitro* efficacy studies using a continuous in vitro culture system of *B. duncani* infected human RBCs. The statement in the abstract has been corrected to include “*in vitro*” before the efficacy studies. The detailed method is provided in page 51. In the last section of the results, we state that the human cell lines were used to perform drug cytotoxity assays. A detailed method is provided in page 52. In the discussion, we state that the *in vitro* drug and safety data will guide future studies in mice to evaluate the efficacy of the compounds in vivo. These studies, however, require that specific criteria for safety and tolerability in mice be met first before a compound can be evaluated for its antibabesial activity in animals. We anticipate conducting such studies in the future with analogs of WR99210 that are considered safe.

*Highlighted sentences in the Results section need to be better addressed and explained in the Discussion section.*

**Response:** No document with highlighted sentences was provided with the editor’s email.

*The authors should be consistent with how they reference the figures in the figure legend. Sometimes a period is used, sometimes a colon, and sometimes a single parenthesis (A. text. B: text. C) text.).*

**Response:** Corrected.

*Please check the references as several have mistakes (i.e. scientific names not italicized).*

**Response:** Corrected.

*Figures 3, 4, 5: The font size is too small in several figures making it difficult to read.*

**Response:** Corrected.

*Table 6: Is the SP/TM column necessary to include as no data is presented in this column? Could this be better as a footnote?*

**Response:** Thanks for the suggestion, the SP/TM have been moved to the footnote.

*Figures S3: Scientific names should be italicized*

**Response:** Corrected in Figure S7 (old Figure S3).

Once again, we thank the reviewers for the excellent comments, which have helped us enhance the standard of this work. I hope you find this revised manuscript acceptable for publication in Nature Microbiology.

Sincerely,

**Choukri Ben Mamoun**

Yale School of Medicine

Departments of Medicine and Microbial Pathogenesis

Section of Infectious Diseases

300 Cedar Street

New Haven, CT 06520

choukri.benmamoun@yale.edu

Phone: +1 203-737-1972

**Decision Letter, first revision:**

| **Message:** | Our ref: NMICROBIOL-22051126B  30th January 2023  Dear Choukri,  Thank you for submitting your revised manuscript "Decoding The Nuclear Genome of The Human Pathogen Babesia duncani Sheds Light on its Virulence, Drug Susceptibility and Evolution within Apicomplexa" (NMICROBIOL-22051126B). It has now been seen by the original referees and their comments are below. The reviewers find that the paper has improved in revision, and therefore we'll be happy in principle to publish it in Nature Microbiology, pending minor revisions to satisfy the referees' final requests and to comply with our editorial and formatting guidelines.  We are now performing detailed checks on your paper and will send you a checklist detailing our editorial and formatting requirements in about a week. Please do not upload the final materials and make any revisions until you receive this additional information from us.  Thank you again for your interest in Nature Microbiology Please do not hesitate to contact me if you have any questions.  [redacted]   Reviewer #1 (Remarks to the Author):  The authors have carried out substantial further work to address my main request for further information regarding the species-specific genes they have discovered. They have shown that, in keeping with the familiar situation in comparable parasites, transcription of B. duncani-specific gene families is likely regulated epigenetically through chromatin organisation. This is a valuable addition and certainly establishes B. duncani among the best understood piroplasm genome sequences. The study firmly establishes the unique character of the B. duncani genome, including the absence of VESAs, and its phylogenetic distinctiveness. This last point makes me wonder how well it can perform as a model for other piroplasms, when many of the genetic determinants of their pathogenic mechanisms are missing or diverged in nature.   Reviewer #2 (Remarks to the Author):  First of all the manuscript has much improved. However, there are still some flaws that need to be addressed. General comments Authors need to make reference to a recent publication that analyses various aspects of the B. duncani genome. One of these is that Lonardi et al. finds the same tree topology described in Wang et al. 2022, who also carries out a phylogenomic approach based on multiple orthologous genes. Lonardi et al. would strengthen their findings which confirm the tree topology reported by Wang et al. There are also a number of other findings on the B. duncani genome reported in Wang et al and authors should make reference whether they confirm or contradict their findings (Wang J, Chen K, Yang J, et al. Comparative genomic analysis of Babesia duncani responsible for human babesiosis. BMC Biol. 2022;20:153). Specific comments Line 57 to 60 multiple orthographic mistakes an gramar mistakes are in this sentence Line 71 Although still sometimes used instead of 18S rDNA the designation 18S rRNA gene is much preferred because DNA is not a product of this gene and rDNA does not exist. Line 85 “respiratory distress syndrome” should be written in lower case letters Line 91 change to “…and identified localization of active and inactive histone marks.” Line 94 change to “…during this phase of parasite development…” Line 98 “…that undergo antigenic variation in other Apicomplexa.” Comment: Please be more specific, with regard to which genera of apicomplexa do antigenic variation. As to my understanding it has been only described in Haemosporidiia, that is, Plasmodium (e.g. Plasmodium falciparum) and in Babesia (e.g. Babesia bovis). So why including all other apicomplexa genera such as Toxoplasma, Sarcocystis, Eimeria etc, etc. where this phenomenon has not been described? Line 99”…identified antifolates, pyrimethaminem,…” Line 222 It is of high importance that Material and Methods are described in sufficient detail so that the scientific community can carry out the described experimental procedure and analysis. Only this will allow to test and challenge reported results. It is therefore indispensable to include to the manuscript a list of the 191 single copy orthologous protein sequences used for alignment and tree building as supplementary data. Line 335 Authors should here make reference to the report of the complete GPI-anchored proteome of Babesia bovis (Rodriguez et al. Ticks Tick Borne Dis. 2014, 5: 343-8). It has been the first study in which the complete GPI-anchored genome of a Babesia species has been identified by bioinformatic analysis using a novel approach. Second because this species is of high veterinary importance and deserves to be mentioned in this context. Third because it is of interest to see whether the GPI-anchored proteome between Babesia spp. of different phylogenetic lineages is conserved (or polymorphic).    Reviewer #3 (Remarks to the Author):  I would like to thank the authors of this manuscript for their additional work both experimentally and in modifying their original manuscript in text and figures which has resulted in a much improved version.  • Line 135 (Chromosome IIII – change to III)  • FISH experiments lines 322-326: the authors should show UMGF1 probe 1 (chr1) and 2 (chr4) do overlap.  • Line 347 chemical reaction was lost, please add in again.  • Fig7: in text panel D is mentioned before panel C, hence reorder figure OR as the figure order makes more sense in its current configuration, reorder the text to allow mentioning of Fig7C before Fig7D?  • Line 432: What are the authors’ suggestions to the role of genes on the right hand arm of ChrIII in the sub-telomeric region associated with a large patch of H3K9me3 marks? I am not sure I understand the hypothesis here. Given these genes are not part of a multigene family, antigenic variation cannot be the purpose.  • Line 439 “… most likely involved in antigenic variation”. The ChIP-Seq data is very convincing and a valuable addition to the manuscript and our knowledge in gene regulation of Babesia parasites. But although there is a significant association with multi-gene families and H3K9me3 marks, and therefore the proof of epigenetics involved in the regulation of these genes, no proof of mutual exclusive expression has been demonstrated as would be possible using single cell RNA-seq. Therefore, I would strongly recommend to the authors to turn down the wording here about what consequences the H3K9me3 marks result in (line 439). The authors have however found the right balance in the discussion section (line 593). |
| --- | --- |

**Author Rebuttal, first revision:**

**Reviewer #1 (Remarks to the Author):**

The authors have carried out substantial further work to address my main request for further information regarding the species-specific genes they have discovered. They have shown that, in keeping with the familiar situation in comparable parasites, transcription of *B. duncani*-specific gene families is likely regulated epigenetically through chromatin organisation. This is a valuable addition and certainly establishes *B. duncani* among the best understood piroplasm genome sequences. The study firmly establishes the unique character of the *B. duncani* genome, including the absence of VESAs, and its phylogenetic distinctiveness. This last point makes me wonder how well it can perform as a model for other piroplasms, when many of the genetic determinants of their pathogenic mechanisms are missing or diverged in nature.

**Response**: We thank the reviewer for the feedback and thorough review of the manuscript. While *B. duncani* has evolved certain unique multigene families (UMGFs) that makes it different from other piroplasm, it has also other multigene families (OMGFs: multigene families with orthologs in other Apicomplexa) that are shared with other piroplasms. What is important in the case of both multigene families is how *B. duncani* uses them to interact with the host, a question that we hope the parasitology community will be able to investigate and answer in the future. The ability of *B. duncani* to propagate continuously in vitro in human red blood cells and its ability to cause lethal infection in mice makes it a particularly attractive system to study several other aspects of its biology such as its nutrient requirements, metabolic functions, and the biogenesis of its organelles. Using this model system, we are now able to evaluate the importance of genes in parasite development in vitro and assess their importance in virulence in mice.

**Reviewer #2 (Remarks to the Author):**

First of all the manuscript has much improved. However, there are still some flaws that need to be addressed.

**General comments**

Authors need to make reference to a recent publication that analyses various aspects of the *B. duncani* genome. One of these is that Lonardi et al. finds the same tree topology described in Wang et al. 2022, who also carries out a phylogenomic approach based on multiple orthologous genes. Lonardi et al. would strengthen their findings which confirm the tree topology reported by Wang et al. There are also a number of other findings on the *B. duncani* genome reported in Wang et al and authors should make reference whether they confirm or contradict their findings (Wang J, Chen K, Yang J, et al. Comparative genomic analysis of *Babesia duncani* responsible for human babesiosis. BMC Biol. 2022;20:153).

**Response:** A reference to this paper has been added in the Discussion section of the manuscript. We do note, however, that our work was posted on BioRXiv earlier than the BMC Biology paper. In terms of assembly quality, the BMC Biology assembly is (1) more fragmented than ours (we have N50 of 1.4Mb, their N50 is only 1Mb), (2) less complete than ours (total assembled size of ours is 10.4Mb, theirs is 7.9Mb).

Furthermore, the paper by Wang and colleagues lacks all the following:

- No evidence that the genome consists of 5 chromosomes, which we confirmed using PFGE, HiC and optical mapping.
- No comparative analyses to determine how the evolution of this parasite among Apicomplexa impacted its cellular and metabolic functions
- No evidence of multigene families in *B. duncani*, which we further supported by HiC and FISH.
- No information about the 3D conformation of the *B. duncani* genome
- No RNAseq analysis, which in our case revealed which metabolic functions are essential for parasite survival and provided evidence that the parasite likely undergoes antigenic variation.
- No ChIP analysis, which showed for the first time that the parasite regulates the expression of a specific set of genes, which are likely to be involved in antigenic variation and/or sexual differentiation.
- No reconstitution of the parasite metabolic functions
- No translation of the genomic data into knowledge that could guide therapy of human babesiosis.

**Specific comments**

Line 57 to 60 multiple orthographic mistakes an grammar mistakes are in this sentence

**Response:** We thank Reviewer #2 for bringing this to our attention. The sentence has been modified to correct the grammar.

Line 71 Although still sometimes used instead of 18S rDNA the designation 18S rRNA gene is much preferred because DNA is not a product of this gene and rDNA does not exist.

**Response:** Corrected.

Line 85 “respiratory distress syndrome” should be written in lower case letters

**Response:** Corrected.

Line 91 change to “…and identified localization of active and inactive histone marks.”

**Response:** Done.

Line 94 change to “…during this phase of parasite development…”

**Response:** Done.

Line 98 “…that undergo antigenic variation in other Apicomplexa.” Comment: Please be more specific, with regard to which genera of apicomplexa do antigenic variation. As to my understanding it has been only described in Haemosporidiia, that is*, Plasmodium* (e.g. *Plasmodium falciparum*) and in Babesia (e.g. *Babesia bovis*). So why including all other apicomplexa genera such as *Toxoplasma*, *Sarcocystis*, *Eimeria* etc, etc. where this phenomenon has not been described?

**Response:** We thank the reviewer for pointing this out. The statement has been modified to include Hemosporida instead of Apicomplexa.

Line 99”…identified antifolates, pyrimethaminem,…”

**Response:** Thanks for pointing out, but it appears that the submitted version already has correct names (“pyrimethamine” and not “pyrimethaminem”).

Line 222 It is of high importance that Material and Methods are described in sufficient detail so that the scientific community can carry out the described experimental procedure and analysis. Only this will allow to test and challenge reported results. It is therefore indispensable to include to the manuscript a list of the 191 single copy orthologous protein sequences used for alignment and tree building as supplementary data.

**Response:** This was an omission from our part, and we have now uploaded the file and the ID of the 190 proteins used to construct the tree as requested by the reviewer.

Line 335 Authors should here make reference to the report of the complete GPI-anchored proteome of *Babesia bovis* (Rodriguez et al. Ticks Tick Borne Dis. 2014, 5: 343-8). It has been the first study in which the complete GPI-anchored genome of a *Babesia* species has been identified by bioinformatic analysis using a novel approach. Second because this species is of high veterinary importance and deserves to be mentioned in this context. Third because it is of interest to see whether the GPI-anchored proteome between *Babesia* spp. of different phylogenetic lineages is conserved (or polymorphic).

**Response:** We thank the reviewer of this suggestion. We have incorporated the suggested reference to the manuscript.

**Reviewer #3 (Remarks to the Author):**

I would like to thank the authors of this manuscript for their additional work both experimentally and in modifying their original manuscript in text and figures which has resulted in a much improved version.

• Line 135 (Chromosome IIII – change to III)

**Response:** Done.

• FISH experiments lines 322-326: the authors should show UMGF1 probe 1 (chr1) and 2 (chr4) do overlap.

**Response:** We thank the reviewer for the suggestion and we have now conducted the study as requested and added the data to Figure 4B.

• Line 347 chemical reaction was lost, please add in again.

**Response:** Added.

• Fig7: in text panel D is mentioned before panel C, hence reorder figure OR as the figure order makes more sense in its current configuration, reorder the text to allow mentioning of Fig7C before Fig7D?

**Response:** Thanks for bringing this to our attention. We have modified the text to mention Fig. 7C before 7D.

• Line 432: What are the authors’ suggestions to the role of genes on the right hand arm of ChrIII in the sub-telomeric region associated with a large patch of H3K9me3 marks? I am not sure I understand the hypothesis here. Given these genes are not part of a multigene family, antigenic variation cannot be the purpose.

**Response:** While these genes are not part of a multigene family that have been identified using our standard pipeline, these genes are associated with H3K9me3 marks are most likely epigenetically regulated. They could therefore be involved in host-pathogen interactions and/or sexual differentiation.

• Line 439 “… most likely involved in antigenic variation”. The ChIP-Seq data is very convincing and a valuable addition to the manuscript and our knowledge in gene regulation of *Babesia* parasites. But although there is a significant association with multi-gene families and H3K9me3 marks, and therefore the proof of epigenetics involved in the regulation of these genes, no proof of mutual exclusive expression has been demonstrated as would be possible using single cell RNA-seq. Therefore, I would strongly recommend to the authors to turn down the wording here about what consequences the H3K9me3 marks result in (line 439). The authors have however found the right balance in the discussion section (line 593).

**Response:** We agree with the reviewer that further studies are needed to confirm the mutually exclusive nature of the genes involved in antigenic variation. Nevertheless, our ChIP seq data provide sufficient data supporting this process and a good starting point for researchers interested in studying antigenic variation in *B. duncani*. As suggested by the reviewer, we have modified the text in line 439.

**Final Decision Letter:**

| **Message:** | 14th March 2023  Dear Dr Ben Mamoun,  I am pleased to accept your Article "Babesia duncani multi-omics identifies virulence factors and drug targets." for publication in Nature Microbiology on behalf of [redacted] who is out of the office. Thank you for having chosen to submit your work to us and many congratulations.  Over the next few weeks, your paper will be copyedited to ensure that it conforms to Nature Microbiology style. We look particularly carefully at the titles of all papers to ensure that they are relatively brief and understandable.  Once your paper is typeset, you will receive an email with a link to choose the appropriate publishing options for your paper and our Author Services team will be in touch regarding any additional information that may be required. Once your paper has been scheduled for online publication, the Nature press office will be in touch to confirm the details.  After the grant of rights is completed, you will receive a link to your electronic proof via email with a request to make any corrections within 48 hours. If, when you receive your proof, you cannot meet this deadline, please inform us at rjsproduction@springernature.com immediately. You will not receive your proofs until the publishing agreement has been received through our system  Due to the importance of these deadlines, we ask you please us know now whether you will be difficult to contact over the next month. If this is the case, we ask you provide us with the contact information (email, phone and fax) of someone who will be able to check the proofs on your behalf, and who will be available to address any last-minute problems.  Acceptance of your manuscript is conditional on all authors' agreement with our publication policies (see https://www.nature.com/nmicrobiol/editorial-policies). In particular your manuscript must not be published elsewhere and there must be no announcement of the work to any media outlet until the publication date (the day on which it is uploaded onto our website).  Please note that <i>Nature Microbiology</i> is a Transformative Journal (TJ). Authors may publish their research with us through the traditional subscription access route or make their paper immediately open access through payment of an article-processing charge (APC). Authors will not be required to make a final decision about access to their article until it has been accepted. <a href="https://www.springernature.com/gp/open-research/transformative-journals"> Find out more about Transformative Journals</a>  **Authors may need to take specific actions to achieve <a href="https://www.springernature.com/gp/open-research/funding/policy-compliance-faqs"> compliance</a> with funder and institutional open access mandates.** If your research is supported by a funder that requires immediate open access (e.g. according to <a href="https://www.springernature.com/gp/open-research/plan-s-compliance">Plan S principles</a>) then you should select the gold OA route, and we will direct you to the compliant route where possible. For authors selecting the subscription publication route, the journal’s standard licensing terms will need to be accepted, including <a href="https://www.nature.com/nature-portfolio/editorial-policies/self-archiving-and-license-to-publish">self-archiving policies</a>. Those licensing terms will supersede any other terms that the author or any third party may assert apply to any version of the manuscript.   If you have any questions about our publishing options, costs, Open Access requirements, or our legal forms, please contact ASJournals@springernature.com  An online order form for reprints of your paper is available at <a href="https://www.nature.com/reprints/author-reprints.html">https://www.nature.com/reprints/author-reprints.html</a>. All co-authors, authors' institutions and authors' funding agencies can order reprints using the form appropriate to their geographical region.  We welcome the submission of potential cover material (including a short caption of around 40 words) related to your manuscript; suggestions should be sent to Nature Microbiology as electronic files (the image should be 300 dpi at 210 x 297 mm in either TIFF or JPEG format). Please note that such pictures should be selected more for their aesthetic appeal than for their scientific content, and that colour images work better than black and white or grayscale images. Please do not try to design a cover with the Nature Microbiology logo etc., and please do not submit composites of images related to your work. I am sure you will understand that we cannot make any promise as to whether any of your suggestions might be selected for the cover of the journal.  You can now use a single sign-on for all your accounts, view the status of all your manuscript submissions and reviews, access usage statistics for your published articles and download a record of your refereeing activity for the Nature journals.  To assist our authors in disseminating their research to the broader community, our SharedIt initiative provides you with a unique shareable link that will allow anyone (with or without a subscription) to read the published article. Recipients of the link with a subscription will also be able to download and print the PDF.   As soon as your article is published, you will receive an automated email with your shareable link.   With kind regards,  [redacted] |
| --- | --- |
